# Synthesis, Antibacterial Properties and Molecular Docking Studies of Nitrogen Substituted 9-(((4X-But-2-ynyloxy)methyl)-1,2,3-triazolyl)–Cinchona Alkaloid Conjugates

**DOI:** 10.3390/molecules30224352

**Published:** 2025-11-10

**Authors:** Gulim K. Mukusheva, Nurizat N. Toigambekova, Victor A. Savelyev, Andrey I. Khlebnikov, Liubov G. Burova, Sofiia D. Afanaseva, Oralgazy A. Nurkenov, Anarkul S. Kishkentayeva, Aikerim S. Olzhabayeva, Yurii V. Gatilov, Roza B. Seidakhmetova, Alexander N. Evstropov, Elvira E. Shults

**Affiliations:** 1 Chemistry Faculty, Karaganda National Research University Buketov, Karaganda 100024, Kazakhstan; nukonti92@mail.ru (N.N.T.);; 2 Vorozhtsov Novosibirsk Institute of Organic Chemistry, Siberian Branch of the Russian Academy of Sciences, Novosibirsk 630090, Russia; 3 Kizhner Research Center, Tomsk Polytechnic University, Tomsk 634050, Russia; aikhl@chem.org.ru; 4 Department of Microbiology, Immunology and Virology, Novosibirsk State Medical University, Krasny Prospect 52, Novosibirsk 630091, Russia; 5 Institute of Organic Synthesis and Coal Chemistry of the Republic of Kazakhstan, Karaganda 100008, Kazakhstan; nurkenov_oral@mail.ru; 6 School of Pharmacy, Karaganda Medical University, Karaganda 100012, Kazakhstan; 7 Department of Clinical Pharmacology and Evidence-Based Medicine, Karaganda Medical University, Karaganda 100012, Kazakhstan; roza77124@gmail.com

**Keywords:** cinchona alkaloids, quinine, triazoles, A^3^-coupling reactions, antibacterial activity, bacterial biofilms, molecular docking study

## Abstract

The year 2024 marked the 80th anniversary of Woodward’s total synthesis of quinine. Quinine is a natural alkaloid from the bark of the cinchona tree that has been used for years as an antimalarial drug. The antibacterial effect of quinine salts has also been regarded. With this in mind, a series of original 9-deoxycinchone alkaloid derivatives bearing a dialkylamino- or heterocyclic moiety at the 4 position of the 9-(((4-X-but-2-ynyloxy)methyl)-1,2,3-triazolyl)-substituent was synthesized. The copper-catalyzed three-component A^3^-coupling reaction of 9-(((4-prop-2-ynyloxy)methyl)-1,2,3-triazolyl)- substituted cinchona alkaloid derivatives with secondary amines and formaldehyde was the main route of synthesis. The present study attempted to examine the antibacterial properties of 9-substituted 9-desoxyquinine-derived compounds and their antibacterial activity against pathogenic bacterial strains, e.g., *Staphylococcus aureus*, *Bacillus subtillis*, *Bacillus cereus*, and *Escherichia coli*. The difference in the antibacterial activity profile of diastereoisomeric 9-(((4-X-but-2-ynyloxy)methyl)-1,2,3-triazolyl)-substituted derivatives of cinchona alkaloids indicated the importance of the nature of nitrogen substituents in the molecules. In a concentration-dependent pattern, (9*R*)- and (9*S*)- (((4-asocan-1yl)-but-2-ynyl-oxy)methyl)-1,2,3-triazolyl)-substituted compounds demonstrated considerable biofilm-inhibitory efficacy against the *S. aureus* bacterial strain. A detailed study of the molecular interactions with the targeted protein MurB was performed using docking simulations, and the obtained results are quite promising.

## 1. Introduction

*Cinchona* alkaloids, natural products typically isolated from the bark of the trees of the genus *Cinchona*, have proven quite versatile. They were used for the treatment of malaria [1,2,3] and cardiac arrhythmias [4] dating from the 17th century. Several studies have revealed that the alkaloids of *Cinchona* bark have other potential activities, such as anticancer, anti-inflammatory, analgesic, and antiviral activities [5,6]. Quinine (8α,9*R*)-6′-methoxycinchonan-9-ol) **1** is the main active ingredient in the bark of *Cinchona*, with a content of up to 3 % (total alkali content of up to 6 %) [7]. Besides its use as the lead compound for the preparation of chiral catalysts, ligands [8,9,10,11,12], and chromatographic selectors [13], quinine **1** has proven to be a valuable building block for synthesizing novel stereochemically enriched bioactive compounds [14,15,16,17,18].

**Figure 1:**
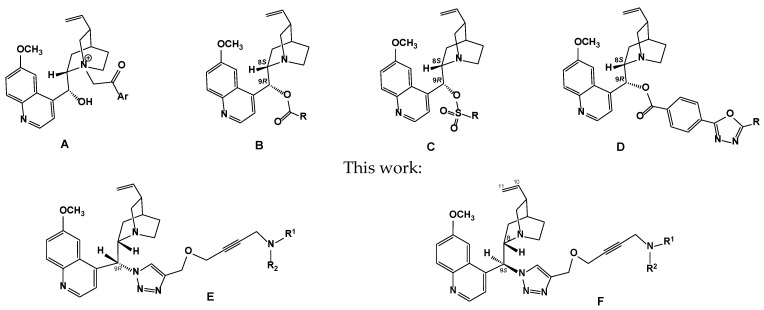
Chemical structures of amphiphilic cationic quinine compounds (**A**), 9-hydroxyl group-substituted quinine derivatives (**B**–**D**), and related nitrogen-substituted 9-(((4-N-but-2-ynyloxy)-melhyl)-1,2,3-triazolyl)-9-deoxy-cinchona alkaloid derivatives (**E**,**F**) synthesized in this work.

Various studies have regarded the antibacterial effects of the alkaloids quinine **1** [19,20,21] and 10,11-dihydroquinine [22] against both Gram-positive and Gram-negative pathogenic microorganisms. The investigation of the effect of quinine sulfate on the growth and invasion of several bacteria showed that the invasive ability of *Enterobacter agglomerans* and *Staphylococcus aureus* was significantly inhibited by 50 and 100 μM [21]. An important advantage of antimalarials is that they do not act directly on the invading pathogens, but rather on the host cells, so that there are few, if any, chances for the microorganisms to become resistant to their effects. Several studies have reported that alkylation on tertiary nitrogen with alkyl (aryl) bromides or iodides [23] or the introduction of functional groups (esterification on C-9 hydroxy group [24]) improved the antibacterial activity of quinine against human pathogenic bacteria strains *Escherichia coli*, *Staphylococcus aureus*, *Pseudomonas aeruginosa*, and *Bacillus substillis*. The minimal inhibitory concentration values in vitro of compound **A** (Figure 1) on methicillin-resistant *S. aureus* range from 0.4 to 1.6 μg/mL (MBC < 3.2 μg/mL) [23]. Quinine esters (especially propionate **B**) (Figure 1) have shown strong inhibition on the growth of *E. coli*, *P. aeruginosa*, and *B. substilis* with an MIC of 0.5–1.25% [24]. It has also been demonstrated that quinine derivatives exhibit antibacterial activity and can inhibit bacterial topoisomerase II (DNA gyrase) [25]. Several dihydroquinine derivatives have been synthesized, and resistance studies, combined with molecular modeling, suggest that these compounds likely interact with the C ring of ATP synthase near the conserved glutamate binding site [26]. Some interesting results of structure–activity relationships have also been observed. It has been documented that a free 9-hydroxyl group is not a prerequisite for activity, and C9-substitution is well tolerated [24,26]. Compounds of quinine sulfonate structure **C**, and also 9*R*-acyloxyquinine derivatives containing 1,3,4-oxadiazole moieties (compounds **D**), have exhibited significant anti-oomycete and anti-fungal activities [27,28]. *Cinchona* alkaloids have been modified by click chemistry utilizing the reaction of 9-azidoderivatives with different functionalized terminal alkynes [29,30,31,32,33,34,35,36,37]. The so-obtained conjugates exhibited high antiproliferative activity and appeared to be less toxic, more selective against normal cells [31,33,36], and possessed antiprotozoal [34,35] and antifungal potency [37]. The activity was strongly dependent on the nature of substituents at the 4 position of the triazole ring. Based on these results, we have synthesized quinine–triazole hybrids carrying an aminopropargylmethoxymethyl substituent at the C-4 position of the triazole unit (Figure 1E,F). Propargylamines have shown wide application in medicinal and pharmaceutical chemistry and have gained some importance in the area of antibacterial and anticancer research [38,39]. A three-component copper-catalyzed coupling reaction among alkyne (terminal acetylene), aldehyde, and amine (A^3^-coupling) represents a sustainable and straightforward synthetic approach for the synthesis of these derivatives [40,41].

Therefore, we prepared some quinine conjugates by linking 9-azido-(9-deoxy)quinine with terminal alkynes, including 2-methylbut-3-yn-2-ol. The reaction of (9-(4-oxymethyl)-1,2,3-triazolyl)-*epi*-quinine with propargyl bromide in DMF in the presence of NaH led to the formation of a mixture of (9*R*)- and (9*S*)-((4-(prop-2-ynyloxy)methyl)triazolyl derivatives of cinchona alkaloids. The newly synthesized 9-(((4-X-but-2-ynyloxy)methyl)-1,2,3-triazolyl)-9-desoxy-cinchona alkaloid derivatives were found to exhibit antibacterial activity against *Staphylococcus aureus*, *Bacillus cereus*, and *Escherichia coli* strains. The disruption of biofilm formation for the new cinchona alkaloid derivatives was also studied. For the most active compounds, the study of interactions with the targeted protein MurB was performed.

## 2. Results and Discussion

**Scheme 1:**
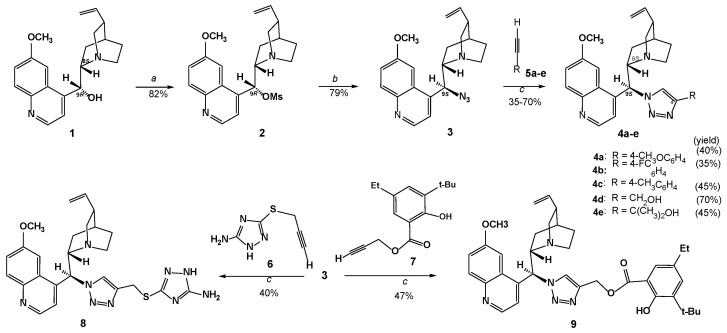
Synthesis of 9-[(4-X-1,2,3-triazol-1-yl)-substituted *epi*-quinine derivatives **4a**–**e**, **8**, and **9** from (-)-quinine **1**. Reagents and conditions: (*a*) MsCl, TEA, toluene, 0 → 20 °C, 24 h; (*b*) NaN_3_, DMF, 40–50 °C, 12h; (*c*) CuSO_4_ × 5H_2_O, sodium ascorbate, DMF, 70–75 °C, 6–8 h.

The strategy for the synthesis of 9-(((4-N-but-2-ynyloxy)methyl)-1,2,3-triazolyl)-substituted 9-deoxy-cinchona alkaloid conjugates was initiated with the synthesis of the azido-derivative of quinine **1**. The free hydroxy group at C-9 of quinine **1** was chosen to be converted into an azide group. Simple mesylation of the hydroxy group through treatment with methanesulphonyl chloride in the presence of triethylamine afforded *O*-mesylated quinine derivative **2** [42], which was, as such, subjected to heating with sodium azide in DMF, which afforded 9-*epi*-9-azido-9-deoxyquinine **3** [43] (Scheme 1). An increase in the reaction temperature above 70 °C led to a decrease in the yield of the target product. The synthesis of 9-(4-substituted 1,2,3-triazole)-9-*epi*-9-desoxyquinine derivatives **4a**–**e** was achieved through the Cu-catalyzed azide–alkyne cycloaddition of 9-*epi*-9-azido-9-deoxyquinine **3** with aryl alkynes **5a**–**c**, propargyl alcohol **5d**, and 2,2,-dimethylpropargyl alcohol **5e**. An aqueous CuSO_4_ and sodium ascorbate mixture was taken as the catalyst system to generate Cu(I)-catalyst in situ. A 6–8 h reaction in DMF at 70–75 °C gave good yields of final products **4a**–**e** (isolated yield, 35–70%). The main decrease in the isolation yield to 35% was observed in the reaction of **3** with 4-fluorophenylacetylene (**5b**). The azide–alkyne cycloaddition reaction of 9-*epi*-9-azido-9-deoxyquinine **3** with substituted terminal alkynes **6** or **7** under the mentioned conditions afforded the target compounds **8** or **9** in a yield of 40–47%. The composition and structure of all synthesized compounds were confirmed by IR, ^1^H, and ^13^C NMR spectroscopy and mass spectrometry (HRESI-MS). The ^1^H and ^13^C NMR spectra of the synthesized compounds contained one set of characteristic signals for the quinine core and the corresponding substituent. The structure of compound **4e** was established by X-ray structure analysis data.

**Figure 2:**
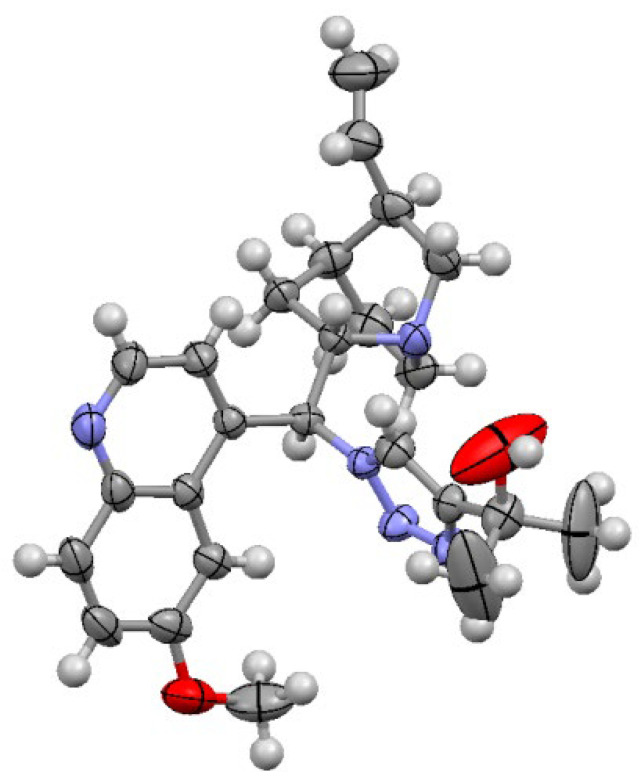
The molecular structure of2-(1-((*S*)-(6-methoxyquinolin-4-yl)((*2S*,*4S*,*5R*)-5-vinylquinuclidin-2-yl)methyl)-1*H*-1,2,3-triazol-4-yl)propan-2-ol (**4e**). The thermal ellipsoids are drawn at the 30% probability level (CCDC 2447839).

The molecular structure of compound **4e** is shown in Figure 2. The geometry of molecule **1** is very close to that of a similar compound, 6-methoxy-4-((4-methoxycarbonyl-1*H*-1,2,3-triazolyl)(5-ethenyl-1-azabicyclo [2.2.2]octan-2-yl)methyl)quinoline [30]. In the crystal, molecules **4e** are linked in chains along the *c* axis by O-H…N(quinoline) hydrogen bonds (H…N 2.05, O…N 2.837(7) Å, O-H…N 162°).

**Scheme 2:**
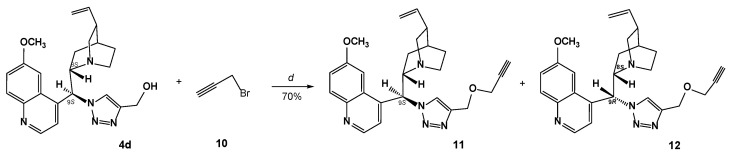
Synthesis of 2(*S*)- (**11**) and 2(*R*)- (**12**) 2-(6-methoxyquinolin-4-yl)(4-(2-(prop-2-ynyloxy)propan-2-yl)-1*H*-1,2,3-triazol-1-yl)methyl)-5-vinylquinuclidines. Reagents and conditions: (*d*) NaH (3 equiv.), DMF, 0 → 20 °C, 8 h.

Recently, C-9 derivatives of cinchona alkaloids have attracted considerable attention, as they have a functional group that introduces steric hindrance between quinuclidine and quinoline moieties and modifies polarity that is lower than in the original alkaloids. The specific functional substituent on the 4 position in the 1,2,3-triazole ring has different effects on pharmacological properties [33,35,37]. Therefore, in the next step, we studied the *O*-propargylation reaction of compound **4d**. When the reaction of **4d** with **10** was carried out in the presence of NaH (70% in mineral oil, 3 equiv.) in DMF under argon flow at RT for 8 h, two separable diastereomeric products, **11** (without change of configuration) and **12** (with inversion of configuration at C9), were isolated in a ca. 1:1.3 ratio in a total yield of 70% (Scheme 2). The more polar diastereomeric compound, **12**, was favored over the less polar **11**. The two easily separated compounds, **11** and **12**, formed in the reaction of propargyl bromide **10** with 8*S*,9*S*-quinine triazole **4d** in the presence of a strong base, revealed the same *m*/*z*, but the respective specific rotations for (8*S*,9*S*)-**11** ([α]_D_^24^ (−) 53.33 (c 0.6, CHCl_3_) and (8*S*,9*R*)-**12** ([α]_D_^24^ + 122.9 (*c* 0.95, CHCl_3_) differed. The ^1^H NMR spectra for the samples of **11** and **12** displayed inequalities in the chemical shifts of H-3′ (∆δ = 0.25 ppm), H-5 in the triazole ring (∆δ = 0.11 ppm), and H-7 on the quinuclidine moiety (approximately δ = 1.40 ppm for (+)-**12** and δ = 0.88 ppm for (−)-**11**). The chemical shifts of all other protons differed slightly (∆δ ≤ 0.03 ppm). Notably, the large values of coupling constant J (H8-H9) = 11.3 Hz confirmed the anti-orientation between the H-8 and H-9 protons and suggested that H-8 and H-9 are in antiperiplanar conformation (open conformation) in both compounds [44]. The greatest difference in the ^13^C NMR spectra of **11** and **12** is in the chemical shifts of the carbon atom signal C-7 (for (−)-11–δ 27.5 ppm and for (+)-12–25.1 ppm). It is obvious that, under the action of the strong base (NaH), inversion occurs at C-9, forming a mixture of easily separated diastereomeric alkaloids, **11** and **12**, in which the C-9-*nat* configuration predominated.

**Scheme 3:**
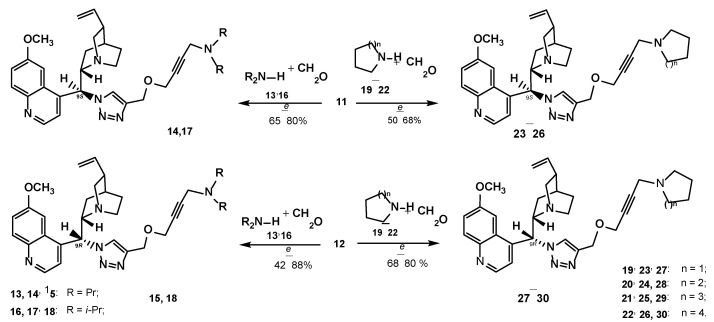
Synthesis of 9-((4-X-(but-2-ynyloxy)methyl)-1*H*-1,2,3-triazol-1-yl)-9-deoxy-cinchona alkaloid derivatives **14**, **15**, **17**, **18**, and **23**–**30**. Conditions: (*e*) Cu(OAc)_2_ × H_2_O, 1,4-dioxane, 60–70 °C, 8 h.

Our main focus was the introduction of a variety of nitrogen-substituted 9-((4-N-but-2-ynyloxymethyl)triazolyl)-moieties in 9-deoxy-cinchona alkaloids (Figure 1). For the synthesis of these compounds, we studied the copper-catalyzed A^3^-coupling reaction between 9*S*- and 9*R*-((4-prop-2-ynyloxymethyl)triazolyl)-9-deoxycinchona alkaloid derivatives **11** or **12**, aq. formaldehyde, and different secondary amines. Notably, a great number of transition-metal catalysts were exploited in the A^3^-coupling process, and the Cu(I)/Cu(II) salts remained the most extensively applied and studied [40,41]. We found that the reaction of compound **11** or **12**, aq. formaldehyde (4 equiv.), and dipropylamine **13** (1.2 equiv.) in the presence of copper(II) acetate monohydrate (10% mol) in 1,4-dioxane in an argon flow proceeds smoothly, and by heating it at 60–70 °C for 6 h, the alkyne was almost consumed; after the treatment of the reaction mixture and column chromatography, the desired 9-((4-(4-(dipropylamino)-but-2-ynyloxy)methyl)-1*H*-1,2,3-triazol-1-yl)-9-deoxycinchona alkaloid derivatives, **14** or **15**, were isolated in a yield of 80–88% (Scheme 3). The reaction was efficient and highly diastereoselective. In these conditions, compounds **11** and **12** were reacted with aq. formaldehyde and diisopropylamine **16** (65 °C, 6 h) to give the desired compounds, **17** and **18**, in high isolated yields.

Three-component reaction of cyclic secondary amines: Pyrrolidine **19**, piperidine **20**, azepane **21**, and azocane **22** with aq. formaldehyde and 9*S*-(propynyloxyprop-2-yl-triazolyl)-**11** or 9*R*-(propynyloxyprop-2-yl-triazolyl)-9-deoxycinchona alkaloid derivative **12** in the presence of Cu(OAc)_2_ × H_2_O in the above conditions afforded 9-((4-(4-(diisopropylamino)but-2-ynyloxy)methyl)-1*H*-1,2,3-triazol-1-yl)-substituted derivatives of cinchona alkaloids **23**–**26** or **27**–**30** in high yields. All the derivatives were purified by column chromatography (chloroform–ethanol, solvent mixture). Diastereomeric compounds **23** and **27** or **26** and **30** were obtained by introducing a mixture of **11** + **12** into the reaction and through the subsequent separation of the reaction mixture via column chromatography. It must be noted that the ratio of diastereomeric compounds in this transformation was very similar to that for compounds **11** and **12**.

^1^H-NMR, ^13^C NMR, and mass spectral analysis were found to prove the expected structures. We note that the vicinal coupling constant ^3^J_H8H9_ was 11.2 and 11.5 Hz for compounds **23**–**30**, and it belongs to two open conformers. The ^1^H and ^13^C NMR spectra corroborate the presence of an intact framework of quinine. The distinguishing features of the ^13^C NMR spectra of compounds **12**, **14**–**17**, and **22**–**26** are the broadening of the signal of the H-9 carbon atom. It is characteristic that in the spectra of 9-triazolyl-substituted derivatives of *epi*-quinines **4a**–**e**, **8**, and **9**, a broadening of this signal can also be observed.

**Figure 3:**
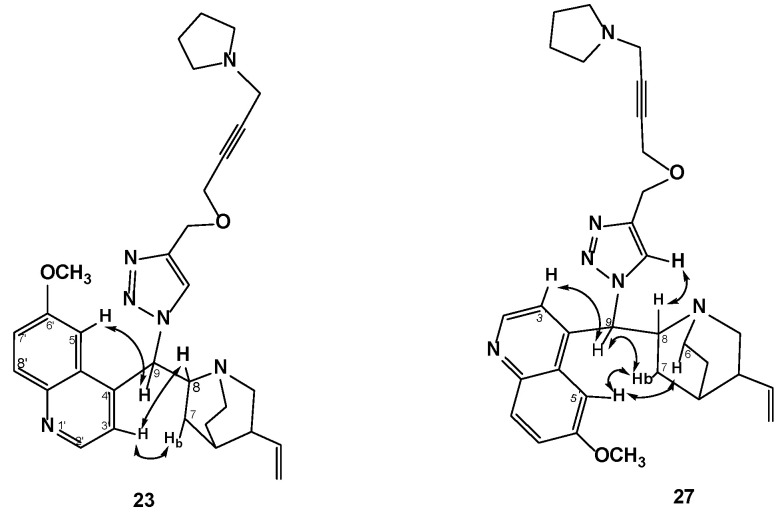
Conformational analysis of compounds **23** and **27** in a solution of CDCl_3_.

Theoretical considerations assume four energetically possible conformers of cinchona alkaloid molecules: two closed (*anti*- and *syn*-) and two open (*anti*- and *syn*-). There is a possibility of easy rotations in molecules around the C8-C9 bond (giving closed and open conformers), as well as around the C4′-C9 bond (giving *syn*- and *anti*-conformers) [44,45,46]. We note that the vicinal coupling constant ^3^J_H8H9_ was 11.2 and 11.5 Hz for compounds **23**–**30**, and it belongs to two open conformers. The configuration of diastereomeric compounds **23** and **27** was established with the help of nuclear Overhauser effect NMR experiments. Strong NOESY correlation of H-9 with H-6 and H-7, but only a faint cross peak with H-8, suggested that H-9 and H-8 are in an open conformation for both compounds. Correlation of the quinoline H-3′ and H-5′ atoms with H-8 of the quinuclidine and H-9, respectively, established the anti-orientation of the heterocyclic rings. The strong NOE between H-5′ (δ 7.51 ppm) and H-8 (δ 3.85 ppm) suggests the 23 structure of the open (A)conformer (Figure 3). The presence of the (A) conformer is confirmed by the NOE between H-3′ (δ 7.45 ppm) and H-7a (δ 1.61 ppm) (compound **23**). In the spectra of compound **27**, we observe strong NOE effects between H-9 (δ 6.39 ppm) and H-7b (δ 1.35 ppm), and also H-9 (δ 6.39 ppm) and H-6a (δ 2.87 ppm), which would be indicative of conformer open (B). Additionally, an NOE of appreciable intensity was observed between H-9 and H-3′ (δ 7.64 ppm) and also between H-9 and H-5 of the triazole ring (δ 7.30 ppm).

Direct inversion of the 9-C center can be achieved under a variety of conditions. These reactions presumably occur rapidly. Reactions causing epimerization at C9 of cinchona alkaloids and C-9-substituted derivatives were reported in [47,48,49,50]. Wang and coworkers reported nucleophilic substitution of 2-bromo-1,10-phenanthroline with quinine in DMSO in the presence of NaH at 80–90 °C, which afforded 9-phenanthroline–quinine ligands as two diastereomers: without a change in configuration and with inversion of configuration at C9 [50].

In the present study, we synthesized several derivatives of quinine with specific functional groups in order to understand how different functional groups serve different antibacterial actions.

### 2.1. Biological Study

#### Antibacterial Activity 

Compounds **4a**–**d** and **8** were screened for their in vitro antibacterial activity against bacterial strains *Staphylococcus aureus* 209 ATCC 6538-P, *Bacillus subtilis* ATCC 6633, and *Escherichia coli* ATCC 25922 by the disc diffusion method. The activities of the target compounds were expressed as the mean diameter of the measured inhibition zone (mm) against selected microorganisms, along with the activity of the reference compound Gentamicin (Table 1). All 9-triazolyl-*epi*-quinine derivatives suppress the growth of the *E. coli* strain (MIC = 12.5–50 µM). Compound **4b**, with the structure of 9-(4-fluorophenyltriazolyl)-*epi*-quinine, statistically significantly inhibited *S. aureus* growth. Only compound **4a** was active on the *B. subtilis* strain. In broth, microdilution assay compound **4b**, with 9-(4-fluorophenyltriazolyl)- and compound **8**, with a (5-amino-1*H*-1,2,4-triazolyl-methylthio)-substituent at the 4 position of 9-triazolyl-*epi*-quinine, suppressed *S. aureus* at a dose of 25 µM, which is two times lower than that of the reference compound gentamycin (MIC 11.8 µM). Thus, in the series of novel triazolyl derivatives of *epi*-quinine, compounds **4a** and **8** showed moderate activity against the *S. aureus* and *E. coli* strains.

Compounds **11**, **12**, **14**, **15**, **17**, **18**, and **23**–**30** and quinine **1** were screened for their in vitro antibacterial activity against two bacterial strains: *Staphylococcus aureus* 209 ATCC 6538-P and *Bacillus cereus* ATCC 10702. The antibacterial activity was studied by serial dilution in a liquid nutrient medium. The minimum inhibition concentrations (MICs) of the test cultures were determined. No bioactivity was defined as an MIC > 1000 µg/mL, mild bioactivity as an MIC in the range 512–1000 µg/mL, moderate bioactivity as an MIC in the range 128–512 µg/mL, and good bioactivity as an MIC in the range 32–128 µg/mL. The results are provided in Table 2. The parent substance quinine **1** does not have antibacterial properties under the specified conditions. According to [20], *S. aureus* was inhibited at an MIC of 125 g/mL of quinine hydrochloride. The difference in the antibacterial activity profile of diastereoisomeric 9-(((prop-2-ynyloxy)methyl)triazolyl)-1,2,3-triazolyl- **11**, **12** and the 9-(((4-X-but-2-ynyloxy)methyl)-1,2,3-triazolyl)-**14**, **15**, **17**, **18**, **23**–**30**-substituted derivatives of cinchona alkaloids indicates the importance of the nature of the nitrogen substituents in the molecules. Compounds with a linear dipropylamino-**14**, **15** or diisopropylamino-**17**, **18** substituents in the 4 position of the (but-2-ynyloxy)methyl)-1,2,3-triazolyl moiety were less active. The heterocyclic substituent in the 9-((4-X-but-2-ynynoxy)methyl)-1,2,3-triazolyl)-moiety (derivatives **23**–**30**) enhances activity. Derivatives **23**–**28** displayed moderate activity toward *S. aureus* and *B.cereus* with MIC values of 416–200 and 283–545 µg/mL. The configuration of the C8/9 position is also important for antibacterial activity, and the (9*R*) configuration is the dominant configuration for *S. aureus* treatment (compounds **27**–**29**). A similar difference was observed in the antibacterial activity of the (9*R*)- and (9*S*)-diastereomers in a series of N-substituted quaternary salts made of cinchonidine and cinchonine compounds [51]. Characteristically, with an increase in the volume of the heterocyclic substituent (X) in the molecule, differences in the activities of diastereomers are leveled (cf. compounds **26** and **30**). Concerning the structure–activity relationships for both test cultures used, the highest activity was found for compounds **26** and **30** on both bacterial strains. However, these new derivatives, while showing improved activity relative to quinine itself, are still substantially less potent than the clinical antibiotic ceftriaxonum (Table 2).

*S. aureus* is a widespread pathogen affecting both human and veterinary medical practice. *S. aureus* is one of the leading bacteria that causes biofilm-associated infections related to medical devices [52]. Approximately 80% of nosocomial infections caused by *S. aureus* are associated with the ability of these microorganisms to form biofilms. Bacterial biofilm strongly promotes the development of pathogenic drug resistance in clinical treatments. *S. aureus* biofilms increase resistance to chemotherapeutic agents, leading to increased severity, recurrence, chronicity, and mortality from the infections they cause [53]. For optimization, the effectiveness of infectious disease treatment, except for antibiotic dose reduction, is significant in biofilm disruption. Synthesis and studies of potent antibiofilm agents have considered this topic [54]. Taking into account the biofilm formation-blocking properties of several cinchona alkaloid derivatives [55], we studied the effect of diastereomeric compounds **26** and **30** on biofilm formation.

The ability of new quinine derivatives to inhibit biofilm formation was studied using the method described in [56]. By introducing *S. aureus* culture at a dose of (6.09 ± 0.31) × 10^3^ CFU, we found that substance **26** completely suppressed bacterial growth in concentrations of 181 ± 12.8 µg/mL, and compound **30** possessed an MIC value equal to 179.2 ± 18.4 µg/mL (Table 2). Suppression of film formation for substance **26** had an MIC of 107.4 ± 7.7 µg/mL, while compound **30** showed a value of 82.4 ± 8.7 µg/mL. These data showed the good antibacterial activity of diastereomeric compounds **26** and **30** on *S. aureus* cultures.

**Figure 4:**
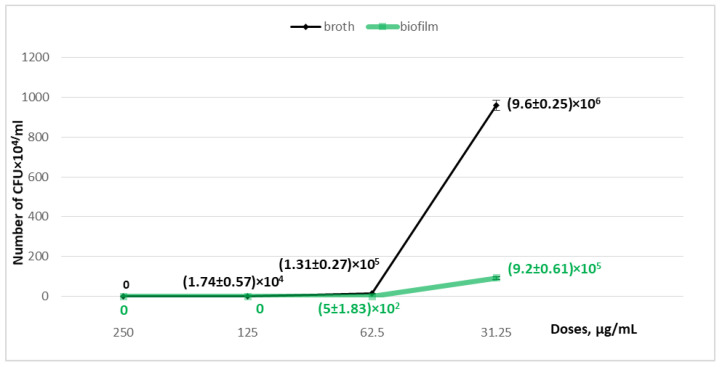
The effect of conpound **26** on inhibition of *S. aureus* growth and film formation in a liquid medium.

Interesting results regarding the film formation dynamics were obtained for both diastereomeric compounds. A dose-dependent growth inhibition effect was observed for compound **26**. At a dose of 125 µg/mL, a decrease in bacterial growth to (1.74 ± 0.57) × 10^4^ CFU/mL was noted–as well as at a dose of 62.5 µg/mL–to (1.31 ± 0.27) × 10^5^ CFU/mL and at a dose of 31.25 µg/mL to (9.6 ± 0.25) × 10^6^ CFU/mL–against (1.38 ± 0.12) × 10^7^ CFU/mL in the control sample, without the addition of the substance. The dynamics of the substance’s effect on film formation were studied in the same concentrations. At a dose of 125 µg/mL, film formation was completely suppressed; at a dose of 62.5 µg/mL, the number of bacteria was (5.0 ± 1.83) × 10^2^ CFU/mL; at a dose of 31.25 µg/mL, it was (9.2 ± 0.61) × 10^5^ CFU/mL, with the presence of bacterial attachment in the control sample in an amount equal to (2.9 ± 0.2) × 10^6^ CFU/mL (Table 3, Figure 4).

**Figure 5:**
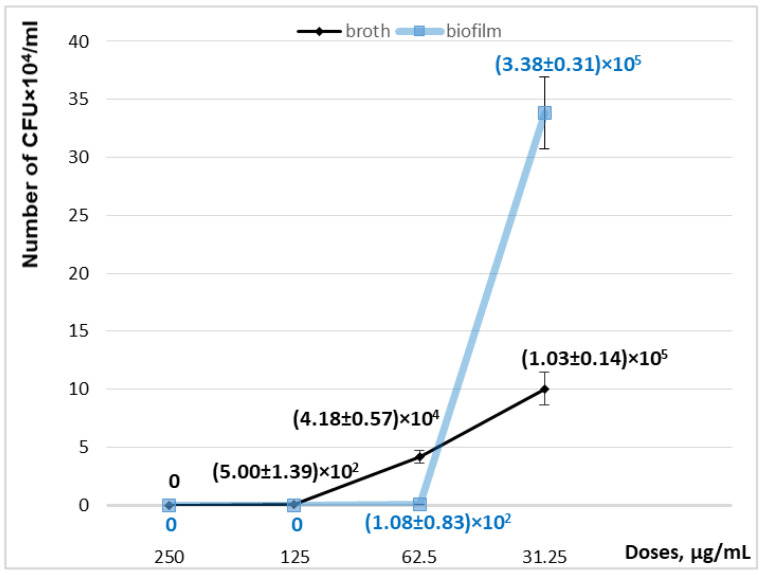
The effect of compound **30** on inhibition of *S. aureus* growth and film formation in a liquid medium.

For substance **30**, the dynamics of the decrease in culture growth were as follows: for a dose of 125 µg/mL, (5.0 ± 1.39) × 10^2^ CFU/mL; for 62.5 µg/mL, (4.18 ± 0.57) × 10^4^ CFU/mL; and for 31.25 µg/mL, (1.03 ± 0.14) × 10^5^ CFU/mL against (1.38 ± 0.12) × 10^7^ CFU/mL in the control sample without the addition of the substance. As with the diastereomeric compound **30**, a dose of 125 µg/mL completely inhibited the adhesive activity of *S. aureus*, a concentration of 62.5 µg/mL reduced the number of attached bacteria to (1.08 ± 0.83) × 10^2^ CFU/mL, and a does of 31.25 µg/mL reduced it to (3.38 ± 0.31) × 10^5^ CFU/mL (Table 4, Figure 5). The amount of bacterial attachment in the control sample was (2.9 ± 0.2) × 10^6^ CFU/mL.

The obtained results demonstrated for the first time the ability of new cinchona alkaloid derivatives to suppress the growth of *S. aureus* and its adhesive activity, which demonstrates the prospects for further study of (9*R*)- and (9*S*)-diastereomeric compounds **26** and **30** as potential antibacterial agents. Notably, according to [21], quinine sulfate inhibits the invasion of some bacterial pathogens at a dose above 100 mg/mL.

### 2.2. Docking Studies of Compounds ***1***, ***4d***, ***11***, ***12***, ***23***, ***26***, ***27***, and ***30*** in the FAD Site of MurB

MurB (UDP-N-acetylenolpyruvylglucosamine reductase) plays a critical role in bacterial peptidoglycan biosynthesis, catalyzing the NADPH-dependent reduction of UDP-N-acetylenolpyruvylglucosamine (UDP-EP-GlcNAc) to UDP-N-acetylmuramic acid (UDP-MurNAc), a precursor for cell wall assembly [57]. This enzyme utilizes FAD (flavin adenine dinucleotide) as a non-covalently bound prosthetic group to facilitate hydride transfer: NADPH reduces FAD to FADH2, which then reduces the enolpyruvyl substrate [58]. As MurB is essential for bacterial viability and lacks eukaryotic homologs, it represents an attractive target for selective antibacterial agents [59]. Docking into the FAD binding site is justified as a strategy for antibacterial drug development, as competitive or allosteric inhibitors can disrupt cofactor association or catalytic function, halting peptidoglycan synthesis [60,61]. Previous studies have validated this approach, with 3,5-dioxopyrazolidines binding adjacent to FAD in MurB and inhibiting activity [60], and virtual screening identifying FAD-competitive leads against *Mycobacterium tuberculosis* MurB [62]. The quinine derivatives examined here, incorporating triazole and alkyne motifs known for antibacterial enhancement in quinoline and coumarin scaffolds [63,64], align with this paradigm by occupying FAD sub-regions and forming analogous interactions.

We have performed molecular docking simulations to evaluate the binding affinities and interaction profiles of quinine (compound **1**), 9-((4-hydroxymethyl)-1,2,3-triazol-1-yl)-*epi*-quinine **4d**, 9-(((4-prop-2-ynyloxy)methyl)- 1,2,3-triazol-1-yl)- **11**, **12**, 9-((((4-pyrrolidin-1-yl)but-2-ynyloxy)methyl)-1,2,3-triazol- 1-yl)- **23**, **27** and 9-((((4-asocan-1-yl)but-2-ynyloxy)-methyl)-1,2,3-triazol-1-yl)- **26**, **30** containing derivatives within the FAD binding site of MurB from *Staphylococcus aureus* (PDB: 1HSK).

The docking scores (DSs), expressed in units of the MolDock force field [65], ranged from −123.5 for compound **1** to −226.4 for compound **30**, indicating significant binding affinities depending on structural modifications (Table 5). For reference, the co-crystallized FAD exhibited a substantially lower DS of −315.5, reflecting its native high-affinity interaction as the prosthetic group of MurB. Key interacting residues were identified based on partial MolDock scores below −10 units, with hydrogen bonding patterns. Ligand moiety placements relative to FAD fragments were also characterized (Table 5).

**Figure 6:**
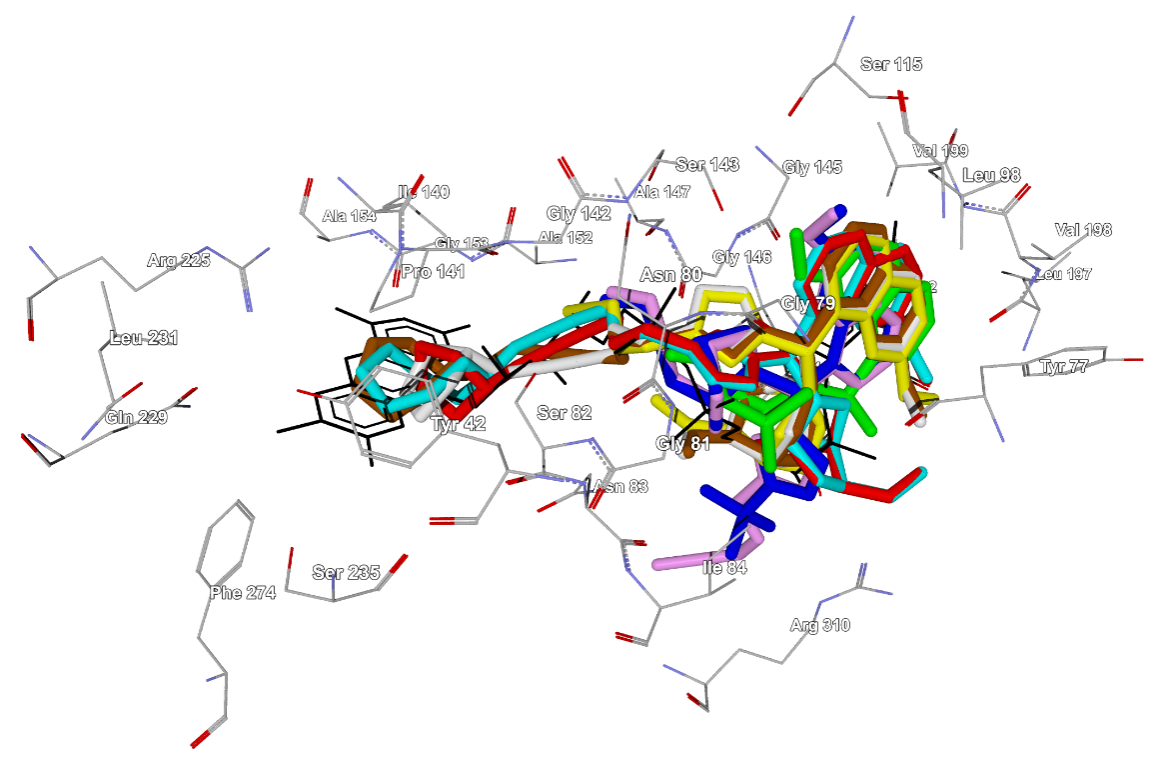
The superimposed docking poses of compounds **1** (green), **4d** (blue), **11** (magenta), **12** (yellow), **23** (red), **27** (gray), **26** (light blue), and **30** (brown). The co-crystallized FAD molecule is shown in thin black sticks. The residues within 3 Å of FAD are visible.

The binding modes revealed consistent occupancy of the FAD adenine region by the quinoline moiety across all compounds, suggesting mimicry of the adenine base (Figure 6). For compound **1** (quinine), the quinuclidine group aligned with the FAD ribose, and the vinyl substituent overlapped the pyrophosphate. Molecule **1** in its docking pose forms hydrogen bonds with Tyr77, Ser143, and Val199 residues. Introduction of a triazole ring in compound **4d** shifted the quinuclidine and vinyl toward the pyrophosphate, with the triazole on ribose yielding hydrogen bonds to Ser143, Tyr149, and Arg310. Addition of the propargyloxy group to compounds **11** and **12** further refined the poses: in **11**, propargyloxy extended from FAD, while in **12**, it aligned with the ribitol fragment; the hydrogen bonds involved Ser143, Tyr149, Arg310, and Ser82 (propargyloxy in **12**). Compounds **23**, **26**, **27**, and **30**, bearing heterocyclic substituents at the terminal alkyne (pyrrolidine-containing in **23** and **27**; azocane-containing in **26** and **30**), displayed the most favorable DS values, with the additional heterocycles positioned near the FAD isoalloxazine ring. Hydrogen bonding patterns emphasized interactions with Gly79, Gly81, Ser143 (triazole), and Ser82 (propargyloxy), alongside van der Waals contacts with Tyr77, Tyr149, Leu78, and Gly146. Common residues across the series included Tyr77, Tyr149, Leu78, Gly145, Gly146, Ser143, and Asn83, which partially overlap with FAD’s interactions (e.g., Tyr77, Tyr149, Ser82, Ser143, Asn83, Gly79, Gly145, and Gly146). Several residues identified in this study have been reported to play a role in the binding of coumarin-based alkynes to MurB [64]. However, ligands **1**, **4d**, **11**, **12**, **23**, **26**, **27**, and **30** showed no significant interaction with FAD-specific residues such as Val199 (except for quinine) and Arg225, which is consistent with their smaller size and preferential binding to sub-pockets.

The binding modes demonstrate a progression from a basic quinine scaffold to more elaborate derivatives that better exploit the FAD pocket. The quinoline consistently mimics adenine, while triazole and propargyloxy moieties enable phosphate/ribitol mimicry, and terminal heterocycles extend toward isoalloxazine (Figure 6), correlating with DS improvements (Table 5). The hydrogen bonding pattern shifts from methoxy/hydroxyl-focused interactions in simpler compounds to triazole- and propargyloxy-centered networks in advanced ligands. Key residues—Ser143, Ser82, Gly79, and Gly81—frequently act as hydrogen bonding centers, mimicking the interaction pattern of FAD but forming fewer hydrogen bonds, which may explain the observed DS gap.

Comparative analysis of diastereomeric pairs (**11**/**12**, **23**/**27**, and **26**/**30**) reveals stereochemical influences: the (9*R*) configuration at the stereodivergent center (compounds **12**, **27**, **30**) consistently yielded more negative DSs (−191.0, −216.3, and −226.4) than the (9*S*) configuration for **11**, **23**, and **26** (−185.7, −199.2, and −207.7), suggesting better steric accommodation in the chiral FAD pocket [58]. Interaction profiles were similar, with shared residues (e.g., Tyr149 and Leu78) and hydrogen bonds (e.g., Ser82-propargyloxy), but (9*R*)-isomers often engaged additional contacts (e.g., Ser143-triazole in **12**, **27**, and **30**) or a weaker Arg310 bond in (9*S*)-isomer **11** (Table 5).

## 3. Materials and Methods

### 3.1. Chemistry

#### 3.1.1. General Information

Melting points were determined on the Mettler Toledo FP900 thermosystem (Columbus, OH, USA). NMR spectra were acquired on Bruker Avance 300 (^1^H: 300.13 MHz, ^13^C: 75.48 MHz), Bruker Avance-400 (^1^H: 400.13 MHz, ^13^C: 100.78 MHz), or Bruker DRX 500 (^1^H: 500.13 MHz, ^13^C: 125.76 MHz) spectrometers (Bruker Corporation, Karlsruhe, Germany). Deuterochloroform (CDCl_3_ or (CD_3_)_2_SO for compound **8**) was used as a solvent. By using CDCl_3_ as a solvent, the residual CHCl_3_ (δ_H_ = 7.24 ppm) and CDCl_3_ (δ_C_ = 76.8 ppm) could be employed as internal standards, and by using (CD_3_)_2_SO as a solvent the residual of DMSO (δ_H_ = 2.51 ppm) and CD_3_)_2_SO (δ_C_ = 39.51 ppm) was employed as the internal standard. NMR signal assignments were carried out with the aid of a combination of 1D and 2D NMR techniques that included ^1^H and ^13^C spectra. The ^13^C NMR signal multiplicity was determined using the standard methods of recording spectra in *J*-modulation resonance mode. For diastereomers **23** and **27**, additionally, COSY, NOESY (with 1 s mixing time and 2 s delay between pulses), HSQC, and HMBC spectra were recorded. In the description of the ^1^H and ^13^C-NMR spectra for all compounds, the chinchone alkaloid skeleton atom numeration system provided in structure **F** was used (Figure 1).

The NMR (^1^H and ^13^C) spectra of **4a**–**e**, **8**, **9**, **11**, **12**, **14**, **15**, **17**, **18**, and **23**–**30** are provided in the Supplementary Materials. IR spectra were recorded by means of the KBr pellet technique on an Avatar 360 ESP spectrometer (Thermo Nicolet, Madison, WI, USA). Mass spectra were recorded (Thermo Fisher Scientific, Waltham, MA, USA) in full-scan mode (15–500 *m*/*z*; evaporator temperature, 210–280 °C; 70 eV electron-impact ionization). The specific rotation values, [α]_D_, were determined on a PolAAr 3005 polarimeter (Optical Activity Ltd., Huntingdon, UK). An X-ray structural study of compound **4e** was performed on a Bruker KAPPA APEX II diffractometer with a two-dimensional CCD detector (MoKα radiation with a graphite monochromator and ω-φ-scanning).

The reaction progress was controlled by TLC on Sorbfil UV-254 plates using CHCl3- EtOH mixtures (100:1 for **4a**–**e**, 20:1 or 10:1 for **8**, **9**, **11**, **12**, **14**, **15**, **17**, **18**, and **23**–**30**) as eluents, with visualization under UV light. Products were isolated by column chromatography on silica gel Acros (0.063–0.200 mm or 60–0.120 mm, Merck KGaA, Darmstadt, Germany), eluting with indicated solvent systems. The chemicals used included the following: sodium azide, MsCl, formalin (30% formaldehyde in aq. solution), sodium ascorbate, NaH, Cu(OAc)_2_ × H_2_O, CuSO_4_ × 5H_2_O, (-)-quinine **1**, propargyl bromide **10**, arylalkynes **5a**–**c**, propargyl alcohol **4d**, 2-methylbut-3-yn-2-ol **4e**, dipropylamine **13**, diisopropylamine **16**, pyrrolidine **19**, piperidine **20**, azepane **21,** and azocane **22**. These were purchased from Aldrich, Alfa Aesar, and Macklin. *O*-Mesylated quinine derivative **2** [42] and 9-*epi*-9-azido-9-deoxyquinine **3** [43] were prepared following procedures in the literature. The solvents (DMF, 1,4-dioxane, CHCl_3_, EtOH) and Et_3_N were purified according to standard methods and distilled immediately before use.

#### 3.1.2. Experimental Procedure

##### Preparation of Compounds **4a**–**e**,**8**,**9**

A mixture of 0.54 g (1.54 mmol) of azide **3**, alkynes **5a**–**e** (1.5 mmol), CuSO_4_ × 5H_2_O (17 mg, 0.07 mmol), and sodium ascorbate (14 mg, 0.07 mmol) in DMF (5 mL) was stirred for 6–8 h at 70–75 °C (TLC-control). The mixture was evaporated under reduced pressure; the residue was diluted in CHCl_3_ and subjected to column chromatography (eluent: CHCl_3_ → CHCl_3_-EtOH, 100:1) to provide compounds **4a**–**e.**

*(2R*,*4S*,*5S)-2-((S)-(4-(4-Methoxyphenyl)-1H-1*,*2*,*3-triazol-1-yl)(6-methoxyquinolin-4-yl)methyl)-5-vinylquinuclidine* (**4a**). Yellowish solid; yield 40% (0.27 g); mp 71–75 °C (decomp.); [α]_D_^26^-178.3 (*c* 0.5, CHCl_3_). IR (KBr) ν: 1620, 1591, 1508, 765, 712 (C=C), 1065, 1032 (-C-O-C) cm^−1^; ^1^H NMR (CDCl_3_, 300 MHz) δ: 0.92–0.96 (1H, m, H-7), 1.56–1.72 (2H, m, H-5), 1.77–1.81 (1H, m, H-4), 1.90–1.98 (1H, m, H-7), 2.31–2.36 (1H, m, H-3), 2.66–2.84 (2H, m, H-2,6), 3.21 (1H, dd, J = 13.5, 10 Hz, H-2), 3.48–3.54 (1H, m, H-6), 3.76 (3H, s, OCH_3_-Ar), 3.94 (3H, s, OCH_3_-C-6′), 3.92–3.97 (1H, m, H-8), 5.06 (1H, dd, J = 1.8, 10.3 Hz, H-11), 5.07 (1H, dd, J = 1.8, 17.2 Hz, H-11), 5.91 (1H, ddd, ^3^J = 17.2, 10.3, 7.5 Hz, H-10), 6.47 (1H, d, J = 11 Hz, H-9), 6.84 (2H, d, J = 8.85 Hz, H-3,5-Ar), 7.35 (1H, dd, J = 9.3, 2.5 Hz, H-7′), 7.51 (1H, d, J = 4.55 Hz, H-3′), 7.54 (1H, d, J = 2.5, H-5′), 7.55 (1H, s, H-triazole), 7.63 (2H, d, J = 8.85 Hz, H-2,6 Ar), 8.00 (1H, d, J = 9.3 Hz, H-8′), 8.82 (1H, d, J = 4.5 Hz, H-2′) ppm; ^13^C NMR (CDCl_3_, 75 MHz) δ: 27.4 (C-5,7), 27.5 (C-4), 38.9 (C-3), 41.0 (C-6), 55.8, 55.1 (2× OCH_3_), 55.9 (C-2), 57.8 (C-8), 60.7 (C-9), 100.7 (C-5′), 113.9 (C-3,5-Ar), 114.7 (C-11), 117.2 (C-3′), 119.2 (CH-triazole), 122.4 (C-7′), 123.2 (C-1 Ar), 126.8 (C-2,6-Ar), 128.1 (C-10′), 132.1 (C-8′), 139.0, (C-4′), 141.1 (C-10), 145.0 (C-9′), 147.2 (C-2′), 147.5 (C-triazole), 158.6 (C-6′), 159.3 (C-4 Ar) ppm; HR-MS, *m*/*z* (I*_rel._*, %): 481 ([M]^+^, 0.8), 306 (12), 186 (7), 173 (6), 136 (100), 81 (9); calcd for C_29_H_31_N_5_O_2_: 481.2472; found [M]^+^ *m*/*z*: 481.2476.

*(2R*,*4S*,*5S)-2-((S)-(4-(4-Fluorophenyl)-1H-1*,*2*,*3-triazol-1-yl)(6-methoxyquinolin-4-yl)methyl)-5-vinylquinuclidine* (**4b**). Colorless solid; yield 35% (0.23 g); mp 91–93 °C (decomp.); [α]_D_^26^-184.5 (*c* 0.7, CHCl_3_); IR (KBr, ν_max_): 3074, 1620, 1593, 1508, 759, 713 (C=C), 1230, 840 (C-F), 1043, 1032 (C-O-C) cm^−1^; ^1^H NMR (CDCl_3_, 400 MHz) δ: 0.94–0.98 (1H, m, H-7), 1.60–1.66 (2H, m, H-5), 1.78–1.82 (1H, m, H-4), 1.88–1.98 (1H, m, H-7), 2.36 (1H, m, H-3), 2.74–2.85 (2H, m, H-2,6), 3.23 (1H, dd, J = 13.5, 10 Hz, H-2), 3.52–3.56 (1H, m, H-6), 3.95 (3H, s, OCH_3_-C-6′), 3.98–4.06 (1H, m, H-8), 5.06 (1H, dd, J = 1.8, 10.4 Hz, H-11), 5.08 (1H, dd, J = 1.8, 17.2 Hz, H-11), 5.90 (1H, ddd, J = 17.2, 10.4, 7.5 Hz, H-10), 6.52 (1H, d, J = 11.0 Hz, H-9), 7.01 (2H, m, H-3,5-Ar), 7.36 (1H, dd, J = 9.2, 2.5 Hz, H-7′), 7.52 (1H, d, J = 4.6 Hz, H-3′), 7.56 (1H, d, J = 2.5, H-5′), 7.63 (1H, s, H-triazole), 7.68 (2H, m, H-2,6 Ar), 8.01 (1H, d, J = 9.2 Hz, H-8′), 8.83 (1H, d, J = 4.6 Hz, H-2′) ppm; ^13^C NMR (75 MHz, CDCl_3_) δ: 26.9 (C-5,7), 27.1 (C-4), 38.3 (C-3), 40.7 (C-6), 55.5 (OCH_3_), 55.6 (C-2), 57.6 (C-8), 60.3 (C-9), 100.3 (C-5′), 114.6 (C-11), 115.5 (d, J_C-F_ = 21.8 Hz, C-3,5 Ar), 118.0 (C-3′), 119.2 (CH triazole), 122.5 (C-7′), 126.3 (d, J_C-F_ = 3.3 Hz, C-1 Ar), 127.3 (d, J_C-F_ = 7.8 Hz, C-2,6, Ar), 128.1 (C-10′), 131.9 (C-8′), 138.7 (C-4′), 140.8 (C-10), 145.0 (C-9′), 146.8 (C-triazole), 147.2 (C-2′), 158.6 (C-6′), 162.1 (d, J_C-F_ = 247.23 Hz, C-4 Ar) ppm; HR-MS, *m*/*z* (I*_rel._*, %): 469 ([M]^+^, 0.5), 440 (1.5), 283 (3), 198 (3), 172 (2), 136 (100), 81 (9); calcd for C_28_H_28_FN_5_O: 469.2272; found [M]^+^ *m*/*z*: 469.2274.

*(2R*,*4S*,*5S)-2-((S)-(6-methoxyquinolin-4-yl)(4-p-tolyl-1H-1*,*2*,*3-triazol-1-yl)methyl)-5-vinylquinuclidine* (**4c**). Colorless solid; yield 45% (0.30 g); mp 95–97 °C (decomp.); [α]_D_^26^-157.1 (*c* 0.6, CHCl_3_). IR (KBr) ν: 3076, 3024, 1620, 1593, 1508, 832, 759, 715 (C=C), 1081, 1043, 1032 (C-O-C) cm^−1^; ^1^H NMR (CDCl_3_, 400 MHz) δ: 0.88–0.95 (1H, m, H-7), 1.51–1.66 (2H, m, H-5), 1.75–1.81 (1H, m, H-4), 1.90–1.99 (1H, m, H-7), 2.29 (3H, s, CH_3_-Ar), 2.31–2.38 (1H, m, H-3), 2.66–2.84 (2H, m, H-2,6), 3.20 (1H, dd, J = 13.5, 10 Hz, H-2), 3.42–3.51 (1H, m, H-6), 3.93 (3H, s, OCH_3_-C-6′), 3.90–3.97 (1H, m, H-8), 5.06 (1H, dd, J = 1.8, 17.2 Hz, H-11),5.08 (1H, dd, J = 1.8, 10.2 Hz, H-11), 5.91 (1H, ddd, J = 17.2, 10.2, 7.6 Hz, H-10), 6.59 (1H, d, J = 11 Hz, H-9); 7.12 (2H, d, J = 8.1 Hz, H-3,5-Ar), 7.35 (1H, dd, J = 9.3, 2.5 Hz, H-7′), 7.51 (1H, d, J = 4.6 Hz, H-3′), 7.55 (1H, d, J = 2.5, H-5′), 7.57 (1H, s, H-triazole), 7.59 (2H, d, J = 8.1 Hz, H-2,6 Ar), 8.00 (1H, d, J = 9.3 Hz, H-8′), 8.81 (1H, d, J = 4.6 Hz, H-2′) ppm; ^13^C NMR (CDCl_3_, 75 MHz) δ: 21.1 (CH_3_), 27.6 (C-5,7,4), 39.1 (C-3), 40.9 (C-6), 55.7 (OCH_3_), 56.0 (C-2), 57.8 (C-8), 60.8 (C-9), 100.7 (C-5′), 114.6 (C-11), 117.5 (C-3′), 119.1 (CH-triazole), 122.4 (C-7′), 125.4 (C-3,5 Ar), 127.6 (C-1 Ar), 128.2 (C-10′), 129.2 (C-2,6 Ar), 131.9 (C-8′), 137.6 (C-4 Ar), 139.0 (C-4′), 141.4 (C-10), 144.9 (C-9′), 147.2 (C-2′), 147.7 (C-triazole), 158.6 (C-6′) ppm; HR-MS, *m*/*z* (I*_rel._*, %): 466 (0.7), 465 ([M]^+^, 4), 307 (6), 279(6),184 (5), 175 (5), 155 (5), 137 (10), 136 (100), 81(7), 55 (5); calcd for C_29_H_31_N_5_O: 465.2523; found [M]^+^ *m*/*z*: 465.2518.

*(1-((S)-(6-methoxyquinolin-4-yl)((2R*,*4S*,*5R)-5-vinylquinuclidin-2-yl)methyl)-1H-1*,*2*,*3-triazol-4-yl)methanol* (**4d**). Colorless solid; yield 70% (0.57 g); mp 223–227 °C (decomp.); [α]_D_^26^-61.0 (*c* 0.5, CHCl_3_); lit.: mp 229–232 °C [30]; IR (KBr) ν: 3415 (OH), 3076, 3024, 1622, 1593, 1510, 833, 802, 759, 715 (C=C), 1041, 1033 (C-O-C) cm^−1^; ^1^H NMR (CDCl_3_, 500 MHz) δ: 0.82–0.94 (1H, m, H-7), 1.55–1.63 (2H, m, H-5), 1.78–1.82 (1H, m, H-4), 1.90–1.96 (1H, m, H-7), 2.25–2.38 (1H, m, H-3), 2.66–2.81 (2H, m, H-2,6), 3.13 (1H, dd, J = 13.5, 10.5 Hz, H-2), 3.35–3.42 (1H, m, H-6), 3.82–3.93 (1H, m, H-8), 3.93 (3H, s, OCH_3_-C-6′), 4.61 (2H, ABq, J = 12.6 Hz, ∆ν = 12.8 Hz, CH_2_O), 5.06 (1H, dd, J = 1.8, 17.2 Hz, H-11), 5.08 (1H, dd, J = 1.8, 10.2 Hz, H-11), 5.89 (1H, ddd, J = 17.2, 10.2, 7.5 Hz, H-10), 6.38 (1H, d, J = 11.2 Hz, H-9), 7.33 (1H, dd, J = 8.6, 2.7 Hz, H-7′), 7.44 (1H, s, H-triazole), 7.46 (1H, d, J = 4.6 Hz, H-3′), 7.50 (1H, d, J = 2.7, H-5′), 8.01 (1H, d, J = 8.6 Hz, H-8′), 8.76 (1H, d, J = 4.6 Hz, H-2′), (OH proton not observed) ppm; ^13^C NMR (100 MHz, CDCl_3_), δ: 27.3, 27.4 (C-5,7,4), 38.9 (C-3), 40.8 (C-6), 55.6 (OCH_3_), 55.8 (C-2), 56.5 (CH_2_O), 57.7 (C-8), 60.4 (C-9), 100.6 (C-5′), 114.8 (C-11), 119.1 (C-3′), 120.2 (CH-triazole), 122.3 (C-7′), 128.1 (C-10′), 131.8 (C-8′), 138.9 (C-4′), 141.1 (C-10), 144.8 C-9′), 147.2 (C-2′), 147.8 (C-triazole), 158.5 (C-6′) ppm.

*2-(1-((S)-(6-Methoxyquinolin-4-yl)((2S*,*4S*,*5R)-5-vinylquinuclidin-2-yl)methyl)-1H-1*,*2*,*3-triazol-4-yl)propan-2-ol* (**4e**). Colorless solid; yield 45% (0.28 g); mp 206–208 °C (decomp.); [α]_D_^26^-97.2 (*c* 0.5, CHCl_3_) white solid, m. p. 206–208 °C (decomp.); IR (KBr) ν: 3275 (OH), 3074, 1622, 1594, 1510, 835, 806, 798, 755, 714 (C=C), 1043, 1030 (C-O-C) cm^−1^; ^1^H NMR (CDCl_3_, 500 MHz) δ: 0.88–0.96 (1H, m, H-7), 1.51 (3H, s, CH_3_), 1.54 (3H, s, CH_3_), 1.59–1.67 (2H, m, H-5), 1.78–1.82 (1H, m, H-4), 1.84–1.96 (1H, m, H-7), 2.35–2.41 (1H, m, H-3), 2.76–2.89 (2H, m, H-2,6), 3.25 (1H, dd, J = 13.5, 10.2 Hz, H-2), 3.48–3.56 (1H, m, H-6), 3.94 (3H, s, OCH_3_-C-6′), 4.05–4.15 (1H, m, H-8), 5.07 (1H, dd, J = 2.0, 16.8 Hz, H-11), 5.08 (1H, dd, J = 2.0, 12.2 Hz, H-11), 5.89 (1H, ddd, J = 16.8, 12.2, 7.8 Hz, H-10), 6.46 (1H, d, J = 11.0 Hz, H-9), 7.35 (1H, dd, J = 9.2, 2.4 Hz, H-7′), 7.48 (1H, s, H-triazole), 7.50 (1H, d, J = 4.6 Hz, H-3′), 7.52 (1H, d, J = 2.4, H-5′), 8.00 (1H, d, J = 9.2 Hz, H-8′), 8.77 (1H, d, J = 4.6 Hz, H-2′), (OH proton not observed) ppm; ^13^C NMR (CDCl_3_, 126 MHz) δ: 26.8, 26.9 (C-5,7), 27.3 (C-4), 29.9 (CH_3_), 30.0 (CH_3_), 38.5 (C-3), 41.0 (C-6), 55.4 (C-2), 55.7 (OCH_3_), 57.9 (C-8), 59.8 (C-9), 68.2 (CH_2_O), 100.6 (C-5′), 115.2 (C-11), 118.3 (C-3′), 119.3 (CH-triazole), 122.3 (C-7′), 128.0 (C-10′), 131.9 (C-8′), 138.7 (C-4′), 140.4 (C-10), 144.8 (C-9′), 147.3 (C-2′), 155.4 (C-triazole), 158.5 (C-6′) ppm; HR-MS, *m*/*z* (I*_rel._*, %): 434 (1), 433 ([M]^+^, 14), 308 (5), 307 (8), 186 (10), 184 (12), 173 (9), 172 (11), 154 (6), 137 (11), 136 (100), 81 (23), 55 (5), 53 (5), 41 (9); calcd for C_25_H_31_N_5_O_2_: 433.2472; found [M]^+^ *m*/*z*: 433.2473.

Crystallographic data for compound (**4e**): C_25_H_31_N_5_O_2_, *M* = 433.55, orthorhombic, space group *P*2_1_2_1_2_1_, at 296 K: *a* = 10.4845 (5), *b* = 12.1203 (6), *c* = 18.8614 (9) Å, *V* = 2396.8 (2) Å^3^, *Z* = 4, *d*_calc_ = 1.201 g/cm^3^, μ = 0.078 mm^−1^, a total of 23185 *I* (θ_max_ = 26.10°), 4750 unique (*R*_int_ = 0.0297), 3773 *I* > 2σ(*I*), 290 parameters. GooF = 0.996, *R*_1_ = 0.0505, *wR*_2_ = 0.1358 [*I* > 2σ(*I*)], *R*_1_ = 0.0687, *wR*_2_ = 0.1536 (all data), Flack = 0.2 (6), max/min diff. peak 0.445/−0.336 e·Å^−3^.

X-ray crystallographic data were obtained on a Bruker Kappa Apex II diffractometer with CCD using graphite monochromated MoKα radiation (λ = 0.71073 Å). Experimental data reduction was performed using the APEX2 suite (Bruker AXS Inc. APEX2 (Version 2.0), SAINT (Version 8.18c), and SADABS (Version 2.11; Bruker Advanced X-ray Solutions, Madison, WI, USA, 2000–2012). The structures were examined by direct methods and refined by the full-matrix least-squares technique against F^2^ in an anisotropic–isotropic approximation. The H atom positions were calculated with the riding model. All calculations were performed using SHELXT-2014/5 [66] and SHELXL-2018/3 [67]. CCDC 2447839 contains the supplementary crystallographic data for this paper. These data can be obtained free of charge from The Cambridge Crystallographic Data Centre via www.ccdc.cam.ac.uk/data_request/cif (accessed on 19 September 2025) (or from the CCDC, 12 Union Road, Cambridge CB2 1EZ, UK; Fax: +44-1223-336033; E-mail: deposit@ccdc.cam.ac.uk).

*3-((1-((S)-(6-Methoxyquinolin-4-yl)((2R*,*4S*,*5R)-5-vinylquinuclidin-2-yl)methyl)-1H-1*,*2*,*3-triazol-4-yl)methylthio)-1H-1*,*2*,*4-triazol-5-amine* (**8**). Yellow solid; yield 40% (0.28 g); mp 119–122 °C (decomp.); [α]_D_^26^-98.9 (*c* 0.6, CHCl_3_); IR (KBr, ν_max_): 3138, 3307 (N-H), 3074, 1622, 1591, 1508, 758, 715 (C=C), 1085, 1045, 1028 (C-O-C) cm^−1^; ^1^H NMR (CD_3_)_2_SO, 300 MHz) δ: 0.70–0.78 (1H, m, H-7), 1.35–1.78 (4H, m, H-5,4,5,7), 2.20–2.30 (1H, m, H-3), 2.54–2.67 (3H, m, H-2,6,6), 3.02 (1H, dd, J = 13.5, 10.0 Hz, H-2), 3.93 (3H, s, OCH_3_-C-6′), 4.03–4.09 (1H, m, H-8), 4.20 (2H, s, SCH_2_), 5.01 (1H, dd, J = 1.8, 16.6 Hz, H-11), 5.06 (1H, dd, J = 1.8, 12.0 Hz, H-11), 6.03 (1H, ddd, J = 16.6, 12.0, 8.2 Hz, H-10), 6.06 (2H, m, H-10, NH_2_), 6.51 (1H, d, J = 11.3 Hz, H-9), 7.44 (1H, dd, J = 9.2, 2.6 Hz, H-7′), 7.72 (1H, br.s, H-5′), 7.81 (1H, d, J = 4.2 Hz, H-3′), 7.96 (1H, d, J = 9.2 Hz, H-8′), 8.22 (1H, s, H-triazole), 8.80 (1H, d, J = 4.2 Hz, H-2′), 11.99 (1H, br.s, NH) ppm; ^13^C NMR (75 MHz, (CD_3_)_2_SO) δ: 25.6, 26.4, 27.1 (C-5,7, CH_2_S), 27.5 (C-4), 38.4 (C-3), 39.4 (C-6), 55.2 (C-2), 55.9 (OCH_3_), 57.3 (C-8), 59.2 (C-9), 102.2 (C-5′), 114.6 (C-11), 120.7 (C-3′), 121.7 (CH triazole), 123.0 (C-7′), 127.7 (C-10′), 131.5 (C-8′), 140.0 (C-4′), 142.4 (C-10), 144.2 (C-9′), 143.0 (C-1,2,3-triazole), 147.8 (C-2′), 156.7, 157.8 (C-1,2,4-triazole), 157.9 (C-6′) ppm; HR-MS, *m*/*z* (I*_rel._*, %): 504 (2), 503 ([M]^+^, 8), 459 (9), 446 (11), 445 (42), 431 (17), 430 (12), 382 (21), 381 (79), 374 (16), 361 (26), 360 (84), 359 (13), 308 (27), 307 (18), 306 (15), 267 (22), 251 (39), 250 (18), 203 (21), 197 (18), 179 (29), 173 (29), 164 (34), 136 (100), 116 (85), 81 (29), 55 (24), 44 (32), 41 (28), 18 (60); calcd for C_25_H_29_N_9_OS: 503.2210; found [M]^+^ *m*/*z*: 503.2211.

*(1-((S)-(6-Methoxyquinolin-4-yl)((2R*,*4S*,*5R)-5-vinylquinuclidin-2-yl)-methyl)-1H-1*,*2*,*3-triazol-4-yl)methyl 3-tert-butyl-5-ethyl-2-hydroxybenzoate* (**9**). Yellowish solid; yield 47% (0.40 g); mp 84–86 °C (decomp.); [α]_D_^26^-90.5 (*c* 0.5, CHCl_3_); IR (KBr, ν_max_): 3425 (OH), 1666 (C=O), 3078, 3024, 1621,1508, 802, 771, 759, 715 (C=C), 1113, 1047, 1029 (C-O-C) cm^−1^; ^1^H NMR (CDCl_3_, 300 MHz) δ: ^1^H NMR (300 MHz, CDCl_3_) δ: 0.92–0.98 (1H, m, H-7), 1.12 (3H, t, J = 7.2 Hz, CH_3_-*Et*), 1.36 (9H, s, 3× CH_3_), 1.59–1.68 (2H, m, H-5), 1.78–1.82 (1H,m, H-4), 1.83–1.97 (1H, m, H-7), 2.33–2.42 (1H, m, H-3), 2.46 (2H, q, J = 7.2 Hz, CH_2_-*Et*), 2.78–2.91 (2H, m, H-2,6), 3.25 (1H, dd, J = 13.8, 10.5 Hz, H-2), 3.41–3.60 (1H, m, H-6), 3.93 (3H, s, OCH_3_-C-6′), 4.01–4.12 (1H, m, H-8), 5.08 (1H, dd, J = 1.8, 16.8 Hz, H-11), 5.10 (1H, dd, J = 1.8, 11.0 Hz, H-11), 5.37 (2H, ABq, J = 12.2 Hz, ∆ν = 12.8 Hz, CH_2_O), 5.89 (1H, ddd, J = 16.8, 11.0, 7.6 Hz, H-10), 6.51 (1H, d, J = 11.0 Hz, H-9), 7.22 (1H, d, J = 2.2 Hz, H-4 Ar), 7.36 (1H, dd, J = 9.2, 2.6 Hz, H-7′), 7.43 (1H, d, J = 2.2 Hz, H-6 Ar), 7.49 (1H, d, J = 2.6 Hz, H-5′), 7.50 (1H, d, J = 4.2 Hz, H-3′), 7.65 (1H, s, H-triazole), 8.01 (1H, d, J = 9.2 Hz, H-8′), 8.80 (1H, d, J = 4.2 Hz, H-2′), 11.12 (1H, s, OH) ppm; ^13^C NMR (75 MHz, CDCl_3_) δ: 15.3 (CH_3_-*Et*), 26.5, 26.8 (C-5,7), 27.0 (C-4), 27.7 (CH_2_-*Et*), 28.9 (3× CH_3_), 34.5 (C-*t*-Bu), 38.4 (C-3), 40.8 (C-6), 55.3 (C-2), 55.5 (OCH_3_), 57.7 (C-8), 57.8 (OCH_2_), 60.4 (C-9), 100.5 (C-5′), 111.4 (C-1 Ar), 114.8 (C-11), 119.3 (CH triazole), 122.1, 122.9 (C-3′,7′), 125.9 (C-6, Ar), 127.6 (C-10′), 131.7 (C-8′), 133.1 (C-4, Ar), 133.6 (C-5, Ar), 137.1 (C-6, Ar), 138.2 (C-4′), 140.2 (C-triazole), 142.1 (C-10), 144.6 (C-9′), 146.9 (C-2′), 158.4 (C-6′), 158.8 (C-2 Ar) 170.4 (OC=O) ppm; HR-MS, *m*/*z* (I*_rel._*, %): 610 (4), 609 ([M]^+^, 9), 361 (5), 360 (19), 338 (3), 337 (10), 308 (7), 307 (23), 189 (12), 184 (16), 154 (6), 137 (10), 136 (100), 81 (12), 69 (8), 57 (10), 55 (11), 18 (50); calcd for C_36_H_43_N_5_O_4_: 609.3310; found [M]^+^ *m*/*z*: 609.3313.

##### Preparation of Compounds **11**, **12**

To a stirred solution of compound **4g** (2.26 g, 5.56 mmol) and NaH (0.67 g, 19.5 mmol, 70% in oil) in DMF (20 mL) under cooling (5 °C) in argon flow, a solution of propargyl bromide **10** (1.24 g, 8.3 mmol) was added to DMF (5mL). The reaction mixture was stirred at RT for 8 h, diluted with brine (250 mL), and extracted with ethyl acetate (4 × 50 mL). The organic layers were combined, washed with brine (20 mL), dried over magnesium sulfate, and filtered. The solvent was removed under reduced pressure, and the residue was subjected to column chromatography (eluted with chloroform-EtOH, 100:1 → 50:1). Compounds **11** and **12** were sequentially isolated.

*(2R*,*4S*,*5R)-2-((S)-(6-Methoxyquinolin-4-yl)(4-((prop-2-ynyloxy)methyl)-1H-1*,*2*,*3-triazol-1-yl)methyl)-5-vinylquinuclidine* (**11**). Yellow viscous oil; yield 30% (0.77 g); [α]_D_^24^-53.3 (*c* 0.6, CHCl_3_); IR (KBr, ν_max_): 3284 (≡CH), 3080, 1620, 1591, 1508, 823, 714 (C=C), 2119 (C≡C), 1080, 1049, 1016 (C-O-C) cm^−1^; ^1^H NMR (300 MHz, CDCl_3_) δ: 0.84–0.92 (1H, m, H-7), 1.56–1.61 (2H, m, H-5), 1.71–1.80 (1H,m, H-4), 1.83–1.96 (1H, m, H-7), 2.28–2.32 (1H, m, H-3), 2.36 (1H, t, J = 2.3 Hz, C≡CH), 2.64–2.78 (2H, m, H-2,6), 3.17 (1H, dd, J = 13.6, 10.0 Hz, H-2), 3.31–3.50 (1H, m, H-6), 3.75–3.90 (1H, m, H-8), 3.93 (3H, s, OCH_3_-C-6′), 4.11 (2H, ABq, J = 12.2 Hz, ∆ν = 13.0 Hz, OCH_2_C≡), 4.61 (2H, ABq, J = 12.4 Hz, ∆ν = 12.6 Hz, OCH_2_), 5.06 (1H, dd, J = 1.8, 16.8 Hz, H-11), 5.08 (1H, dd, J = 1.8, 10.2 Hz, H-11), 5.90 (1H, ddd, *^3^J* = 16.8, 10.2, 7.5 Hz, H-10), 6.40 (1H, d, J = 11.3 Hz, H-9), 7.35 (1H, dd, J = 9.2, 2.5 Hz, H-7′), 7.46 (1H, s, H-triazole), 7.44 (1H, d, J = 4.6 Hz, H-3′), 7.51 (1H, d, J = 2.5 Hz, H-5′), 8.00 (1H, d, J = 9.2 Hz, H-8′), 8.79 (1H, d, J = 4.6 Hz, H-2′) ppm; ^13^C NMR (100 MHz, CDCl_3_) δ: 27.4, 27.5 (C-5,7), 27.6 (C-4), 38.9 (C-3), 40.8 (C-6), 55.6 (OCH_3_), 55.9 (C-2), 57.4 (OCH_2_), 57.8 (C-8), 60.5 (C-9), 63.3 (OCH_2_), 74.7 (≡CH), 79.0 (C≡), 100.6 (C-5′), 114.6 (C-11), 119.0 (CH triazole), 120.9 (C-3′), 122.9 (C-7′), 127.6 (C-10′), 131.8 (C-8′), 138.9 (C-4′), 141.3 (C-10), 144.1 (C-triazole), 144.9 (C-9′), 147.1 (C-2′), 158.5 (C-6′) ppm; HR-MS, *m*/*z* (I*_rel._*, %): 443 ([M]^+^, 2), 309 (3), 308 (4), 307 (4), 251 (4), 225 (4), 211 (7), 198 (6), 197 (6), 183 (6), 154 (7), 137 (10), 136 (100), 81 (7); calcd for C_26_H_29_N_5_O_2_: 443.2316; found [M]^+^ *m*/*z*: 443.2318.

*(2R*,*4S*,*5R)-2-((R)-(6-Methoxyquinolin-4-yl)(4-((prop-2-ynyloxy)methyl)-1H-1*,*2*,*3-triazol-1-yl)methyl)-5-vinylquinuclidine* (**12**). Yield 0.98 g (40%), Yellowish viscous oil; yield 40% (0.98 g); [α]_D_^24^ +122.9 (c 0.95, CHCl_3_); IR (KBr, ν_max_): 3286 (≡CH), 3076, 1620, 1589, 1508, 794, 715 (C=C), 2114 (C≡C), 1086, 1047, 1030 (C-O-C) cm^−1^; ^1^H NMR (400 MHz, CDCl_3_) δ: 1.37–1.42 (1H, m, H-7), 1.54–1.63 (2H, m, H-5), 1.70–1.80 (1H,m, H-4), 1.83–1.89 (1H, m, H-7), 2.33–2.39 (1H, m, H-3), 2.42 (1H, t, J = 2.3 Hz, C≡CH), 2.60–2.71 (1H, m, H-6), 2.86–2.92 (2H, m,H-2,6), 3.21 (1H, dd, J = 13.6, 10.0 Hz, H-2), 3.85–3.93 (1H, m, H-8), 3.93 (3H, s, OCH_3_-C-6′), 4.16 (2H, ABq, J = 12.2 Hz, ∆ν = 12.0 Hz, OCH_2_C≡), 4.62 (2H, ABq, J = 12.4 Hz, ∆ν = 13.2 Hz, OCH_2_), 5.10 (1H, dd, J = 1.8, 17.2 Hz, H-11), 5.12 (1H, dd, J = 1.8, 11.6 Hz, H-11), 5.93 (1H, ddd, J = 17.2, 11.6, 7.5 Hz, H-10), 6.44 (1H, d, J = 11.3 Hz, H-9), 7.34 (1H, dd, J = 9.2, 2.5 Hz, H-7′), 7.35 (1H, s, H-triazole), 7.41 (1H, d, J = 2.5 Hz, H-5′), 7.69 (1H, d, J = 4.6 Hz, H-3′), 8.00 (1H, d, J = 9.2 Hz, H-8′), 8.86 (1H, d, J = 4.6 Hz, H-2′) ppm; ^13^C NMR (100 MHz, CDCl_3_) δ: 25.1 (C-7), 27.0 (C-4), 27.5 (C-5), 39.3 (C-3), 41.3 (C-6), 55.7 (OCH_3_), 55.9 (C-2), 57.1 (C-8), 57.7 (OCH_2_), 61.4 (C-9), 63.2 (OCH_2_), 75.0 (≡CH), 79.0 (C≡), 100.2 (C-5′), 114.8 (C-11), 119.7 (C-3′), 121.2 (CH triazole), 122.1 (C-7′), 127.8 (C-10′), 131.9 (C-8′), 139.3 (C-4′), 141.5 (C-10), 144.3 (C-9′), 145.0 (C-triazole), 147.4 (C-2′), 158.5 (C-6′) ppm; HR-MS, *m*/*z* (I*_rel._*, %): 443 ([M]^+^, 1), 307 (1), 211 (2), 198 (2), 197 (2), 184 (2), 183 (2), 172 (3), 155 (3), 137 (9), 136 (100), 81 (6); calcd for C_26_H_29_N_5_O_2_: 443.2316; found [M]^+^ *m*/*z*: 443.2314.

##### General Procedure for the A3 Coupling Reaction

Secondary amine (**13**, **16**, **19**–**22**) (4 mmol), 30% aq. formaldehyde (8 mmol, 0.75 mL), and Cu(OAc)_2_ × H_2_O (40 mg, 0.2 mmol) were successively added in argon to a stirred solution of compounds **11** and **12** (0.89 g, 2 mmol) or their mixture (for synthesis of **23**, **27**; **24**, **28**; **25**, **29**; **26**, **30**) in 1,4-dioxane (10 mL). The reaction mixture was stirred at 65–70 °C (bath) for 6 h (TLC-control), followed by being kept at room temperature overnight. The reaction mixture was evaporated, the residue was dissolved in ethyl acetate (100–120 mL), washed with brine (3 × 20mL), dried over NaSO_4_, filtered, and evaporated. Purification was carried out by column chromatography (eluent: chloroform–ethanol, 50:1 → 20:1).

*4-((1-((S)-(6-Methoxyquinolin-4-yl)((2R*,*4S*,*5R)-5-vinylquinuclidin-2-yl)methyl)-1H-1*,*2*,*3-triazol-4-yl)methoxy)-N*,*N-dipropylbut-2-yn-1-amine* (**14**). Yellow viscous oil; yield 80% (0.89 g); [α]_D_^24^-65.2 (*c* 0.7, CHCl_3_); ^1^H NMR (300 MHz, CDCl_3_) δ: 0.81–0.90 (1H, m, H-7), 0.86 (6H, t, J = 7.2 Hz, 2× CH_3_-*Pr*), 1.46 (4H, q, J = 7.2 Hz, 2× CH_2_-*Pr*), 1.56–1.61 (2H, m, H-5), 1.71–1.80 (1H,m, H-4), 1.83–1.96 (1H, m, H-7), 2.29–2.34 (1H, m, H-3), 2.43 (4H, t, J = 7.2 Hz, 2× CH_2_-*Pr*), 2.67–2.80 (2H, m, H-2,6), 3.19 (1H, dd, J = 13.6, 10.0 Hz, H-2), 3.43 (2H, d, J = 2.3 Hz, CH_2_N), 3.32–3.50 (1H, m, H-6), 3.79–3.92 (1H, m, H-8), 3.95 (3H, s, OCH_3_), 4.17 (2H, br.s, CH_2_O), 4.61 (2H, ABq, J = 12.2 Hz, ∆ν = 12.6 Hz, CH_2_O), 5.08 (1H, dd, J = 1.8, 16.8 Hz, H-11), 5.10 (1H, dd, J = 1.8, 10.8 Hz, H-11), 5.91 (1H, ddd, ^3^J = 16.8, 10.8, 7.8 Hz, H-10), 6.42 (1H, d, J = 11.3 Hz, H-9), 7.37 (1H, dd, J = 9.2, 2.5 Hz, H-7′), 7.46 (1H, d, J = 4.6 Hz, H-3′), 7.47 (1H, s, H-triazole), 7.52 (1H, d, J = 2.5 Hz, H-5′), 8.02 (1H, d, J = 9.2 Hz, H-8′), 8.81 (1H, d, J = 4.6 Hz, H-2′) ppm; ^13^C NMR (126 MHz, CDCl_3_) δ: 11.6 (2× CH_3_-*Pr*), 20.2 (2× CH_2_-*Pr*), 27.3, 27.4 (C-5,7), 27.5 (C-4), 38.9 (C-3), 40.8 (C-6), 41.8 (CH_2_), 55.4 (2× CH_2_-*Pr*), 55.6 (OCH_3_), 55.65 (C-2), 57.76 (C-8), 57.9 (OCH_2_), 60.5 (C-9), 63.1 (OCH_2_), 80.3, 81.4 (C≡C), 100.6 (C-5′), 114.6 (C-11), 119.1 (CH triazole), 120.8 (C-3′), 122.2 (C-7′), 128.0 (C-10′), 131.8 (C-8′), 138.9 (C-4′), 141.2 (C-10), 144.4 (C-triazole), 144.8 (C-9′), 147.1 (C-2′), 158.5 (C-6′) ppm; HR-MS, *m*/*z* (I*_rel._*, %): 557 (2), 556 ([M]^+^, 5), 309 (11), 308 (25), 307 (49), 250 (7), 249 (42), 221 (18), 184 (9), 172 (11), 168 (24), 154 (11), 153 (100), 152 (13), 138 (43), 137 (10), 136 (84), 124 (20), 122 (11), 82 (11), 81 (26); calcd for C_33_H_44_N_6_O_2_: 556.3520; found [M]^+^ *m*/*z*: 556.3518.

*4-((1-((R)-(6-Methoxyquinolin-4-yl)((2R*,*4S*,*5R)-5-vinylquinuclidin-2-yl)methyl)-1H-1*,*2*,*3-triazol-4-yl)methoxy)-N*,*N-dipropylbut-2-yn-1-amine* (**15**). Yellow viscous oil; yield 88% (0.98 g); [α]_D_^25^ +101.6 (*c* 1.4, CHCl_3_); ^1^H NMR (400 MHz, CDCl_3_) δ: 0.83 (6H, t, J = 7.2 Hz, 2× CH_3_-*Pr*), 1.34–1.45 (5H, m, H-7 and 2× CH_2_-*Pr*), 1.48–1.59 (1H, m, H-5), 1.68–1.76 (2H, m, H-5,7), 1.79–1.85 (1H,m, H-4), 2.30–2.38 (1H, m, H-3), 2.34 (4H, t, J = 7.2 Hz, 2× CH_2_-*Pr*), 2.56–2.63 (1H, m, H-6), 2.81–2.91 (2H, m, H-2,6), 3.16 (1H, dd, J = 13.6, 10.0 Hz, H-2), 3.37 (2H, t, J = 1.8 Hz, CH_2_N), 3.79–3.92 (1H, m, H-8), 3.95 (3H, s, OCH_3_), 4.14 (2H, t, J = 1.8 Hz, CH_2_O), 4.56 (2H, ABq, J = 12.3 Hz, ∆ν 13 Hz, CH_2_O), 5.04 (1H, dd, J = 1.8, 10.8 Hz, H-11), 5.07 (1H, dd, J = 1.8, 16.2 Hz, H-11), 5.91 (1H, ddd, J = 16.2, 10.8, 7.5 Hz, H-10), 6.39 (1H, d, J = 11.3 Hz, H-9), 7.29 (1H, s, H-triazole), 7.30 (1H, dd, J = 9.2, 2.5 Hz, H-7′), 7.37 (1H, d, J = 2.5 Hz, H-5′), 7.64 (1H, d, J = 4.6 Hz, H-3′), 7.97 (1H, d, J = 9.2 Hz, H-8′), 8.82 (1H, d, J = 4.6 Hz, H-2′) ppm; ^13^C NMR (126 MHz, CDCl_3_) δ: 11.7 (2× CH_3_-*Pr*), 20.4 (2× CH_2_-*Pr*), 24.9 (C-7), 26.9 (C-4), 27.4 (C-5), 39.2 (C-3), 41.2 (C-6), 41.9 (CH_2_), 55.5 (2× CH_2_-*Pr*), 55.6 (OCH_3_), 55.7 (C-2), 56.9 (C-8), 58.0 (OCH_2_), 61.2 (C-9), 62.8 (OCH_2_), 79.5, 82.2 (C≡C), 100.16 (C-5′), 114.8 (C-11), 119.5 (C-3′), 120.9 (CH triazole), 122.0 (C-7′), 127.7 (C-10′), 131.8 (C-8′), 139.2 (C-4′), 141.3 (C-10), 144.8 (C-triazole), 145.3 (C-9′), 147.3 (C-2′), 158.3 (C-6′) ppm; HR-MS, *m*/*z* (I*_rel._*, %): 557 (0.5), 556 ([M]^+^, 1.3), 309 (5), 308 (24), 307 (21), 250 (6), 249 (35), 221 (8), 173 (5), 172 (11), 168 (12), 153 (59), 152 (9), 138 (28), 137 (11), 136 (100), 124 (12), 114 (14), 100 (9), 86 (27), 81 (18), 43 (35); calcd for C_33_H_44_N_6_O_2_: 556.3520; found [M]^+^ *m*/*z*: 556.3521.

*4-((1-((S)-(6-Methoxyquinolin-4-yl)((2R*,*4S*,*5R)-5-vinylquinuclidin-2-yl)methyl)-1H-1*,*2*,*3-triazol-4-yl)methoxy)-N*,*N-diisopropylbut-2-yn-1-amine* (**17**). Yellow viscous oil; yield 65% (0.72 g); [α]_D_^24^-67 (*c* 0.9, CHCl_3_); ^1^H NMR (500 MHz, CDCl_3_) δ: 0.83–0.91 (1H, m, H-7), 1.03 (12H, d, J = 6.5 Hz, 4× CH_3_-*i-Pr*), 1.56–1.61 (2H, m, H-5), 1.71–1.80 (1H,m, H-4), 1.84–1.96 (1H, m, H-7), 2.29–2.34 (1H, m, H-3), 2.65–2.75 (2H, m, H-2,6), 3.09–3.21 (3H, m, 2× CH-*i-Pr* + H-2), 3.39 (2H, br.s, CH_2_N), 3.35–3.50 (1H, m, H-6), 3.79–3.92 (1H, m, H-8), 3.93 (3H, s, OCH_3_), 4.11 (2H, br.s, CH_2_O), 4.59 (2H, ABq, J = 12.4 Hz, ∆ν = 12.6 Hz, CH_2_O), 5.06 (1H, dd, J = 1.8, 16.8 Hz, H-11), 5.08 (1H, dd, J = 1.8, 10.8 Hz, H-11), 5.90 (1H, ddd, ^3^J = 16.8, 10.8, 7.5 Hz, H-10), 6.42 (1H, d, J = 11.3 Hz, H-9), 7.35 (1H, dd, J = 9.2, 2.5 Hz, H-7′), 7.43 (1H, s, H-triazole), 7.44 (1H, d, J = 4.6 Hz, H-3′), 7.52 (1H, d, J = 2.5 Hz, H-5′), 8.00 (1H, d, J = 9.2 Hz, H-8′), 8.79 (1H, d, J = 4.6 Hz, H-2′) ppm; ^13^C NMR (75 MHz, CDCl_3_) δ: 20.4 (4× CH_3_-*i-Pr*), 27.47, 27.52 (C-5,7), 27.6 (C-4), 34.2 (CH_2_), 39.0 (C-3), 40.9 (C-6), 48.3 (2× CH-*i*-*Pr*), 55.7 (OCH_3_), 55.9 (C-2), 57.8 (C-8), 58.2 (OCH_2_), 60.5 (C-9), 63.1 (OCH_2_), 79.3, 86.1 (C≡C), 100.6 (C-5′), 114.6 (C-11), 119.1 (C-3′), 120.8 (CH triazole), 122.3 (C-7′), 128.1 (C-10′), 131.9 (C-8′), 138.9 (C-4′), 141.3 (C-10), 144.6 (C-triazole), 144.9 (C-9′), 147.2 (C-2′), 158.5 (C-6′) ppm; HR-MS, *m*/*z* (I*_rel._*, %): 556 ([M]^+^, 0.6), 513 (12), 308 (8), 307 (21), 249 (20), 184 (5), 173 (5), 172 (7), 168 (7), 155 (5), 153 (35), 152 (10), 138 (22), 137 (11), 136 (100), 100 (14), 84 (11), 81 (11), 42 (14); calcd for C_33_H_44_N_6_O_2_: 556.3520; found [M]^+^ *m*/*z*: 556.3526.

*4-((1-((R)-(6-Methoxyquinolin-4-yl)((2R*,*4S*,*5R)-5-vinylquinuclidin-2-yl)methyl)-1H-1*,*2*,*3-triazol-4-yl)methoxy)-N*,*N-diisopropylbut-2-yn-1-amine* (**18**). Yellow viscous oil; yield 42% (0.47 g); [α]_D_^25^ +104.76 (*c* 0.6, CHCl_3_); ^1^H NMR (400 MHz, CDCl_3_) δ: 1.04 (6H, t, J = 6.7 Hz, 2× CH_3_-*Pr*), 1.32–1.40 (1H, m, H-7), 1.48–1.59 (2H, m, H-5,7), 1.68–1.76 (1H, m, H-5), 1.79–1.85 (1H,m, H-4), 2.30–2.35 (1H, m, H-3), 2.56–2.63 (1H, m, H-6), 2.81–2.91 (2H, m, H-2,6), 3.10–3.20 (3H, m, 2× CH-*i-Pr* + H-2), 3.40 (2H, t, J = 1.8 Hz, CH_2_N), 3.80–3.90 (1H, m, H-8), 3.98 (3H, s, OCH_3_), 4.11 (2H, t, J = 1.8 Hz, CH_2_O), 4.57 (2H, ABq, J = 12.2 Hz, ∆ν = 13.8 Hz, CH_2_O), 5.04 (1H, dd, J = 1.8, 16.4 Hz, H-11), 5.02 (1H, dd, J = 1.8, 12.2 Hz, H-11), 5.95 (1H, ddd, ^3^J = 16.4, 12.2, 7.7 Hz, H-10), 6.39 (1H, d, J = 11.3 Hz, H-9), 7.29 (1H, s, H-triazole), 7.30 (1H, dd, J = 9.2, 2.5 Hz, H-7′), 7.37 (1H, d, J = 2.5 Hz, H-5′), 7.64 (1H, d, J = 4.6 Hz, H-3′), 7.96 (1H, d, J = 9.2 Hz, H-8′), 8.82 (1H, d, J = 4.6 Hz, H-2′) ppm; ^13^C NMR (126 MHz, CDCl_3_) δ: 20.3 (4× CH_3_-*i*-*Pr*), 24.9 (C-7), 27.0 (C-4), 27.5 (C-5), 34.2 (CH_2_), 39.2 (C-3), 41.2 (C-6), 48.5 (2× CH-*i*-*Pr*), 55.5 (2× CH_2_-*Pr*), 55.6 (OCH_3_), 55.8 (C-2), 57.0 (C-8), 58.2 (OCH_2_), 61.3 (C-9), 62.9 (OCH_2_), 78.5, 85.7 (C≡C), 100.1 (C-5′), 114.8 (C-11), 119.5 (C-3′), 121.0 (CH triazole), 122.1 (C-7′), 127.8 (C-10′), 131.9 (C-8′), 139.2 (C-4′), 141.3 (C-10), 144.9 (C-triazole), 145.4 (C-9′), 147.4 (C-2′), 158.3 (C-6′) ppm; HR-MS, *m*/*z* (I*_rel._*, %): 557 (12), 556 ([M]^+^, 1.6), 513 (13), 309 (8), 308 (34), 307 (41), 291 (6), 267 (11), 250 (15), 249 (88), 172 (8), 168 (13), 154 (12), 153 (100), 152 (22), 151 (9), 138 (54), 137 (9), 136 (85), 110 (16), 100 (28), 94 (8), 84 (10), 81 (12), 42 (12); calcd for C_33_H_44_N_6_O_2_: 556.3520; found [M]^+^ *m*/*z*: 556.3521.

*(2R*,*4S*,*5R)-2-((S)-(6-Methoxyquinolin-4-yl)(4-((4-(pyrrolidin-1-yl)but-2-ynyloxy)methyl)-1H-1*,*2*,*3-triazol-1-yl)methyl)-5-vinylquinuclidine* (**23**). Yellowish viscous oil; yield 34% (0.36g); [α]_D_^24^-68.8 (*c* 1.9, CHCl_3_); IR (KBr, ν_max_): 3133 (≡CH), 3076, 1622, 1593, 1510, 759, 713 (C=C), 1080, 1047, 1028 (C-O-C) cm^−1^; ^1^H NMR (CDCl_3_, 400 MHz), δ: 0.84–0.91 (1H, m, H-7_b_), 1.58–1.62 (2H, m, H-5,7), 1.73–1.78 (4H, m, H-3,4-pyrrolidine), 1.84–1.94 (2H, m, H-4,5), 2.29–2.33 (1H, m, H-3), 2.53–2.57 (4H, m, H-2,5-pyrrolidine), 2.66–2.75 (м, 2H, H-2,6), 3.17 (1 H, dd, J = 13.6, 10 Hz, H-2_a_), 3.36–3.39 (2H, m, CH_2_N), 3.36–3.44 (1H, m, H-6_a_), 3.81–3.90 (1H, m, H-8), 3.93 (3H, s, OCH_3_), 4.13–4.16 (2H, m, CH_2_), 4.60 (2H, ABq, J = 12.1 Hz, ∆ν = 12.6 Hz, CH_2_O), 5.07 (1H, dd, J = 1.8, 17.0 Hz, H-11), 5.08 (1H, dd, *J* = 1.8, 10.6 Hz, H-11), 5.88 (1H, ddd, J = 17.0, 10.6, 7.5 Hz, H-10), 6.39 (1H, d, J = 11.3 Hz, H-9), 7.33 (1H, dd, J = 9.1, 2.5 Hz, H-7′), 7.43 (1H, d, J = 4.6 Hz, H-3′), 7.45 (1H, s, H-triazole), 7.51 (1H, d, J = 2.5 Hz, H-5′), 8.00 (1H, d, J = 9.1 Hz, H-8′), 8.79 (1H, d, J = 4.6 Hz, H-2′) ppm; ^13^C NMR (CDCl_3_, 101 MHz) δ: 23.6 (C-3,4-pyrrolidine), 27.4, 27.5, 27.6 (C-4,5,7), 39.0 (C-3), 40.8 (C-6), 43.2 (NCH_2_), 52.5 (C-2,5-pyrrolidine), 55.7 (OCH_3_), 55.9 (C-2), 57.9 (C-8), 58.0 (CH_2_), 60.6 (C-9), 63.1 (OCH_2_), 79.4, 82.8 (C≡C), 100.7 (C-5′), 114.7 (C-11), 119.1 (C-3′), 120.9 (CH-triazole), 122.3 (C-7′), 128.1 (C-10′), 131.9 (C-8′), 139.0 (C-4′), 141.4 (C-10), 144.5 (C-9′), 145.0 (C-triazole), 147.2 (C-2′), 158.6 (C-6′) ppm; HR-MS, *m*/*z* (I*_rel._*, %): 526 ([M]^+^, 2), 471 (8), 307 (22), 219 (20), 191 (12), 172 (11), 138 (13), 137 (11), 136 (100), 123 (32), 81 (9); calcd for C_31_H_38_ N_6_O_2_: 526.3051; found [M]^+^ *m*/*z*: 526.3049.

*(2R*,*4S*,*5R)-2-((R)-(6-Methoxyquinolin-4-yl)(4-((4-(pyrrolidin-1-yl)but-2-ynyloxy)-methyl)-1H-1*,*2*,*3-triazol-1-yl)methyl)-5-vinylquinuclidine* (**27**). Yellow viscous oil; yield 44% (0.46 g); [α]_D_^24^ +99.02 (*c* 2.0, CHCl_3_); IR (KBr, ν_max_): 3145 (≡CH), 3078, 1622, 1593, 1510, 715 (C=C), 1084, 1047, 1030 (C-O-C) cm^−1^; ^1^H NMR (CDCl_3_, 400 MHz), δ: 1.34–1.45 (1H, m, H-7), 1.52–1.60 (2H, m, H-5,7), 1.73–1.78 (4H, m, H-3,4-pyrrolidine), 1.78–1.86 (2H, m, H-4,5), 2.29–2.33 (1H, m, H-3), 2.53–2.57 (4H, m, H-2,5-pyrrolidine), 2.62 (1H, m, H-6b); 2.76–2.97 (м, 2H, H-2,6), 3.17 (1 H, dd, J = 13.6, 10 Hz, H-2_a_), 3.36–3.39 (2H, m, CH_2_N), 3.81–3.90 (1H, m, H-8), 3.89 (3H, s, OCH_3_), 4.12–4.16 (2H, m, CH_2_), 4.57 (2H, ABq, J = 12.1 Hz, ∆ν = 14.9 Hz, CH_2_O), 5.06 (1H, dd, J = 1.8, 10.2 Hz, H-11), 5.07 (1H, dd, J = 1.8, 17.2 Hz, H-11), 5.89 (1H, ddd, J = 17.2, 10.2, 7.5 Hz, H-10), 6.39 (1H, d, J = 11.3 Hz, H-9), 7.30 (1H, dd, J = 9.0, 2.5 Hz, H-7′); 7.30 (1H, s, H-triazole), 7.37 (1H, d, J = 2.5 Hz, H-5′), 7.64 (1H, d, J = 4.6 Hz, H-3′), 7.97 (1H, d, J = 9.2 Hz, H-8′), 8.82 (1H, d, J = 4.6 Hz, H-2′) ppm; ^13^C NMR (CDCl_3_, 100 MHz) δ: 23.5 (C-3,4-pyrrolidine), 25.0 (C-7), 27.0 (C-4), 27.5 (C-5), 39.3 (C-3), 41.2 (C-6), 43.2 (NCH_2_), 52.6 (C-2,5-pyrrolidine), 55.6 (OCH_3_), 55.9 (C-2), 57.0 (C-8), 58.0 (CH_2_), 61.3 (C-9), 62.9 (OCH_2_), 79.1, 83.0 (C≡C), 100.2 (C-5′), 114.8 (C-11), 119.8 (C-3′), 120.9 (CH-triazole), 122.1 (C-7′), 127.8 (C-10′), 131.9 (C-8′), 139.3 (C-4′), 141.4 (C-10), 144.9 (C-9′), 145.4 (C-triazole), 147.4 (C-2′), 158.3 (C-6′) ppm; HR-MS, *m*/*z* (I*_rel._*, %): 526 ([M]^+^, 0.8), 471 (1), 308 (21), 307 (20), 220 (8), 219 (57), 138 (8), 137 (11), 136 (100), 123 (35), 81 (12); calcd for C_31_H_38_ N_6_O_2_: 526.3051; found [M]^+^ *m*/*z*: 526.3042.

*(2R*,*4S*,*5R)-2-((S)-(6-Methoxyquinolin-4-yl)(4-((4-(piperidin-1-yl)but-2-ynyloxy)methyl)-1H-1*,*2*,*3-triazol-1-yl)methyl)-5-vinylquinuclidine* (**24**). Yellow amorphous solid; yield 34% (0.37g); [α]_D_^24^-72.0 (*c* 1.4, CHCl_3_); IR (KBr, ν_max_): 3133 (≡CH), 3076, 1622, 1593, 1508, 758, 714 (C=C), 1080, 1047, 1028 (C-O-C) cm^−1^; ^1^H NMR (CDCl_3_, 300 MHz), δ: 0.82–0.91 (1H, m, H-7), 1.30–1.45 (2H, m, 2H-4-piperidine), 1.51–1.62 (6H, m, 2-H-5, 2H-3,5-piperidine), 1.74–1.78 (1H, m, H-4), 1.84–1.94 (1H, m, H-7_b_), 2.29–2.33 (1H, m, H-3), 2.38–2.47 (4H, m, 2× H-2,6-piperidine), 2.66–2.75 (м, 2H, H-2,6), 3.17 (1H, dd, J = 13.6, 10 Hz, H-2_a_), 3.22 (2H, m, CH_2_N), 3.34–3.44 (1H, m, H-6_a_), 3.80–3.89 (1H, m, H-8), 3.93 (3H, s, OCH_3_), 4.13–4.16 (2H, m, CH_2_), 4.61 (2H, ABq, J = 12.3 Hz, ∆ν = 14.0 Hz, CH_2_O), 5.07 (1H, dd, J = 1.8, 17.2 Hz, H-11), 5.08 (1H, dd, *J* = 1.8, 10.8 Hz, H-11), 5.92 (1H, ddd, J = 17.2, 10.8, 7.5 Hz, H-10), 6.39 (1H, d, J = 11.3 Hz, H-9), 7.35 (1H, dd, J = 9.2, 2.5 Hz, H-7′), 7.43 (1H, d, J = 4.6 Hz, H-3′), 7.45 (1H, s, H-triazole), 7.50 (1H, d, J = 2.5 Hz, H-5′), 8.00 (1H, d, J = 9.2 Hz, H-8′), 8.79 (1H, d, J = 4.6 Hz, H-2′) ppm; ^13^C NMR (CDCl_3_, 126 MHz) δ: 23.7 (C-4 piperidine), 25.7 (C-3,5-piperidine), 27.46, 27.48, 27.52 (C-4,5,7), 39.0 (C-3), 40.8 (C-6), 47.7 (NCH_2_), 53.2 (C-2,6-piperidine), 55.7 (OCH_3_), 55.9 (C-2), 57.8 (C-8), 58.0 (CH_2_), 60.4 (C-9), 63.2 (OCH_2_), 80.0, 82.3 (C≡C), 100.5 (C-5′), 114.7 (C-11), 119.0 (C-3′), 120.8 (CH-triazole), 122.4 (C-7′), 128.1 (C-10′), 131.8 (C-8′), 138.8 (C-4′), 141.2 (C-10), 144.5 (C-9′), 145.9 (C-triazole), 147.1 (C-2′), 158.6 (C-6′) ppm; HR-MS, *m*/*z* (I*_rel._*, %): 541 (2), 540 ([M]^+^, 5), 485 (7), 308 (15), 307 (23), 233 (28), 205 (30), 198 (12), 173 (13), 172 (12), 152 (18), 138 (11), 137 (100), 136 (99), 122 (34), 85 (27), 83 (38); calcd for C_32_H_40_N_6_O_2_: 540.3207; found [M]^+^ *m*/*z*: 540.3201.

*(2R*,*4S*,*5R)-2-((R)-(6-Methoxyquinolin-4-yl)(4-((4-(piperidin-1-yl)but-2-ynyloxy)methyl)-1H-1*,*2*,*3-triazol-1-yl)methyl)-5-vinylquinuclidine* (**28**). Yellow amorphous solid; yield 40% (0.43 g); [α]_D_^24^ +101.9 (*c* 1.4, CHCl_3_); IR (KBr, ν_max_): 3132 (≡CH), 3074, 1622, 1593, 1508, 792, 756, 715 (C=C), 1084, 1045, 1028 (C-O-C) cm^−1^; ^1^H NMR (CDCl_3_, 300 MHz), δ: 1.30–1.38 (3H, m, H-7 and 2H-4-piperidine), 1.50–1.60 (6H, m, H-5,7, 2H-3,5-piperidine), 1.73–1.78 (1H, m, H-5), 1.78–1.84 (1H, m, H-4), 2.29–2.33 (1H, m, H-3), 2.40–2.48 (4H, m, H-2,6-piperidine), 2.56–2.66 (1H, m, H-6_b_), 2.79–2.95 (м, 2H, H-2,6), 3.16 (1 H, dd, J = 13.6, 10 Hz, H-2_a_), 3.20–3.23 (2H, m, CH_2_N), 3.81–3.90 (1H, m, H-8), 3.88 (3H, s, OCH_3_), 4.12–4.15 (2H, m, CH_2_), 4.57 (2H, ABq, J = 12.4 Hz, ∆ν = 14.2 Hz, CH_2_O), 5.04 (1H, dd, J = 1.8, 10.2 Hz, H-11), 5.07 (1H, dd, J = 1.8, 17.2 Hz, H-11), 5.89 (1H, ddd, J = 17.2, 10.2, 7.5 Hz, H-10), 6.39 (1H, d, J = 11.2 Hz, H-9), 7.30 (1H, dd, J = 9.2, 2.5 Hz, H-7′); 7.30 (1H, s, H-triazole), 7.37 (1H, d, J = 2.5 Hz, H-5′), 7.64 (1H, d, J = 4.6 Hz, H-3′), 7.96 (1H, d, J = 9.2 Hz, H-8′), 8.82 (1H, d, J = 4.6 Hz, H-2′) ppm; ^13^C NMR (CDCl_3_, 126 MHz) δ: 23.6 (C-4-piperidine), 24.9 (C-7), 25.6 (C-3,5-piperidine), 26.9 (C-4), 27.4 (C-5), 39.2 (C-3), 41.1 (C-6), 47.7 (NCH_2_), 53.2 (C-2,6-piperidine), 55.6 (OCH_3_), 55.7 (C-2), 56.9 (C-8), 58.0 (CH_2_), 61.2 (C-9), 62.9 (OCH_2_), 79.7, 82.6 (C≡C), 100.1 (C-5′), 114.7 (C-11), 119.6 (C-3′), 121.1 (CH-triazole), 122.0 (C-7′), 127.7 (C-10′), 131.8 (C-8′), 139.2 (C-4′), 141.3 (C-10), 144.8 (C-9′), 145.3 (C-triazole), 147.3 (C-2′), 158.3 (C-6′) ppm; HR-MS, *m*/*z* (I*_rel._*, %): 541 (0.7), 540 ([M]^+^, 2), 308 (33), 307 (14), 267 (12), 233 (60), 205 (10), 173 (9), 172 (10), 152 (11), 138 (10), 137 (100), 136 (66), 122 (28), 84 (13), 81 (15); calcd for C_32_H_40_N_6_O_2_: 540.3207; found [M]^+^ *m*/*z*: 540.3205.

*(2R*,*4S*,*5R)-2-((S)-(4-((4-(Azepan-1-yl)but-2-ynyloxy)methyl)-1H-1*,*2*,*3-triazol-1-yl)(6-methoxyquinolin-4-yl)methyl)-5-vinylquinuclidine* (**25**). Yellow amorphous solid; yield 34% (0.38 g); [α]_D_^24^-65.1 (*c* 1.4, CHCl_3_); IR (KBr, ν_max_): 3135 (≡CH), 3076, 1622, 1593, 1510, 715 (C=C), 1082, 1049, 1028 (C-O-C) cm^−1^; ^1^H NMR (CDCl_3_, 300 MHz), δ: 0.84–0.91 (1H, m, H-7_b_), 1.50–1.70 (10H, m, H-5,7 and 8H, H-3,4,5,6-azepane), 1.71–1.75 (1H, m, H-5), 1.72–1.82 (1H, m, H-4), 2.29–2.33 (1H, m, H-3), 2.61–2.71 (4H, m, H-2,7-azepane), 2.72–2.77 (м, 2H, H-2,6), 3.17 (1H, dd, J = 13.8, 10.0 Hz, H-2_a_), 3.22 (2H, m, CH_2_N), 3.34–3.44 (1H, m, H-6_a_), 3.80–3.89 (1H, m, H-8), 3.93 (3H, s, OCH_3_), 4.13–4.16 (2H, m, CH_2_), 4.61 (2H, ABq, J = 12.3 Hz, ∆ν = 13.6 Hz, CH_2_O), 5.07 (1H, dd, J = 1.8, 17.0 Hz, H-11), 5.08 (1H, dd, J = 1.8, 11.2 Hz, H-11), 5.92 (1H, ddd, J = 17.0, 11.2, 7.5 Hz, H-10), 6.39 (1H, d, J = 11.3 Hz, H-9), 7.35 (1H, dd, J = 9.2, 2.5 Hz, H-7′), 7.43 (1H, d, J = 4.6 Hz, H-3′), 7.45 (1H, s, H-triazole), 7.51 (1H, d, J = 2.5 Hz, H-5′), 8.00 (1H, d, J = 9.2 Hz, H-8′), 8.79 (1H, d, J = 4.6 Hz, H-2′) ppm; ^13^C NMR (CDCl_3_, 101 MHz) δ: 26.3 (C-4,5-azepane), 27.22, 27.29, 27.35 (C-4,5,7), 27.6 (C-3,6-azepane), 38.8 (C-3), 40.6 (C-6), 47.9 (NCH_2_), 54.9 (C-2,7-azepane), 55.5 (OCH_3_), 55.7 (C-2), 57.6 (C-8), 57.8 (CH_2_), 60.3 (C-9), 62.9 (OCH_2_), 79.2, 82.8 (C≡C), 100.4 (C-5′), 114.4 (C-11), 118.9 (C-3′), 120.6 (CH-triazole), 122.1 (C-7′), 127.9 (C-10′), 131.6 (C-8′), 138.7 (C-4′), 141.0 (C-10), 144.3, 144.7 (C-9′,C-triazole), 147.0 (C-2′), 158.3 (C-6′) ppm; HR-MS, *m*/*z* (I*_rel._*, %): 555 (1), 554 ([M]^+^, 3), 308 (14), 307 (22), 247 (27), 219 (22), 173 (16), 172 (10), 166 (21), 152 (12), 151 (100), 137 (29), 136 (82), 122 (10), 81 (16), 55 (12); calcd for C_33_H_42_N_6_O_2_: 554.3364; found [M]^+^ *m*/*z*: 554.3354.

*(2R*,*4S*,*5R)-2-((R)-(4-((4-(Azepan-1-yl)but-2-ynyloxy)methyl)-1H-1*,*2*,*3-triazol-1-yl)(6-methoxyquinolin-4-yl)methyl)-5-vinylquinuclidine* (**29**). Yellow amorphous solid; yield 50% (0.54 g); [α]_D_^24^ +91.4 (*c* 0.9, CHCl_3_); IR (KBr, ν_max_): 3140 (≡CH), 3076, 1622, 1593, 1510, 795, 715 (C=C), 2240 (C≡C), 1084, 1045, 1030 (C-O-C) cm^−1^; ^1^H NMR (CDCl_3_, 300 MHz) δ: ^1^H NMR (CDCl_3_, 300 MHz), δ: 1.32–1.39 (1H, m, H-7), 1.46–1.67 (10H, m, H-5,7, 8H-3,4,5,6- azepane), 1.72–1.78 (1H, m, H-5), 1.79–1.84 (1H, m, H-4), 2.29–2.33 (1H, m, H-3), 2.58–2.63 (5H, m, H-6_b_ and 4H-2,7-azepane), 2.80–2.95 (м, 2H, H-2,6), 3.16 (1 H, dd, J = 13.6, 10 Hz, H-2_a_), 3.33 (2H, br.s, CH_2_N), 3.81–3.90 (1H, m, H-8), 3.88 (3H, s, OCH_3_), 4.14 (2H, br.s, CH_2_), 4.57 (2H, ABq, J = 12.2 Hz, ∆ν = 14.2 Hz, CH_2_O), 5.05 (1H, dd, J = 1.8, 10.0 Hz, H-11), 5.06 (1H, dd, J = 1.8, 17.0 Hz, H-11), 5.89 (1H, ddd, J = 17.0, 10.0, 7.5 Hz, H-10), 6.39 (1H, d, J = 11.2 Hz, H-9), 7.30 (1H, dd, J = 9.2, 2.5 Hz, H-7′), 7.30 (1H, s, H-triazole), 7.37 (1H, d, J = 2.5 Hz, H-5′), 7.64 (1H, d, J = 4.6 Hz, H-3′), 7.96 (1H, d, J = 9.2 Hz, H-8′), 8.82 (1H, d, J = 4.6 Hz, H-2′) ppm; ^13^C NMR (CDCl_3_, 126 MHz) δ: 27.0 (C-7), 26.5 (C-4,5-azepane), 27.0 (C-4), 27.5 (C-5), 27.9 (C-3,6-azepane), 39.2 (C-3), 41.2 (C-6), 48.1 (NCH_2_), 55.2 (C-2,7-azepane), 55.6 (OCH_3_), 55.8 (C-2), 57.1 (C-8), 58.0 (CH_2_), 61.4 (C-9), 62.9 (OCH_2_), 79.0, 83.4 (C≡C), 100.1 (C-5′), 114.8 (C-11), 119.7 (C-3′), 121.1 (CH-triazole), 122.0 (C-7′), 127.8 (C-10′), 131.8 (C-8′), 139.3 (C-4′), 141.3 (C-10), 144.9 (C-9′), 145.4 (C-triazole), 147.4 (C-2′), 158.3 (C-6′) ppm; HR-MS, *m*/*z* (I*_rel._*, %): 555 (0.5), 554 ([M]^+^, 0.5), 308 (17), 307 (12), 247 (33), 219 (9), 184 (10), 173 (11), 172 (12), 166 (11), 151 (72), 137 (26), 136 (100), 108 (14), 95 (15), 82 (16), 81 (31); calcd for C_33_H_42_N_6_O_2_: 554.3364; found [M]^+^ *m*/*z*: 554.3358.

*(2R*,*4S*,*5R)-2-((S)-(4-((4-(Azocan-1-yl)but-2-ynyloxy)methyl)-1H-1*,*2*,*3-triazol-1-yl)(6-methoxyquinolin-4-yl)methyl)-5-vinylquinuclidine* (**26**). Yellowish viscous oil; yield 40% (0.45 g); [α]_D_^24^-64.1 (*c* 1.6, CHCl_3_); ^1^H NMR (CDCl_3_, 400 MHz), δ: 0.82–0.91 (1H, m, H-7_b_), 1.51–1.62 (12H, m, H-5,7 and 10H, H-3,4,5,6,7-azocane), 1.73–1.77 (1H, m, H-4), 1.88–1.92 (1H, m, H-5), 2.29–2.33 (1H, m, H-3), 2.52–2.64 (4H, m, H-2,8-azocane), 2.63–2.77 (м, 2H, H-2,6), 3.17 (1H, dd, J = 13.8, 10.0 Hz, H-2_a_), 3.31 (2H, m, CH_2_N), 3.34–3.38 (1H, m, H-6_a_), 3.80–3.89 (1H, m, H-8), 3.93 (3H, s, OCH_3_), 4.12–4.15 (2H, m, CH_2_), 4.60 (2H, ABq, J = 12.0 Hz, ∆ν = 12.8 Hz, CH_2_O), 5.07 (1H, dd, J = 1.8, 17.0 Hz, H-11), 5.08 (1H, dd, J = 1.8, 11.0 Hz, H-11), 5.90 (1H, ddd, J = 17.0, 11.0, 7.5 Hz, H-10), 6.39 (1H, d, J = 11.5 Hz, H-9), 7.35 (1H, dd, J = 9.2, 2.5 Hz, H-7′), 7.43 (1H, d, J = 4.6 Hz, H-3′), 7.44 (1H, s, H-triazole), 7.50 (1H, d, J = 2.5 Hz, H-5′), 8.00 (1H, d, J = 9.2 Hz, H-8′), 8.79 (1H, d, J = 4.6 Hz, H-2′) ppm; ^13^C NMR (CDCl_3_, 75 MHz) δ: 25.9 (C-4,5,6-azocane), 27.4 (C-7), 27.49 (C-3,7-azocane, 27.56 (C-4), 27.61 (C-5), 39.0 (C-3), 40.9 (C-6), 47.7 (NCH_2_), 53.0 (C-2,8-azocane), 55.7 (OCH_3_), 56.0 (C-2), 57.9 (C-8), 58.0 (CH_2_), 60.5 (C-9), 63.1 (OCH_2_), 78.7, 84.0 (C≡C), 100.7 (C-5′), 114.7 (C-11), 119.1 (C-3′), 120.8 (CH-triazole), 122.4 (C-7′), 128.2 (C-10′), 131.9 (C-8′), 138.9 (C-4′), 141.3 (C-10), 144.6, 144.9 (C-9′,C-triazole), 147.2 (C-2′), 158.3 (C-6′) ppm; HR-MS, *m*/*z* (I*_rel._*, %): 569 (10), 568 ([M]^+^, 5), 308 (16), 307 (18), 262 (7), 261 (38), 233 (20), 180 (19), 166 (11), 165 (100), 150 (14), 136 (33), 95 (9), 81 (10), 55 (11); calcd for C_34_H_44_N_6_O_2_: 568.3520; found [M]^+^ *m*/*z*: 568.3522.

*(2R*,*4S*,*5R)-2-((R)-(4-((4-(Azocan-1-yl)but-2-ynyloxy)methyl)-1H-1*,*2*,*3-triazol-1-yl)(6-methoxyquinolin-4-yl)methyl)-5-vinylquinuclidine* (**30**). Yellowish viscous oil; yield 40% (0.45 g); [α]_D_^24^ +65.2 (*c* 1.6, CHCl_3_); ^1^H NMR (CDCl_3_, 300 MHz) δ: ^1^H NMR (CDCl_3_, 300 MHz), δ: 1.32–1.38 (1H, m, H-7), 1.48–1.57 (12H, m, H-5,7, 10H-3,4,5,6,7-azocane), 1.70–1.78 (1H, m, H-5), 1.79–1.83 (1H, m, H-4), 2.29–2.34 (1H, m, H-3), 2.50–2.55 (4H, m, H-2,8-azocane), 2.57–2.63 (1H, m, H-6_b_), 2.80–2.92 (м, 2H, H-2,6), 3.16 (1 H, dd, J = 13.6, 10 Hz, H-2_a_), 3.31 (2H, m, CH_2_N), 3.78–3.88 (1H, m, H-8), 3.89 (3H, s, OCH_3_), 4.14 (2H, m, CH_2_), 4.57 (2H, ABq, J = 12.4 Hz, ∆ν = 14.2 Hz, CH_2_O), 5.05 (1H, dd, J = 1.8, 10.2 Hz, H-11), 5.07 (1H, dd, J = 1.8, 17.2 Hz, H-11), 5.89 (1H, ddd, J = 17.2, 10.2, 7.5 Hz, H-10), 6.39 (1H, d, J = 11.2 Hz, H-9), 7.31 (1H, s, H-triazole), 7.33 (1H, dd, J = 9.2, 2.5 Hz, H-7′), 7.37 (1H, d, J = 2.5 Hz, H-5′), 7.64 (1H, d, J = 4.6 Hz, H-3′), 7.96 (1H, d, J = 9.2 Hz, H-8′), 8.82 (1H, d, J = 4.6 Hz, H-2′) ppm; ^13^C NMR (CDCl_3_, 126 MHz) δ: 24.7 (C-7), 25.6 (C-4,5,6-azocane), 26.8 (C-4), 27.2 (C-5), 27.39 (C-3,7-azocane), 39.0 (C-3), 41.0 (C-6), 47.4 (NCH_2_), 52.8 (C-2,8-azepane), 55.4 (OCH_3_), 55.6 (C-2), 56.8 (C-8), 57.9 (CH_2_), 61.1 (C-9), 62.6 (OCH_2_), 78.2, 84.0 (C≡C), 99.9 (C-5′), 114.6 (C-11), 119.5 (C-3′), 120.9 (CH-triazole), 121.9 (C-7′), 127.7 (C-10′), 131.6 (C-8′), 139.0 (C-4′), 141.2 (C-10), 144.6 (C-9′), 145.2 (C-triazole), 147.2 (C-2′), 158.1 (C-6′) ppm; HR-MS, *m*/*z* (I*_rel._*, %): 569 (0.5), 568 ([M]^+^, 0.7), 308 (22), 307 (17), 267 (11), 262 (14), 261 (68), 180 (16), 172 (12), 166 (11), 165 (88), 150 (21), 137 (13), 136 (100), 112 (11), 95 (14), 82 (13), 81 (25), 55 (28), 41 (23); calcd for C_34_H_44_N_6_O_2_: 568.3520; found [M]^+^ *m*/*z*: 568.3524.

### 3.2. The Study of Antibacterial Activity

Test microorganisms were obtained from the American Type Culture Collection (ATCC, Rockville, MD, USA) and from the collection of the State Research Center of Applied Microbiology and Biotechnology (Obolensk, Russia). A Gram-negative bacterium, *Escherichia coli* (*ATCC 25922*), as well as three Gram-positive bacteria, *Staphylococcus aureus* (ATCC 6538), *Bacillus subtilis* (ATTC 6633), and *Bacillus cereus* (ATCC 10702), were used.

*For the disc diffusion assay* [68], sterile Mueller–Hinton agar was dispensed in sterile Petri dishes (90 mm diameter) and left at RT to solidify for 2 h. Paper discs (6 mm diameter) were placed on the agar surface, and 50 µL of each compound was placed on an empty disc. Gentamicin was used as a positive control and DMSO as a solvent control. The Petri dishes were left to stand for 20 min at room temperature before incubation at 37 °C for 24 h. The experiment was performed in triplicate.

The broth microdilution assay was used to assess minimum bactericidal concentration (MBC) by the standard two-fold serial microdilution assay [68]. A suspension of test strains of microorganisms was prepared from daily cultures grown on slant agar at a temperature of 37 °C for 24 h. The microbial cultures were diluted in fresh Mueller–Hinton broth (MHB) to a final concentration of 10^6^ CFU/mL for bacteria. A suspension of microbes with a nutrient medium without a sample was placed in control tubes. The tested compounds and reference drug (Gentamicin) were diluted to obtain concentrations ranging from 50 to 1.56 μg/mL. The mixture was incubated in a thermostat for 24–48 h, depending on the class of the microorganism. Following this, upon visually determining the presence of turbidity in each of the tubes, the one that contained a clear suspension and the lowest concentration of the compound was chosen. This concentration was taken as the minimum bactericidal concentration. The experiment was performed in triplicate. Statistical processing was carried out by parametric statistical methods with the calculation of the arithmetic mean and standard error. Differences were considered significant at the achieved significance level of *p* < 0.05.

*Minimum inhibitory concentration (MIC) assay*. The MIC value was determined as the lowest concentration of the sample at which the tested microorganisms did not demonstrate any visible growth after incubation [68]. The antibacterial activity was compared with the effect of the drug ceftriaxone (Sintez, Kurgan, Russia). Dimethyl sulfoxide (99.55+% purity, Vekton, Russia) and commercial Mueller–Hinton agar/broth medium (BioMedia, St. Petersburg, Russia) were used to dissolve the test compounds. The bacteria were cultured in Mueller–Hinton agar/broth medium under anaerobic conditions at 37 °C. The cultivation time was 24 h for *S. aureus* and 48 h for *B. cereus*. The compounds were pre-dissolved in dimethyl sulfoxide (0.05 mL) and brought to the required concentration with a 0.9% NaCl solution and the nutrient broth. The seeding dose of the overnight bacterial cultures (for *S. aureus*, (6.09 ± 0.31) × 10^3^ colony-forming units (CFUs); for *B. cereus*, (7.38 ± 0.86) 10^3^ CFU) was determined using the McFarland standard and controlled by seeding onto a dense nutrient medium. The antibacterial activity was evaluated using serial twofold dilutions of the test compounds starting at 500 μg/mL. The minimum inhibitory concentration (MIC) was defined as the lowest concentration of the compound that completely inhibited the visible growth of bacteria. The absence of signs of growth in the liquid medium was controlled by seeding on agar medium followed by incubation under standard conditions. The following negative control was used. The test culture was added to the broth (1 mL), and the mixture was cultured under the same conditions, followed by seeding on an agar nutrient medium and the detection of bacterial growth. In this study, no bioactivity was defined as an MIC > 1000 µg/mL, mild bioactivity as an MIC in the range 512–1000 µg/mL, moderate bioactivity as an MIC in the range 128–512 µg/mL, good bioactivity as an MIC in the range 32–128 µg/mL, strong bioactivity as an MIC in the range 10–32 µg/mL, and very strong bioactivity as an MIC < 10 µg/mL. The mean MIC values and the standard deviation (M ± SEM) were calculated based on the results of repeated experiments. The statistical processing of the data was performed by a standard procedure using Student’s criterion.

*Modeling of biofilm formation was* performed on PVC fragments (the surface area was 1.0 ± 0.2 cm^2^) of intubation tubes (Alda-Healthcare, New Brunswick, NJ, USA). The tubes were fully immersed in liquid culture medium (1 mL) containing the broth (0.2 mL). Then, a seeding dose of microorganisms (0.1 mL of a suspension containing 3.5 × 10^6^ cells/mL of an overnight culture of the strain) was added [69]. After 24 h of incubation, the fragment of the tube was withdrawn from the culture medium, washed with a 0.9% sodium chloride solution, and placed in a 0.02% ethylenediaminetetraacetic acid solution (1 mL) for 5 min at 37 °C to detach the bacterial cells. The number of microbial cells was evaluated by the subsequent seeding on dense culture medium, followed by incubation for 24 h and the calculation of CFUs. The experiment was performed in triplicate.

### 3.3. Molecular Docking

The three-dimensional structures of quinine (compound **1**) and its derivatives (compounds **4d**, **11**, **12**, **23**, **26**, **27**, **30**) were constructed using the ChemOffice 2016 software (PerkinElmer Informatics, Waltham, MA, USA) and subsequently energy-minimized by employing the MM2 force field to refine their geometries. The crystal structure of UDP-N-acetylenolpyruvylglucosamine reductase (MurB) from Staphylococcus aureus, co-crystallized with flavin adenine dinucleotide (FAD) [70], was retrieved from the Protein Data Bank (PDB ID: 1HSK).

Molecular docking was conducted using Molegro Virtual Docker (MVD) version 6.0 (CLC bio, Qiagen, Aarhus, Denmark). The protein structure and ligand models were imported into the MVD workspace. To define the search space, a spherical area with a radius of 12 Å was placed on the centroid of the co-crystallized FAD molecule, encompassing the FAD binding site. Given the high conformational flexibility of the ligands and the elongated topology of the binding pocket, template-based docking was implemented, utilizing the FAD cofactor as the structural template to guide pose generation.

For each ligand, 500 independent docking runs were executed with the “Internal HBond” and “Sp^2^-Sp^2^ Torsions” ligand evaluation options enabled to account for possible intramolecular hydrogen bonding and torsional flexibility in π-electron systems. The MolDock scoring function [65] was applied to evaluate the docked poses, providing docking scores (DS) that quantify the overall interaction energy between the ligand and the protein in units of the MolDock force field. Key interacting residues were identified based on partial MolDock scores more negative than −10 units, and hydrogen bonds were analyzed within the top-scoring poses. Ligand moiety placements were characterized relative to the structural fragments of the co-crystallized FAD (adenine, ribose, pyrophosphate, ribitol, and isoalloxazine).

## 4. Conclusions

We developed an efficient strategy for the modification of quinine with the introduction of a (((4-X-but-2-ynyloxy)methyl)triazolyl)-substituent at the C-9 position. These compounds can be easily prepared through straightforward procedures with commercially available starting materials and do not require additional resolution. Their antibacterial activity was further evaluated against *Staphylococcus aureus*, *Bacillus subtilis*, *Bacillus cereus*, and *Escherichia coli*. After introducing *S. aureus* culture at a dose of (6.09 ± 0.31) × 10^3^ CFU, compound **26** completely suppressed bacterial growth in a concentration of 181 ± 12.8 µg/mL, and diastereomeric compound **30** possessed a minimum inhibitory concentration value equal to 179.2 ± 18.4 µg/mL. Furthermore, compounds **26** and **30** prevented biofilm formation by the bacterial strain *S. aureus* with an MIC of 107.4 ± 7.7 µg/mL and 82.4 ± 8.7 µg/mL. Molecular docking of the studied compounds was carried out based on the ChemOffice 2016 software (PerkinElmer Informatics, Waltham, MA, USA), and the results are well corroborated by the found antibacterial activity. The obtained results regarding the structure–activity relationships are quite important for the further design of new C-9 (but-2-ynyloxy)triazolyl- and (propynyloxy)triazolyl-substituted 9-desoxycinchona alkaloid derivatives.

## Figures and Tables

**Table 1:** Antibacterial activity of 9-triazolylsubstituted derivatives of *epi*-quinine **4a–d** and **8** against bacterial strains determined by disc diffusion assay and broth microdilution assay.

Compound	Diameters of the Inhibition Zone (mm) ^[a]^			Minimum Bactericidal Concentration, MBC (μg/mL)		
	*Staphylococcus aureus* *ATCC 6538*-P	*Bacillus subtilis* *ATCC 6633*	*Escherichia coli* *ATCC 25922*	*Staphylococcus aureus* *ATCC 6538*-P	*Bacillus subtilis* *ATCC 6633*	*Escherichia coli* *ATCC 25922*
**4a**	12 ± 0.2	15 ± 0.1 *	15 ± 0.1 *		25	25
**4b**	14 ± 0.1 *	13 ± 0.2	16 ± 0.2	25		12.5
**4c**	-	-	14 ± 0.2			50
**4d**	13 ± 0.2	-	14 ± 0.1			50
**8**	15 ± 0.2	-	15 ± 0.1 *	25		25
Gentamicin	24 ± 0.1	21 ± 0.2	26 ± 0.1	11.8	22.5	11.3

^[a]^ Diameter of inhibition zone (mm) around the disc; * *p* < 0.05 vs. Gentamicin; the symbol “-” means that the compound was not active in this experiment; all values are expressed as mean ± SD of three parallel measurements.

**Table 2:** The in vitro antibacterial activity of the new type of cinchona alkaloid derivatives, **11**, **12**, **14**, **15**, **17**, **18**, and **23**–**30**, on bacterial strains via a serial dilution assay in a liquid nutrient medium.

Compound	MIC, μg/mL ^[a,b]^		Compound	MIC, μg/mL ^[a]^	
	*S. aureus* 209 ATCC 6538-P ^[b]^	*B. cereus* ATCC 10702 ^[c]^		*S. aureus* 209 ATCC 6538-P ^[b]^	*B. cereus* ATCC 10702 ^[c]^
**11**	>1000	596.5 ± 3.2	**24**	385 ± 14.3	366.7 ± 33.3
**12**	>1000	456.7 ± 29.6	**28**	250 ± 26.7	291.7 ± 8.3
**14**	>1000	481.7 ± 18.3	**25**	350 ± 34.3	296.5 ± 23.2
**15**	>1000	750 ± 76.4	**29**	200 ± 16.4	283.2 ± 28.8
**17**	646.7 ± 33.3	533.3 ± 33.3	**26**	181 ± 12.8	218.8 ± 18.75
**18**	683.3 ± 92.8	633.3 ± 66.7	**30**	179.2 ± 18.4	107.5 ± 10.9
**23**	416.7 ± 22.6	498.3 ± 30.3	Quinine 1	>1000	956.7 ± 109.6
**27**	303.3 ± 43.3	545.8 ± 26.7	Ceftriaxonum	2.7 ± 0.7	6.03 ± 0.52

^[a]^ MIC: minimum inhibition concentrations. ^[b]^ The culture of *S. aureus* was introduced in an amount equal to (6.09 ± 0.31) × 10^3^ CFU; ^[c]^ the culture of *B. cereus* was introduced in an amount equal to (7.38 ± 0.86) × 10^3^ CFU.

**Table 3:** The effect of compound **26** on inhibition of *S. aureus* 209 ATCC 6538-P growth in a liquid medium and film formation (MIC = 181 ± 12.8 µg/mL).

Doses (M ± m), µg/mL	250	125	62.5	31.25	Culture Control
Growth in the broth, CFU/mL	0	(1.74 ± 0.57) × 10^4^ *	(1.31 ± 0.27) × 10^5^ *	(9.6 ± 0.25) × 10^6^ *	(1.38 ± 0.12) × 10^7^
biofilm formation, CFU/mL	0	0	(5.0 ± 1.83) × 10^2^ *	(9.2 ± 0.61) × 10^5^ *	(2.9 ± 0.2) × 10^6^

* Statistically significant differences compared to control (*p* < 0.05).

**Table 4:** The effect of compound **30** on inhibition of *S. aureus* 209 ATCC 6538-P growth in a liquid medium and film formation (MIC = 179.2 ± 18.4 µg/mL).

Doses (M ± m), µg/mL	250	125	62.5	31.25	Culture Control
Growth in the broth, CFU/mL	0	(5.0 ± 1.39) × 10^2^ *	(4.18 ± 0.57) × 10^4^ *	(1.03 ± 0.14) × 10^5^ *	(1.38 ± 0.12) × 10^7^
biofilm formation, CFU/mL	0	0	(1.08 ± 0.83) × 10^2^	(3.38 ± 0.31) × 10^5^ *	(2.9 ± 0.2) × 10^6^

* Statistically significant differences compared to control (*p* < 0.05).

**Table 5:** The MolDock docking scores and key interactions found during docking of the investigated compounds to the FAD binding site of MurB (PDB: 1HSK).

Com-pound	DS *	Interacting Residues **	Hydrogen Bonds (Ligand Group)	Location of The Ligand Moieties ***
**1**	−123.5	Tyr77, Tyr149, Val199, Gly145, Gly146, Leu78	Tyr77 (OH), Ser143 (OMe), Val199 (quinoline nitrogen)	Quinoline on adenine, quinuclidine on ribose, vinyl on pyrophosphate
**4d**	−173.6	Tyr149, Tyr77, Gly146, Leu78, Ser143, Gly145, Arg310, Gly79, Asn83	Ser143 (OMe), Tyr149 (Me_2_CHOH), Arg310 (triazole N-3, weak)	Quinoline on adenine, quinuclidine and vinyl on pyrophosphate, triazole on ribose
**11**	−185.7	Tyr149, Gly146, Tyr77, Arg310, Asn83, Leu78, Gly145, Ser143, Gly79, Ile84	Ser143 (OMe), Tyr149 (propargyloxy), Arg310 (triazole N-3, weak; propargyloxy, weak)	Quinoline on adenine, quinuclidine and vinyl on pyrophosphate, triazole on ribose, propargyloxy apart from FAD
**12**	−191.0	Tyr149, Tyr77, Leu78, Gly146, Gly145, Ser143, Asn83	Ser82 (propargyloxy), Ser143 (triazole N-3)	Quinoline on adenine, quinuclidine on ribose, triazole near pyrophosphate, propargyloxy on ribitol
**23**	−199.2	Tyr77, Tyr149, Leu78, Ser82, Gly146, Gly145, Gly79, Asn83, Pro141	Gly79 (triazole N-2; triazole N-3, weak), Gly81 (triazole N-3), Ser82 (propargyloxy)	Quinoline on adenine, quinuclidine on ribose, triazole near pyrophosphate, propargyloxy on ribitol, pyrrolidine near isoalloxazine
**27**	−216.3	Tyr77, Tyr149, Ser82, Asn83, Leu78, Gly145, Pro141, Gly146, Ser143	Ser82 (propargyloxy), Ser143 (triazole N-3)	Quinoline on adenine, quinuclidine on ribose, triazole near pyrophosphate, propargyloxy on ribitol, pyrrolidine near isoalloxazine
**26**	−207.7	Tyr77, Tyr149, Leu78, Ser82, Gly146, Pro141, Gly145, Gly79	Gly79 (triazole N-2, N-3), Gly81 (triazole N-3), Ser82 (propargyloxy)	Quinoline on adenine, quinuclidine on ribose, triazole near pyrophosphate, propargyloxy on ribitol, azocane on isoalloxazine
**30**	−226.4	Tyr77, Tyr149, Asn83, Ser82, Leu78, Gly145, Pro141, Gly146, Ser143	Ser82 (propargyloxy), Ser143 (triazole N-3)	Quinoline on adenine, quinuclidine on ribose, triazole near pyrophosphate, propargyloxy on ribitol, azocane on isoalloxazine
**FAD**	−315.5	Ser82, Pro141, Tyr149, Tyr77, Ile140, Val199, Gly146, Arg225, Asn83, Asn80, Ser143, Leu78, Ile84, Gly79, Gly145	Tyr77 (ribose OH), Gly79(pyrophosphate), Asn80(pyrophosphate), Gly81 (pyrophosphate), Ser82 (pyrophosphate; ribitol OH), Asn83 (pyrophpsphate), Pro141 (ribitol OH), Ser143(pyrophosphate), Tyr149 (ribose OH, weak), Gly153 (isoalloxazine N-3; isoalloxazine carbonyl at C-2), Val199 (adenine NH_2_; adenine N-1), Arg225 (isoalloxazine N-5; isoalloxazine carbonyl at C-4)	-

* Units of the MolDock force field. ** Residues exhibiting partial MolDock scores below −10 units are ranked in descending order of negativity (i.e., from the most negative partial score). *** The positioning with respect to the co-crystallized FAD cofactor in the 1HSK structure.

## Data Availability

The data are contained within the article.

## Supplementary Materials

The following supporting information can be downloaded at: https://www.mdpi.com/article/10.3390/molecules30224352/s1. Figure S1: 1H NMR spectrum of (2R,4S,5S)-2-((S)-(4-(4-methoxyphenyl)-1H-1,2,3-triazol-1-yl)(6-methoxyquinolin-4-yl)methyl)-5-vinylquinuclidine (**4a**) (CDCl3, 300 MHz). Figure S2: 13C NMR spectrum of (2R,4S,5S)-2-((S)-(4-(4-methoxyphenyl)-1H-1,2,3-triazol-1-yl)(6-methoxyquinolin-4-yl)methyl)-5-vinylquinuclidine (**4a**) (CDCl3, 75 MHz). Figure S3: 1H NMR spectrum of (2R,4S,5S)-2-((S)-(4-(4-fluorophenyl)-1H-1,2,3-triazol-1-yl)(6-methoxyquinolin-4-yl)methyl)-5-vinylquinuclidine (**4b**) (CDCl3, 400 MHz). Figure S4: 13C NMR spectrum of (2R,4S,5S)-2-((S)-(4-(4-fluorophenyl)-1H-1,2,3-triazol-1-yl)(6-methoxyquinolin-4-yl)methyl)-5-vinylquinuclidine (**4b**) (CDCl3, 75 MHz). Figure S5: 1H NMR spectrum of (2R,4S,5S)-2-((S)-(6-methoxyquinolin-4-yl)(4-p-tolyl-1H-1,2,3-triazol-1-yl)methyl)-5-vinylquinuclidine (**4c**) (CDCl3, 400 MHz). Figure S6: 13C NMR spectrum of (2R,4S,5S)-2-((S)-(6-methoxyquinolin-4-yl)(4-p-tolyl-1H-1,2,3-triazol-1-yl)methyl)-5-vinylquinuclidine (**4c**) (CDCl3, 75 MHz). Figure S7: 1H NMR spectrum of (1-((S)-(6-methoxyquinolin-4-yl)((2R,4S,5R)-5-vinylquinuclidin-2-yl)methyl)-1H-1,2,3-triazol-4-yl)methanol (**4d**) (CDCl3, 500 MHz). Figure S8: 13C NMR spectrum of (1-((S)-(6-methoxyquinolin-4-yl)((2R,4S,5R)-5-vinylquinuclidin-2-yl)methyl)-1H-1,2,3-triazol-4-yl)methanol (**4d**) (CDCl3, 100 MHz). Figure S9: 1H NMR spectrum of 2-(1-((S)-(6-methoxyquinolin-4-yl)((2S,4S,5R)-5-vinylquinuclidin-2-yl)methyl)-1H-1,2,3-triazol-4-yl)propan-2-ol (**4e**) (CDCl3, 400 MHz). Figure S10: 13C NMR spectrum of 2-(1-((S)-(6-methoxyquinolin-4-yl)((2S,4S,5R)-5-vinylquinuclidin-2-yl)methyl)-1H-1,2,3-triazol-4-yl)propan-2-ol (**4e**) (CDCl3, 126 MHz). Figure S11: 1H NMR spectrum of 3-((1-((S)-(6-methoxyquinolin-4-yl)((2R,4S,5R)-5-vinylquinuclidin-2-yl)methyl)-1H-1,2,3-triazol-4-yl)methylthio)-1H-1,2,4-triazol-5-amine (**8**) (CD3)2SO, 300 MHz). Figure S12: 13C NMR spectrum of 3-((1-((S)-(6-methoxyquinolin-4-yl)((2R,4S,5R)-5-vinylquinuclidin-2-yl)methyl)-1H-1,2,3-triazol-4-yl)methylthio)-1H-1,2,4-triazol-5-amine (**8**) (CD3)2SO, 75 MHz). Figure S13: 1H NMR of (1-((S)-(6-methoxyquinolin-4-yl)((2R,4S,5R)-5-vinylquinuclidin-2-yl)-methyl)-1H-1,2,3-triazol-4-yl)methyl 3-tert-butyl-5-ethyl-2-hydroxybenzoate (**9**) (CDCl3, 300MHz). Figure S14: 13C NMR of (1-((S)-(6-methoxyquinolin-4-yl)((2R,4S,5R)-5-vinylquinuclidin-2-yl)-methyl)-1H-1,2,3-triazol-4-yl)methyl 3-tert-butyl-5-ethyl-2-hydroxybenzoate (**9**) (CDCl3, 75 MHz). Figure S15: 1H NMR spectrum of (2R,4S,5R)-2-((S)-(6-methoxyquinolin-4-yl)(4-((prop-2-ynyloxy)methyl)-1H-1,2,3-triazol-1-yl)methyl)-5-vinylquinuclidine (**11**) (CDCl3, 300 MHz). Figure S16: 13C NMR spectrum of(2R,4S,5R)-2-((S)-(6-methoxyquinolin-4-yl)(4-((prop-2-ynyloxy)methyl)-1H-1,2,3-triazol-1-yl)methyl)-5-vinylquinuclidine (**11**) (CDCl3, 100 MHz). Figure S17: 1H NMR spectrum of (2R,4S,5R)-2-((R)-(6-methoxyquinolin-4-yl)(4-((prop-2-ynyloxy)methyl)-1H-1,2,3-triazol-1-yl)methyl)-5-vinylquinuclidine (**12**) (CDCl3, 400 MHz). Figure S18: 13C NMR spectrum of (2R,4S,5R)-2-((R)-(6-methoxyquinolin-4-yl)(4-((prop-2-ynyloxy)methyl)-1H-1,2,3-triazol-1-yl)methyl)-5-vinylquinuclidine (**12**) (CDCl3, 300 MHz). Figure S19: 1H NMR of 4-((1-((S)-(6-methoxyquinolin-4-yl)((2R,4S,5R)-5-vinylquinuclidin-2-yl)methyl)-1H-1,2,3-triazol-4-yl)methoxy)-N,N-dipropylbut-2-yn-1-amine (**14**) (CDCl3, 300 MHz). Figure S20: 13C NMR of 4-((1-((S)-(6-methoxyquinolin-4-yl)((2R,4S,5R)-5-vinylquinuclidin-2-yl)methyl)-1H-1,2,3-triazol-4-yl)methoxy)-N,N-dipropylbut-2-yn-1-amine (**14**) (CDCl3, 126 MHz). Figure S21: 1H NMR of 4-((1-((R)-(6-methoxyquinolin-4-yl)((2R,4S,5R)-5-vinylquinuclidin-2-yl)methyl)-1H-1,2,3-triazol-4-yl)methoxy)-N,N-dipropylbut-2-yn-1-amine (**15**) (CDCl3, 400 MHz). Figure S22: 13C NMR of 4-((1-((R)-(6-methoxyquinolin-4-yl)((2R,4S,5R)-5-vinylquinuclidin-2-yl)methyl)-1H-1,2,3-triazol-4-yl)methoxy)-N,N-dipropylbut-2-yn-1-amine (**15**) (CDCl3, 126 MHz). Figure S23: 1H NMR of 4-((1-((S)-(6-methoxyquinolin-4-yl)((2R,4S,5R)-5-vinylquinuclidin-2-yl)methyl)-1H-1,2,3-triazol-4-yl)methoxy)-N,N-diisopropylbut-2-yn-1-amine (**17**) (CDCl3, 500 MHz). Figure S24: 13C NMR of 4-((1-((S)-(6-methoxyquinolin-4-yl)((2R,4S,5R)-5-vinylquinuclidin-2-yl)methyl)-1H-1,2,3-triazol-4-yl)methoxy)-N,N-diisopropylbut-2-yn-1-amine (**17**) (CDCl3, 75 MHz). Figure S25: 1H NMR of 4-((1-((R)-(6-methoxyquinolin-4-yl)((2R,4S,5R)-5-vinylquinuclidin-2-yl)methyl)-1H-1,2,3-triazol-4-yl)methoxy)-N,N-diisopropylbut-2-yn-1-amine (**18**) (CDCl3, 400 MHz). Figure S26: 13C NMR of 4-((1-((R)-(6-methoxyquinolin-4-yl)((2R,4S,5R)-5-vinylquinuclidin-2-yl)methyl)-1H-1,2,3-triazol-4-yl)methoxy)-N,N-diisopropylbut-2-yn-1-amine (**18**) (CDCl3, 126 MHz). Figure S27: 1H NMR of (2R,4S,5R)-2-((S)-(6-methoxyquinolin-4-yl)(4-((4-(pyrrolidin-1-yl)but-2-ynyloxy)methyl)-1H-1,2,3-triazol-1-yl)methyl)-5-vinylquinuclidine (**23**) (CDCl3, 400 MHz). Figure S28: 13C NMR of (2R,4S,5R)-2-((S)-(6-methoxyquinolin-4-yl)(4-((4-(pyrrolidin-1-yl)but-2-ynyloxy)methyl)-1H-1,2,3-triazol-1-yl)methyl)-5-vinylquinuclidine (**23**) (CDCl3, 101 MHz). Figure S29: 1H NMR of (2R,4S,5R)-2-((R)-(6-methoxyquinolin-4-yl)(4-((4-(pyrrolidin-1-yl)but-2-ynyloxy)methyl)-1H-1,2,3-triazol-1-yl)methyl)-5-vinylquinuclidine (**27**) (CDCl3, 400 MHz). Figure S30: 13C NMR of (2R,4S,5R)-2-((R)-(6-methoxyquinolin-4-yl)(4-((4-(pyrrolidin-1-yl)but-2-ynyloxy)methyl)-1H-1,2,3-triazol-1-yl)methyl)-5-vinylquinuclidine (**27**) (CDCl3, 125 MHz). Figure S31: 1H NMR of (2R,4S,5R)-2-((S)-(6-methoxyquinolin-4-yl)(4-((4-(piperidin-1-yl)but-2-ynyloxy)methyl)-1H-1,2,3-triazol-1-yl)methyl)-5-vinylquinuclidine (**24**) (CDCl3, 300 MHz). Figure S32: 13C NMR of (2R,4S,5R)-2-((S)-(6-methoxyquinolin-4-yl)(4-((4-(piperidin-1-yl)but-2-ynyloxy)methyl)-1H-1,2,3-triazol-1-yl)methyl)-5-vinylquinuclidine (**24**) (CDCl3, 126 MHz). Figure S33: 1H NMR of (2R,4S,5R)-2-((R)-(6-methoxyquinolin-4-yl)(4-((4-(piperidin-1-yl)but-2-ynyloxy)methyl)-1H-1,2,3-triazol-1-yl)methyl)-5-vinylquinuclidine (**28**) (CDCl3, 300 MHz). Figure S34: 13C NMR of (2R,4S,5R)-2-((R)-(6-methoxyquinolin-4-yl)(4-((4-(piperidin-1-yl)but-2-ynyloxy)methyl)-1H-1,2,3-triazol-1-yl)methyl)-5-vinylquinuclidine (**28**) (CDCl3, 126 MHz). Figure S35: 1H NMR spectrum of (2R,4S,5R)-2-((S)-(4-((4-(azepan-1-yl)but-2-ynyloxy)methyl)-1H-1,2,3-triazol-1-yl)(6-methoxyquinolin-4-yl)methyl)-5-vinylquinuclidine (**25**) (CDCl3, 300 MHz). Figure S36: 13C NMR spectrum of (2R,4S,5R)-2-((S)-(4-((4-(azepan-1-yl)but-2-ynyloxy)methyl)-1H-1,2,3-triazol-1-yl)(6-methoxyquinolin-4-yl)methyl)-5-vinylquinuclidine (**25**) (CDCl3, 101 MHz). Figure S37: 1H NMR spectrum of (2R,4S,5R)-2-((R)-(4-((4-(azepan-1-yl)but-2-ynyloxy)methyl)-1H-1,2,3-triazol-1-yl)(6-methoxyquinolin-4-yl)methyl)-5-vinylquinuclidine (**29**) (CDCl3, 300 MHz). Figure S38: 13C NMR spectrum of (2R,4S,5R)-2-((R)-(4-((4-(azepan-1-yl)but-2-ynyloxy)methyl)-1H-1,2,3-triazol-1-yl)(6-methoxyquinolin-4-yl)methyl)-5-vinylquinuclidine (**29**) (CDCl3, 126 MHz). Figure S39: 1H NMR spectrum of (2R,4S,5R)-2-((S)-(4-((4-(azocan-1-yl)but-2-ynyloxy)methyl)-1H-1,2,3-triazol-1-yl)(6-methoxyquinolin-4-yl)methyl)-5-vinylquinuclidine (**26**) (CDCl3, 400 MHz). Figure S40: 13C NMR spectrum of (2R,4S,5R)-2-((S)-(4-((4-(azocan-1-yl)but-2-ynyloxy)methyl)-1H-1,2,3-triazol-1-yl)(6-methoxyquinolin-4-yl)methyl)-5-vinylquinuclidine (**26**) (CDCl3, 75 MHz). Figure S41: 1H NMR spectrum of (2R,4S,5R)-2-((R)-(4-((4-(azocan-1-yl)but-2-ynyloxy)methyl)-1H-1,2,3-triazol-1-yl)(6-methoxyquinolin-4-yl)methyl)-5-vinylquinuclidine (**30**) (CDCl3, 400 MHz). Figure S42: 13C NMR spectrum of (2R,4S,5R)-2-((R)-(4-((4-(azocan-1-yl)but-2-ynyloxy)methyl)-1H-1,2,3-triazol-1-yl)(6-methoxyquinolin-4-yl)methyl)-5-vinylquinuclidine (**30**) (CDCl3, 100 MHz).
