# Supplementary material for: Synthesis, Antibacterial Properties and Molecular Docking Studies of Nitrogen Substituted 9-(((4X-But-2-ynyloxy)methyl)-1,2,3-triazolyl)–Cinchona Alkaloid Conjugates
Source: Molecules. 2025 Nov 10;30(22):4352. doi: 10.3390/molecules30224352 (PMC12654649; doi:10.3390/molecules30224352)
Supplement: Supplementary file 1 [file molecules-30-04352-s001.zip › molecules-3957067-supplementary.pdf]

SUPPLEMENTARY MATERIAL

Synthesis, Antibacterial Properties and Molecular Docking Studies of Nitrogen Substituted 9-(((4X-But-2-ynyloxy)methyl)-1,2,3-triazolyl)–Cinchona Alkaloid Conjugates

Gulim K. Mukusheva [1,*], Nurizat N. Toigambekova [1], Victor A. Savelyev [2], Andrey I. Khlebnikov [3], Liubov G. Burova [4], Sofiia D. Afanaseva [4], Oralgazy A. Nurkenov [5], Anarkul S. Kishkentayeva [6], Aikerim S. Olzhabayeva [1], Yurii V. Gatilov [2], Roza B. Seidakhmetova [7], Alexander N. Evstropov [4] and Elvira E. Shults [2,*]

[1] Chemistry Faculty, Karaganda National Research University Buketov, Karaganda 100024, Kazakhstan; nukonti92@mail.ru (N.N.T.); aikerim.olzhabayeva@mail.ru (A.S.O.)
[2] Vorozhtsov Novosibirsk Institute of Organic Chemistry, Siberian Branch of the Russian Academy of Sciences, Novosibirsk 630090, Russia; vicsav@nioch.nsc.ru (V.A.S.); gatilov@nioch.nsc.ru (Y.V.G.)
[3] Kizhner Research Center, Tomsk Polytechnic University, Tomsk 634050, Russia; aikhl@chem.org.ru
[4] Department of Microbiology, Immunology and Virology, Novosibirsk State Medical University, Krasny Prospect 52, Novosibirsk 630091, Russia; mic.bio.lgb@mail.ru (L.G.B.); sofylennon@mail.ru (S.D.A.); microbiongmu@yandex.ru (A.N.E.)
[5] Institute of Organic Synthesis and Coal Chemistry of the Republic of Kazakhstan, Karaganda 100008, Kazakhstan; nurkenov_oral@mail.ru
[6] School of Pharmacy, Medical University of Karaganda, Karaganda 100012, Kazakhstan; anar_kish@mail.ru
[7] Department of Clinical Pharmacology and Evidence-Based Medicine, Karaganda Medical University, Karaganda 100012, Kazakhstan; roza77124@gmail.com
* Correspondence: mukushevagulim5@gmail.com (G.K.M.); schultz@nioch.nsc.ru (E.E.S.)

Table of contents

1 Figure S1. ^{1}H NMR spectrum of (2*R*,4*S*,5*S*)-2-((*S*)-(4-(4-methoxyphenyl)-1*H*-1,2,3-triazol-1-yl)(6-methoxyquinolin-4-yl)methyl*)*-5-vinylquinuclidine **(4a)** ($CDCl_3$, 300 MHz). 3

2 Figure S2. ^{13}C NMR spectrum of (2*R*,4*S*,5*S*)-2-((*S*)-(4-(4-methoxyphenyl)-1*H*-1,2,3-triazol-1-yl)(6-methoxyquinolin-4-yl)methyl*)*-5-vinylquinuclidine **(4a)** ($CDCl_3$, 75 MHz). 4

3 Figure S3. ^{1}H NMR spectrum of (2*R*,4*S*,5*S*)-2-((*S*)-(4-(4-fluorophenyl)-1*H*-1,2,3-triazol-1-yl)(6-methoxyquinolin-4-yl)methyl*)*-5-vinylquinuclidine (**4b**) ($CDCl_3$, 400 MHz). 5

4 Figure S4. ^{13}C NMR spectrum of (2*R*,4*S*,5*S*)-2-((*S*)-(4-(4-fluorophenyl)-1*H*-1,2,3-triazol-1-yl)(6-methoxyquinolin-4-yl)methyl*)*-5-vinylquinuclidine (**4b**) ($CDCl_3$, 75 MHz). 6

5 Figure S5. ^{1}H NMR spectrum of (2*R*,4*S*,5*S*)-2-((*S*)-(6-methoxyquinolin-4-yl)(4-*p*-tolyl-1*H*-1,2,3-triazol-1-yl)methyl)-5-vinylquinuclidine **(4c)** ($CDCl_3$, 400 MHz). 7

6 Figure S6. ^{13}C NMR spectrum of (2*R*,4*S*,5*S*)-2-((*S*)-(6-methoxyquinolin-4-yl)(4-*p*-tolyl-1*H*-1,2,3-triazol-1-yl)methyl)-5-vinylquinuclidine **(4c)** ($CDCl_3$, 75 MHz). 8

7 Figure S7. ^{1}H NMR spectrum of (1-((*S*)-(6-methoxyquinolin-4-yl)((2*R*,4*S*,5*R*)-5-vinylquinuclidin-2-yl)methyl)-1H-1,2,3-triazol-4-yl)methanol (**4d**) ($CDCl_3$, 500 MHz). 9

8 Figure S8. ^{13}C NMR spectrum of (1-((*S*)-(6-methoxyquinolin-4-yl)((2*R*,4*S*,5*R)*-5-vinylquinuclidin-2-yl)methyl)-1H-1,2,3-triazol-4-yl)methanol (**4d**) ($CDCl_3$, 100 MHz).. 10

9 Figure S9. ^{1}H NMR spectrum of 2-(1-((*S*)-(6-methoxyquinolin-4-yl)((2*S*,4*S*,5*R*)-5-vinylquinuclidin-2-yl)methyl)-1H-1,2,3-triazol-4-yl)propan-2-ol (**4e**) ($CDCl_3$, 400 MHz). 11

10 Figure S10. ^{13}C NMR spectrum of 2-(1-((S)-(6-methoxyquinolin-4-yl)((2*S*,4*S*,5*R)*-5-vinylquinuclidin-2-yl)methyl)-1*H*-1,2,3-triazol-4-yl)propan-2-ol (**4e**) ($CDCl_3$, 126 MHz). 12

11 Figure S11. ^{1}H NMR spectrum of 3-((1-((*S*)-(6-methoxyquinolin-4-yl)((2*R*,4*S*,5*R*)-5-vinylquinuclidin-2-yl)methyl)-1*H*-1,2,3-triazol-4-yl)methylthio)-1*H*-1,2,4-triazol-5-amine (**8**) $(CD_3)_2SO$, 300 MHz). 13

12 Figure S12. ^{13}C NMR spectrum of 3-((1-((*S*)-(6-methoxyquinolin-4-yl)((2*R*,4*S*,5*R*)-5-vinylquinuclidin-2-yl)methyl)-1*H*-1,2,3-triazol-4-yl)methylthio)-1*H*-1,2,4-triazol-5-amine (**8**) $(CD_3)_2SO$, 75 MHz). 14

Figure S13. ^{1}H NMR of (1-((*S*)-(6-methoxyquinolin-4-yl)((2*R*,4*S*,5*R*)-5-vinylquinuclidin-2-yl)-methyl)-1H-1,2,3-triazol-4-yl)methyl 3-tert-butyl-5-ethyl-2-hydroxybenzoate (**9**) ($CDCl_3$, 300MHz). 15
Figure S14. ^{13}C NMR of (1-((*S*)-(6-methoxyquinolin-4-yl)((2*R*,4*S*,5*R*)-5-vinylquinuclidin-2-yl)-methyl)-1H-1,2,3-triazol-4-yl)methyl 3-tert-butyl-5-ethyl-2-hydroxybenzoate (**9**) ($CDCl_3$, 75 MHz). 16
Figure S15. ^{1}H NMR spectrum of (2*R*,4*S*,5*R*)-2-((*S*)-(6-methoxyquinolin-4-yl)(4-((prop-2-ynyloxy)methyl)-1*H*-1,2,3-triazol-1-yl)methyl)-5-vinylquinuclidine (**11**) ($CDCl_3$, 300 MHz). 17
Figure S16. ^{13}C NMR spectrum of(2*R*,4*S*,5*R*)-2-((*S*)-(6-methoxyquinolin-4-yl)(4-((prop-2-ynyloxy)methyl)-1*H*-1,2,3-triazol-1-yl)methyl)-5-vinylquinuclidine (**11**) ($CDCl_3$, 100 MHz). 18
Figure S17. ^{1}H NMR spectrum of (2*R*,4*S*,5*R*)-2-((*R*)-(6-methoxyquinolin-4-yl)(4-((prop-2-ynyloxy)methyl)-1*H*-1,2,3-triazol-1-yl)methyl)-5-vinylquinuclidine (**12**) ($CDCl_3$, 400 MHz). 19
Figure S18. ^{13}C NMR spectrum of (2*R*,4*S*,5*R*)-2-((*R*)-(6-methoxyquinolin-4-yl)(4-((prop-2-ynyloxy)methyl)-1*H*-1,2,3-triazol-1-yl)methyl)-5-vinylquinuclidine (**12**) ($CDCl_3$, 300 MHz). 20
Figure S19. ^{1}H NMR of 4-((1-((*S*)-(6-methoxyquinolin-4-yl)((2*R*,4*S*,5*R*)-5-vinylquinuclidin-2-yl)methyl)-1*H*-1,2,3-triazol-4-yl)methoxy)-N,N-dipropylbut-2-yn-1-amine (**14**) ($CDCl_3$, 300 MHz). 21
Figure S20. ^{13}C NMR of 4-((1-((*S*)-(6-methoxyquinolin-4-yl)((2*R*,4*S*,5*R*)-5-vinylquinuclidin-2-yl)methyl)-1*H*-1,2,3-triazol-4-yl)methoxy)-N,N-dipropylbut-2-yn-1-amine (**14**) ($CDCl_3$, 126 MHz). 22
Figure S21. ^{1}H NMR of 4-((1-((*R*)-(6-methoxyquinolin-4-yl)((2*R*,4*S*,5*R*)-5-vinylquinuclidin-2-yl)methyl)-1*H*-1,2,3-triazol-4-yl)methoxy)-N,N-dipropylbut-2-yn-1-amine (**15**) ($CDCl_3$, 400 MHz). 23
Figure S22. ^{13}C NMR of 4-((1-((*R*)-(6-methoxyquinolin-4-yl)((2*R*,4*S*,5*R*)-5-vinylquinuclidin-2-yl)methyl)-1*H*-1,2,3-triazol-4-yl)methoxy)-N,N-dipropylbut-2-yn-1-amine (**15**) ($CDCl_3$, 126 MHz). 24
Figure S23. ^{1}H NMR of 4-((1-((*S*)-(6-methoxyquinolin-4-yl)((2*R*,4*S*,5*R*)-5-vinylquinuclidin-2-yl)methyl)-1*H*-1,2,3-triazol-4-yl)methoxy)-N,N-diisopropylbut-2-yn-1-amine (**17**) ($CDCl_3$, 500 MHz). 25
Figure S24. ^{13}C NMR of 4-((1-((*S*)-(6-methoxyquinolin-4-yl)((2*R*,4*S*,5*R*)-5-vinylquinuclidin-2-yl)methyl)-1*H*-1,2,3-triazol-4-yl)methoxy)-N,N-diisopropylbut-2-yn-1-amine (**17**) ($CDCl_3$, 75 MHz). 26
Figure S25. ^{1}H NMR of 4-((1-((*R*)-(6-methoxyquinolin-4-yl)((2*R*,4*S*,5*R*)-5-vinylquinuclidin-2-yl)methyl)-1*H*-1,2,3-triazol-4-yl)methoxy)-N,N-diisopropylbut-2-yn-1-amine (**18**) ($CDCl_3$, 400 MHz). 27
Figure S26. ^{13}C NMR of 4-((1-((*R*)-(6-methoxyquinolin-4-yl)((2*R*,4*S*,5*R*)-5-vinylquinuclidin-2-yl)methyl)-1*H*-1,2,3-triazol-4-yl)methoxy)-N,N-diisopropylbut-2-yn-1-amine (**18**) ($CDCl_3$, 126 MHz). 28
Figure S27. ^{1}H NMR of (2*R*,4*S*,5*R*)-2-((*S*)-(6-methoxyquinolin-4-yl)(4-((4-(pyrrolidin-1-yl)but-2-ynyloxy)methyl)-1*H*-1,2,3-triazol-1-yl)methyl)-5-vinylquinuclidine (**23**) ($CDCl_3$, 400 MHz). 29
Figure S28. ^{13}C NMR of (2*R*,4*S*,5*R*)-2-((*S*)-(6-methoxyquinolin-4-yl)(4-((4-(pyrrolidin-1-yl)but-2-ynyloxy)methyl)-1*H*-1,2,3-triazol-1-yl)methyl)-5-vinylquinuclidine (**23**) ($CDCl_3$, 101 MHz). 30
Figure S29. ^{1}H NMR of (2*R*,4*S*,5*R*)-2-((*R*)-(6-methoxyquinolin-4-yl)(4-((4-(pyrrolidin-1-yl)but-2-ynyloxy)methyl)-1*H*-1,2,3-triazol-1-yl)methyl)-5-vinylquinuclidine (**27**) ($CDCl_3$, 400 MHz). 31
Figure S30. ^{13}C NMR of (2*R*,4*S*,5*R*)-2-((*R*)-(6-methoxyquinolin-4-yl)(4-((4-(pyrrolidin-1-yl)but-2-ynyloxy)methyl)-1*H*-1,2,3-triazol-1-yl)methyl)-5-vinylquinuclidine (**27**) ($CDCl_3$, 125 MHz). 32
Figure S31. ^{1}H NMR of (2*R*,4*S*,5*R*)-2-((*S*)-(6-methoxyquinolin-4-yl)(4-((4-(*piperidin*-1-yl)but-2-ynyloxy)methyl)-1*H*-1,2,3-triazol-1-yl)methyl)-5-vinylquinuclidine (**24**) ($CDCl_3$, 300 MHz). 33
Figure S32. ^{13}C NMR of (2*R*,4*S*,5R)-2-((*S*)-(6-methoxyquinolin-4-yl)(4-((4-(*piperidin*-1-yl)but-2-ynyloxy)methyl)-1*H*-1,2,3-triazol-1-yl)methyl)-5-vinylquinuclidine (**24**) ($CDCl_3$, 126 MHz). 34
Figure S33. ^{1}H NMR of (2*R*,4*S*,5*R*)-2-((*R*)-(6-methoxyquinolin-4-yl)(4-((4-(piperidin-1-yl)but-2-ynyloxy)methyl)-1*H*-1,2,3-triazol-1-yl)methyl)-5-vinylquinuclidine (**28**) ($CDCl_3$, 300 MHz). 35
Figure S34. ^{13}C NMR of (2*R*,4*S*,5*R*)-2-((*R*)-(6-methoxyquinolin-4-yl)(4-((4-(piperidin-1-yl)but-2-ynyloxy)methyl)-1*H*-1,2,3-triazol-1-yl)methyl)-5-vinylquinuclidine (**28**) ($CDCl_3$, 126 MHz). 36
Figure S35. ^{1}H NMR spectrum of (2*R*,4*S*,5*R*)-2-((*S*)-(4-((4-(azepan-1-yl)but-2-ynyloxy)methyl)-1*H*-1,2,3-triazol-1-yl)(6-methoxyquinolin-4-yl)methyl)-5-vinylquinuclidine (**25**) ($CDCl_3$, 300 MHz). 37
Figure S36. ^{13}C NMR spectrum of (2*R*,4*S*,5*R*)-2-((*S*)-(4-((4-(azepan-1-yl)but-2-ynyloxy)methyl)-1*H*-1,2,3-triazol-1-yl)(6-methoxyquinolin-4-yl)methyl)-5-vinylquinuclidine (**25**) ($CDCl_3$, 101 MHz). 38
Figure S37. ^{1}H NMR spectrum of (2*R*,4*S*,5*R*)-2-((*R*)-(4-((4-(azepan-1-yl)but-2-ynyloxy)methyl)-1*H*-1,2,3-triazol-1-yl)(6-methoxyquinolin-4-yl)methyl)-5-vinylquinuclidine (**29**) ($CDCl_3$, 300 MHz). 39
Figure S38. ^{13}C NMR spectrum of (2*R*,4*S*,5*R*)-2-((*R*)-(4-((4-(azepan-1-yl)but-2-ynyloxy)methyl)-1*H*-1,2,3-triazol-1-yl)(6-methoxyquinolin-4-yl)methyl)-5-vinylquinuclidine (**29**) ($CDCl_3$, 126 MHz). 40
Figure S39. ^{1}H NMR spectrum of (2*R*,4*S*,5R)-2-((*S*)-(4-((4-(azocan-1-yl)but-2-ynyloxy)methyl)-1*H*-1,2,3-triazol-1-yl)(6-methoxyquinolin-4-yl)methyl)-5-vinylquinuclidine (**26**) ($CDCl_3$, 400 MHz). 41
Figure S40. ^{13}C NMR spectrum of (2*R*,4*S*,5*R*)-2-((*S*)-(4-((4-(azocan-1-yl)but-2-ynyloxy)methyl)-1*H*-1,2,3-triazol-1-yl)(6-methoxyquinolin-4-yl)methyl)-5-vinylquinuclidine (**26**) ($CDCl_3$, 75 MHz). 42

Figure S41. ^{1}H NMR spectrum of (2*R*,4*S*,5*R*)-2-((*R*)-(4-((4-(azocan-1-yl)but-2-ynyloxy)methyl)-1*H*-1,2,3-triazol-1-yl)(6-methoxyquinolin-4-yl)methyl)-5-vinylquinuclidine (**30**) ($CDCl_3$, 400 MHz). 43
Figure S42. ^{13}C NMR spectrum of (2*R*,4*S*,5*R*)-2-((*R*)-(4-((4-(azocan-1-yl)but-2-ynyloxy)methyl)-1*H*-1,2,3-triazol-1-yl)(6-methoxyquinolin-4-yl)methyl)-5-vinylquinuclidine (**30**) ($CDCl_3$, 100 MHz). 44

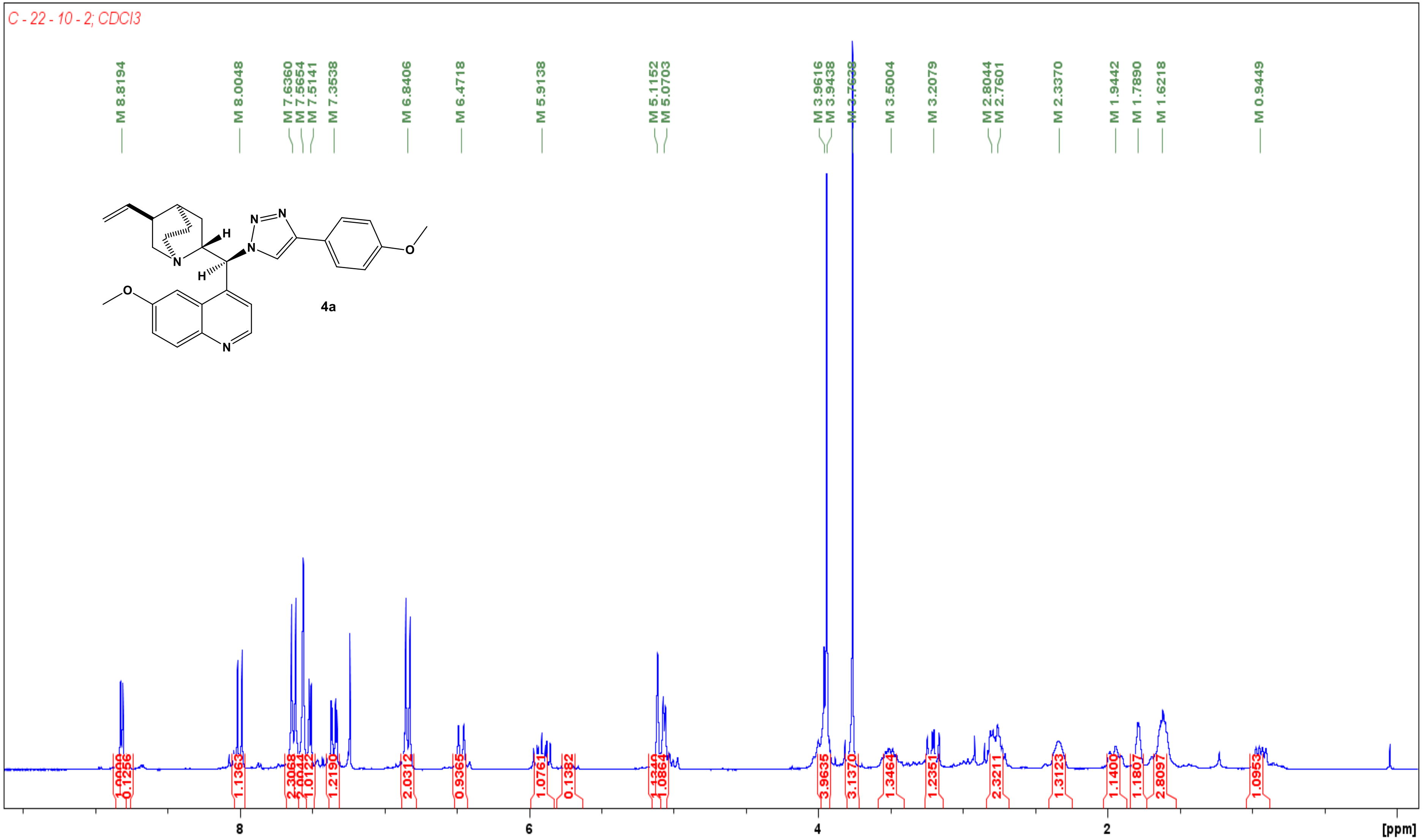

Figure S1. ^{1}H NMR spectrum of (2*R*,4*S*,5*S*)-2-((*S*)-(4-(4-methoxyphenyl)-1*H*-1,2,3-triazol-1-yl)(6-methoxyquinolin-4-yl)methyl)-5-vinylquinuclidine **(4a)** ($CDCl_3$, 300 MHz).

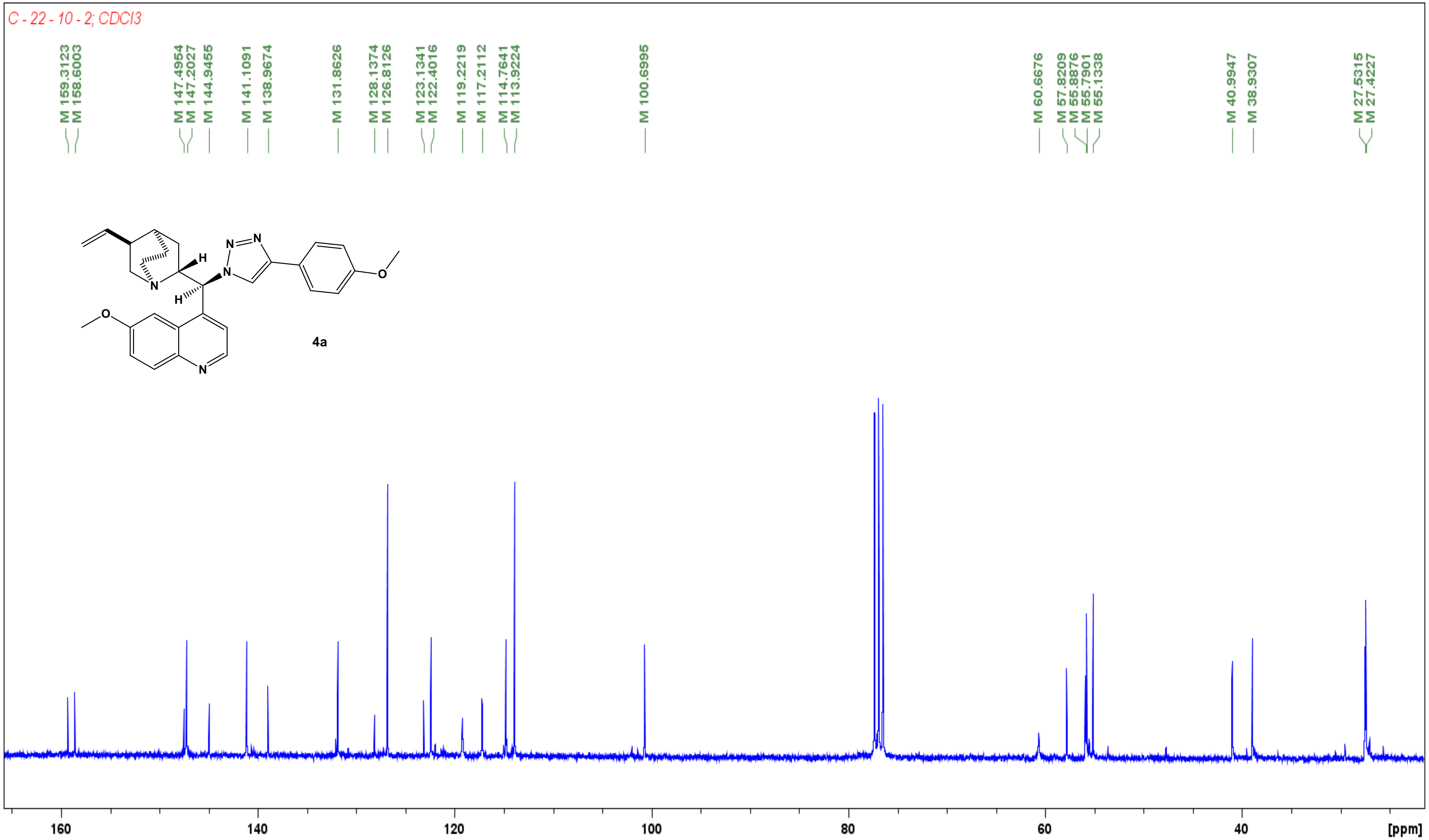

Figure S2. ^{13}C NMR spectrum of (2*R*,4*S*,5*S*)-2-((*S*)-(4-(4-methoxyphenyl)-1*H*-1,2,3-triazol-1-yl)(6-methoxyquinolin-4-yl)methyl*)-5-vinylquinuclidine* **(4a)** ($CDCl_3$, 75 MHz).

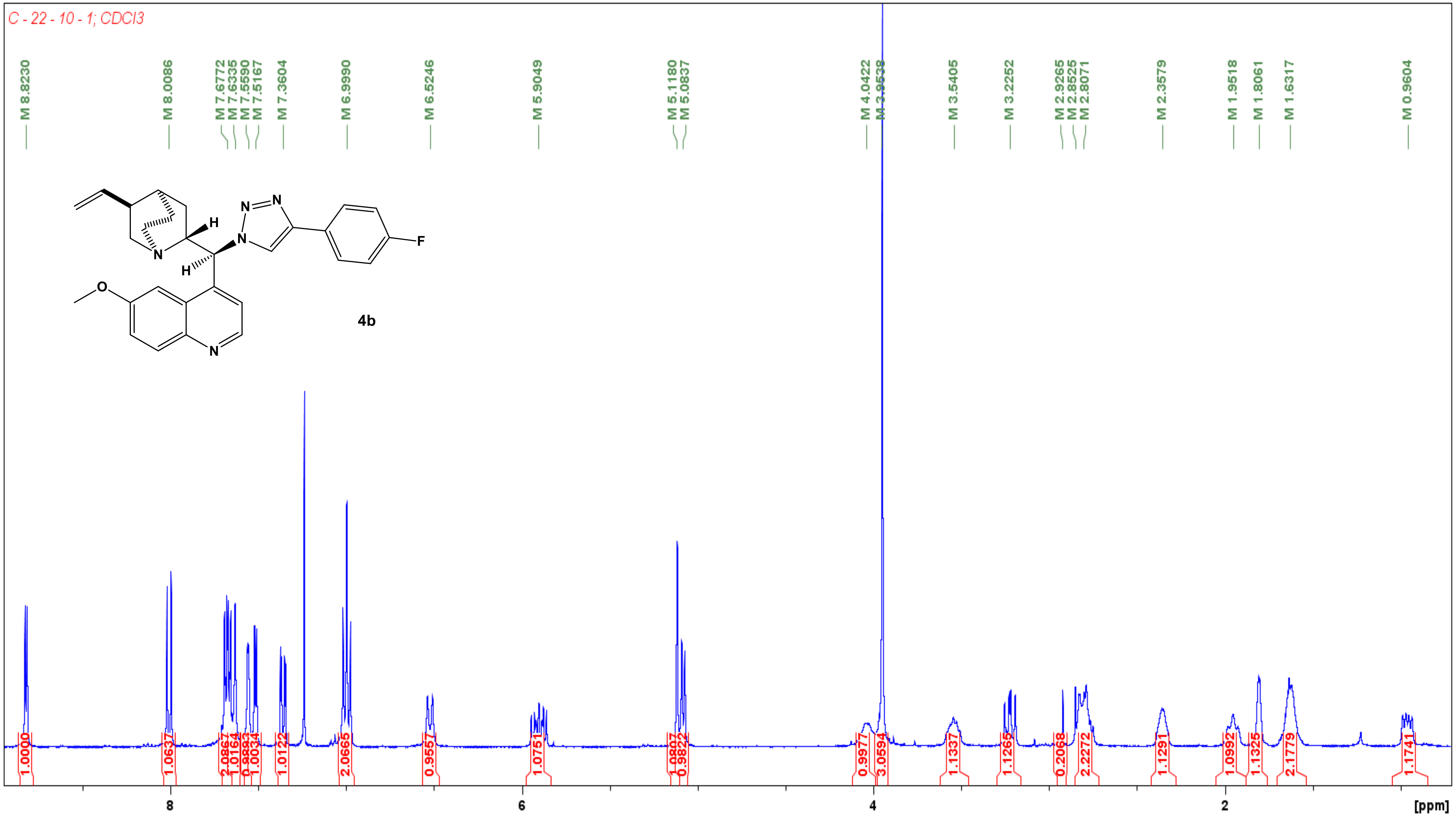

Figure S3. ¹H NMR spectrum of (2*R*,4*S*,5*S*)-2-((*S*)-(4-(4-fluorophenyl)-1*H*-1,2,3-triazol-1-yl)(6-methoxyquinolin-4-yl)methyl)*-5-vinylquinuclidine* **(4b)** ($CDCl_3$, 400 MHz).

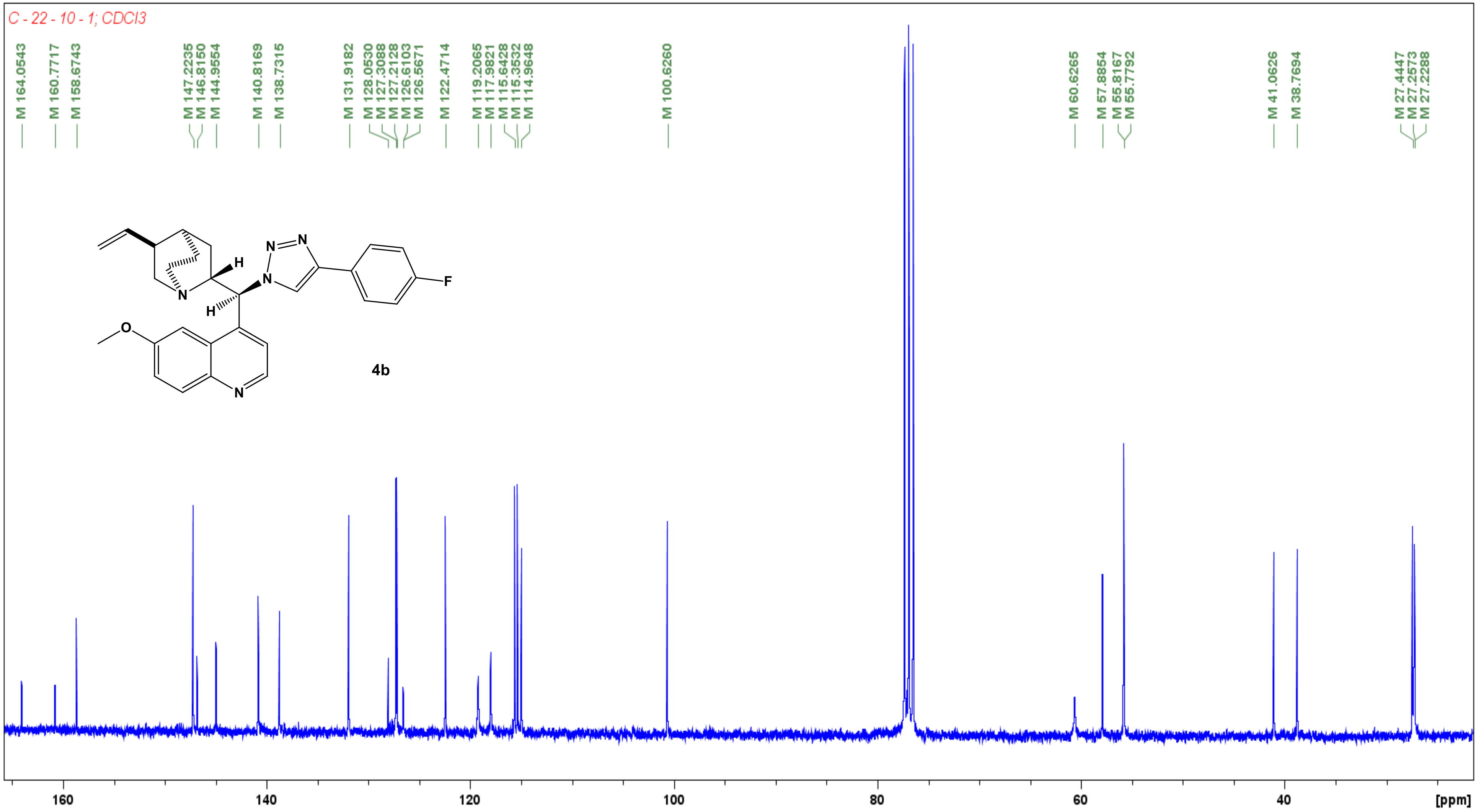

Figure S4. ^{13}C NMR spectrum of (2*R*,4*S*,5*S*)-2-((*S*)-(4-(4-fluorophenyl)-1*H*-1,2,3-triazol-1-yl)(6-methoxyquinolin-4-yl)methyl*)-5-vinylquinuclidine* **(4b)** ($CDCl_3$, 75 MHz).

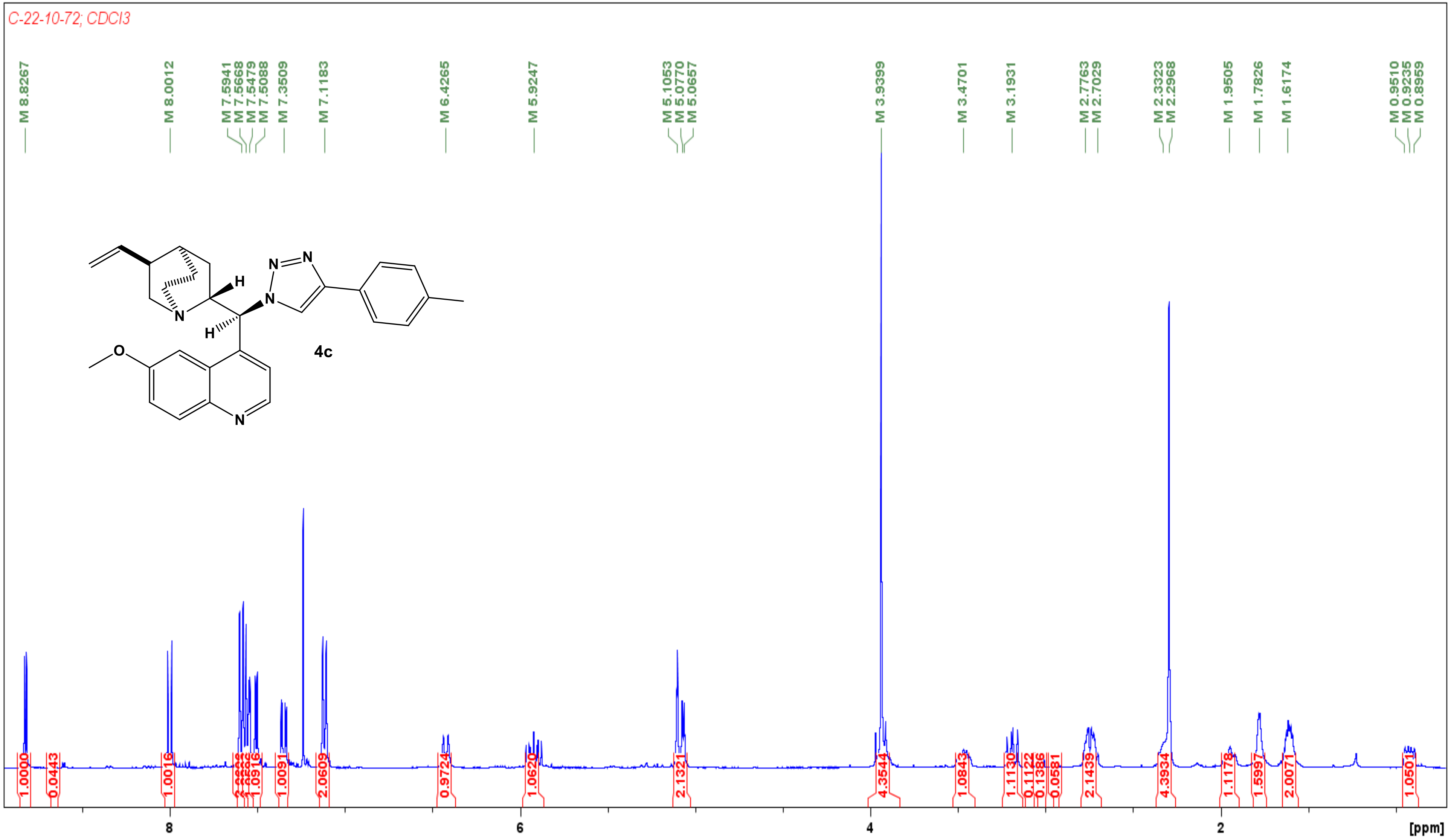

Figure S5. ^{1}H NMR spectrum of (2*R*,4*S*,5*S*)-2-((*S*)-(6-methoxyquinolin-4-yl)(4-*p*-tolyl-1*H*-1,2,3-triazol-1-yl)methyl)-5-vinylquinuclidine **(4c)** ($CDCl_3$, 400 MHz).

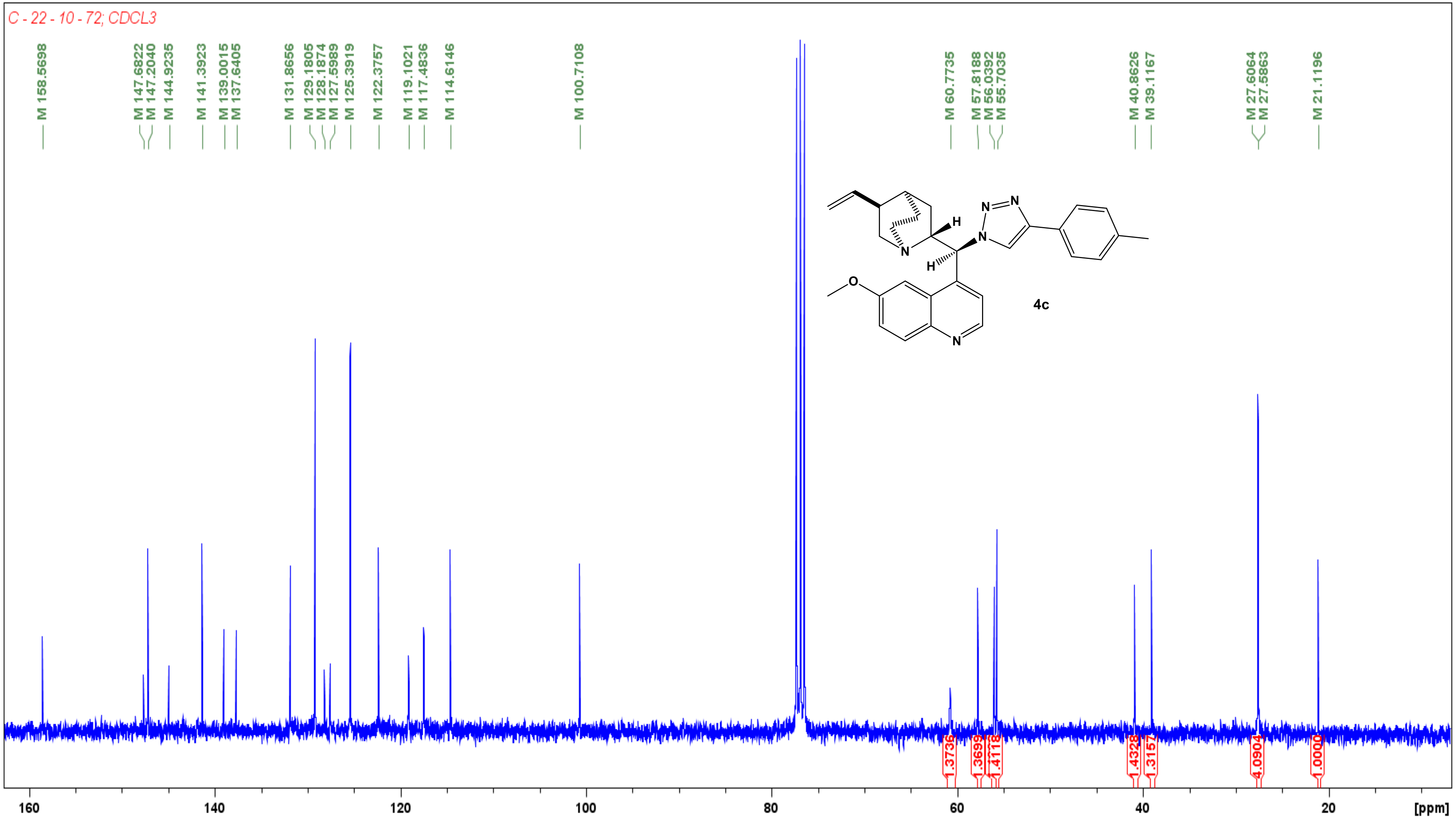

Figure S6. ^{13}C NMR spectrum of (2*R*,4*S*,5*S*)-2-((*S*)-(6-methoxyquinolin-4-yl)(4-*p*-tolyl-1*H*-1,2,3-triazol-1-yl)methyl)-5-vinylquinuclidine **(4c)** ($CDCl_3$, 75 MHz).

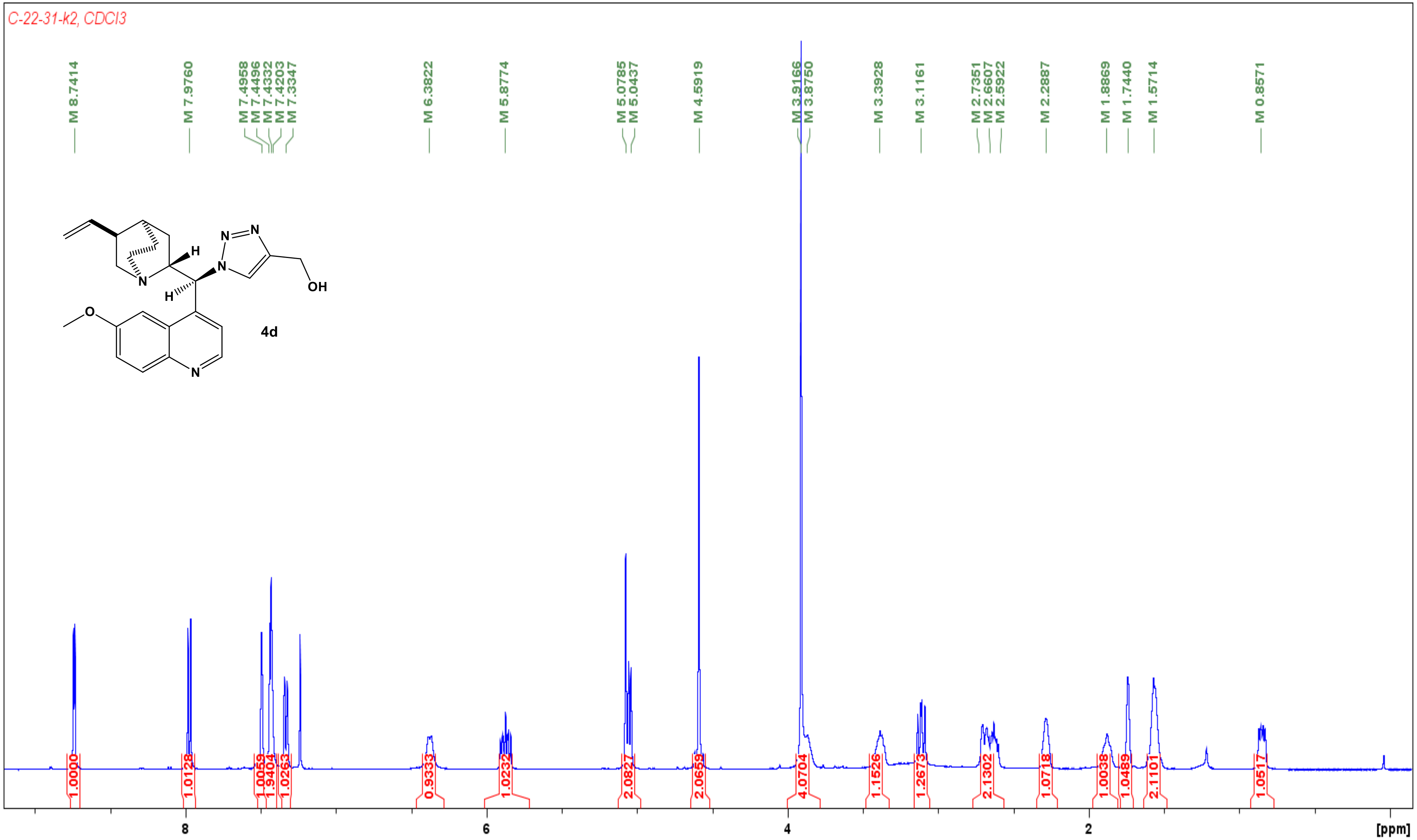

Figure S7. ¹H NMR spectrum of (1-((*S*)-(6-methoxyquinolin-4-yl)((2*R*,4*S*,5*R*)-5-vinylquinuclidin-2-yl)methyl)-1*H*-1,2,3-triazol-4-yl)methanol (**4d**) ($CDCl_3$, 500 MHz).

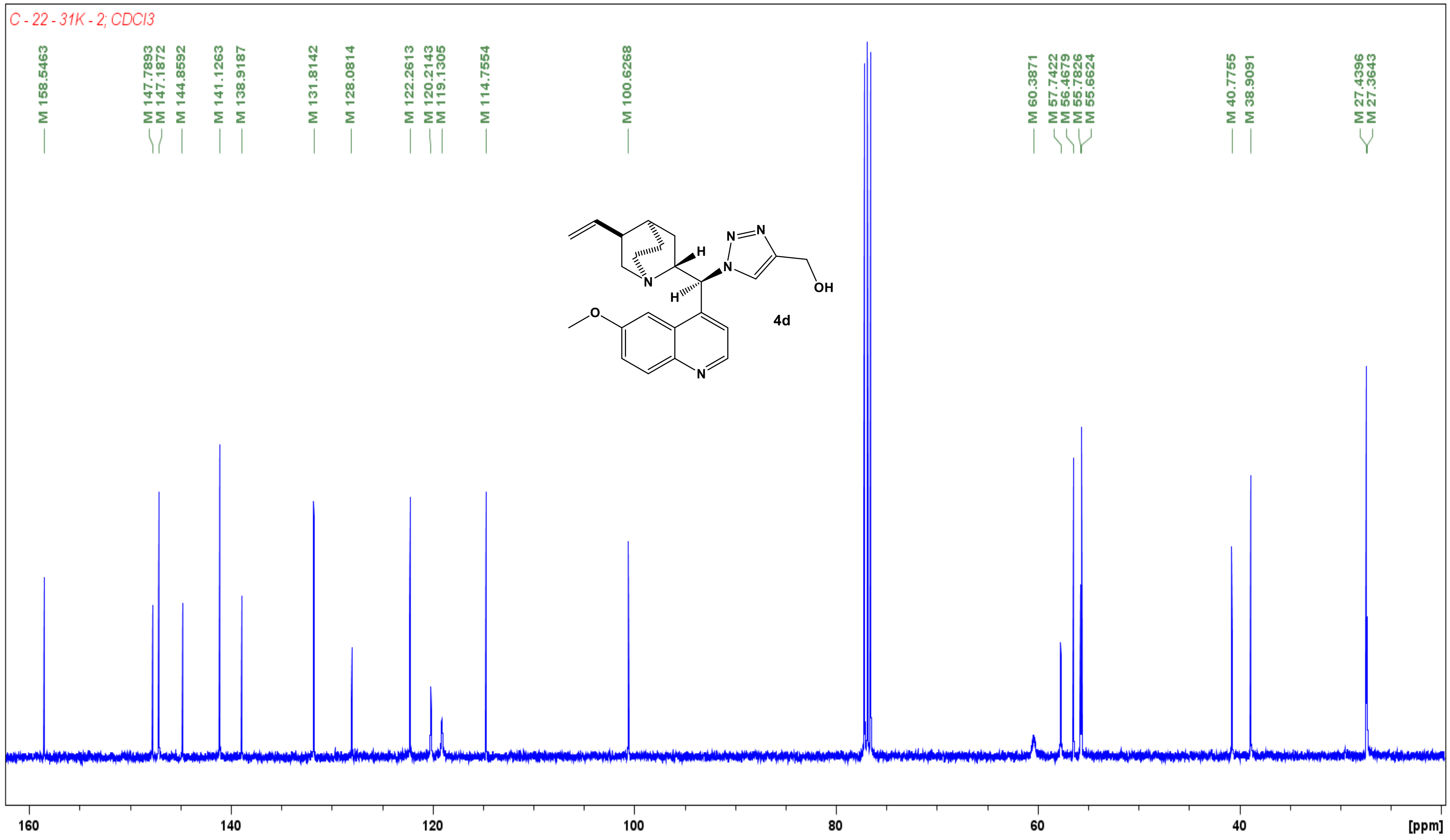

Figure S8. ^{13}C NMR spectrum of (1-((*S*)-(6-methoxyquinolin-4-yl)((2*R*,4*S*,5*R*)-5-vinylquinuclidin-2-yl)methyl)-1*H*-1,2,3-triazol-4-yl)methanol (**4d**) ($CDCl_3$, 100 MHz).

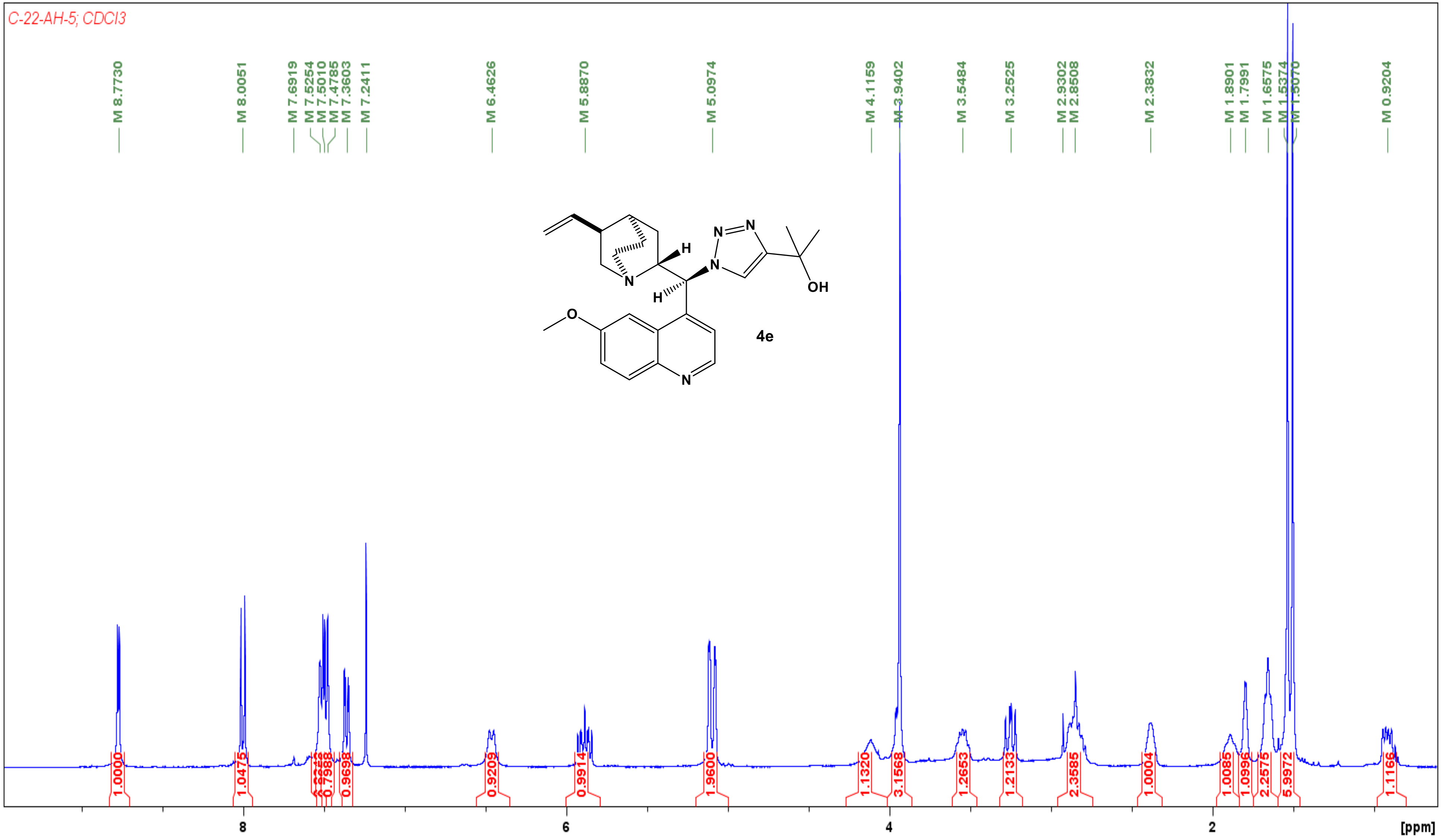

Figure S9. ^{1}H NMR spectrum of 2-(1-((*S*)-(6-methoxyquinolin-4-yl)((2*S*,4*S*,5*R*)-5-vinylquinuclidin-2-yl)methyl)-1*H*-1,2,3-triazol-4-yl)propan-2-ol (**4e**) ($CDCl_3$, 400 MHz).

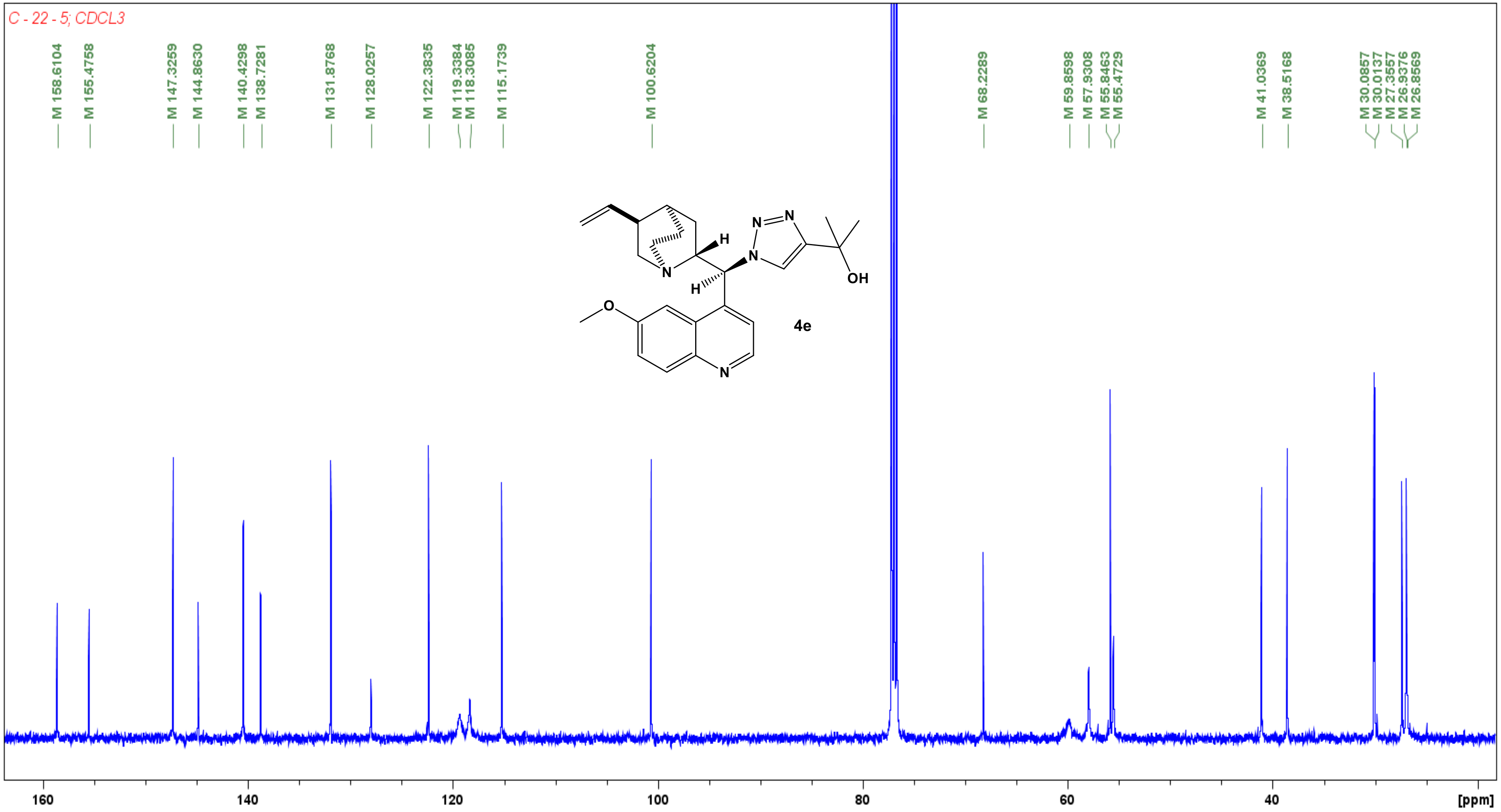

Figure S10. ^{13}C NMR spectrum of 2-(1-((*S*)-(6-methoxyquinolin-4-yl)((2*S*,4*S*,5*R*)-5-vinylquinuclidin-2-yl)methyl)-1*H*-1,2,3-triazol-4-yl)propan-2-ol (**4e**) ($CDCl_3$, 126 MHz).

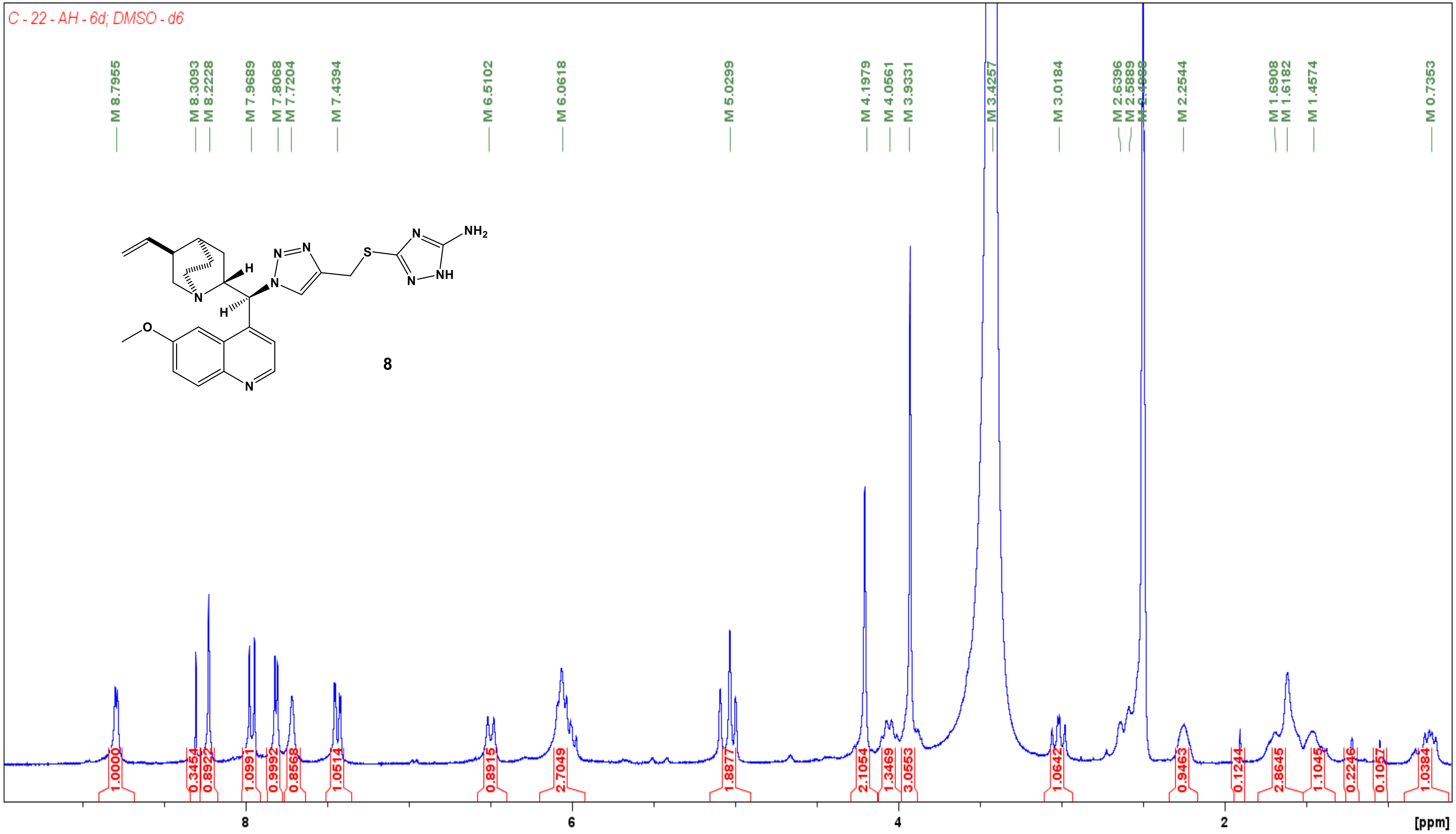

Figure S11. ^{1}H NMR spectrum of 3-((1-((*S*)-(6-methoxyquinolin-4-yl)((2*R*,4*S*,5*R*)-5-vinylquinuclidin-2-yl)methyl)-1*H*-1,2,3-triazol-4-yl)methylthio)-1*H*-1,2,4-triazol-5-amine (**8**) (($(CD_3)_2SO$, 300 MHz).

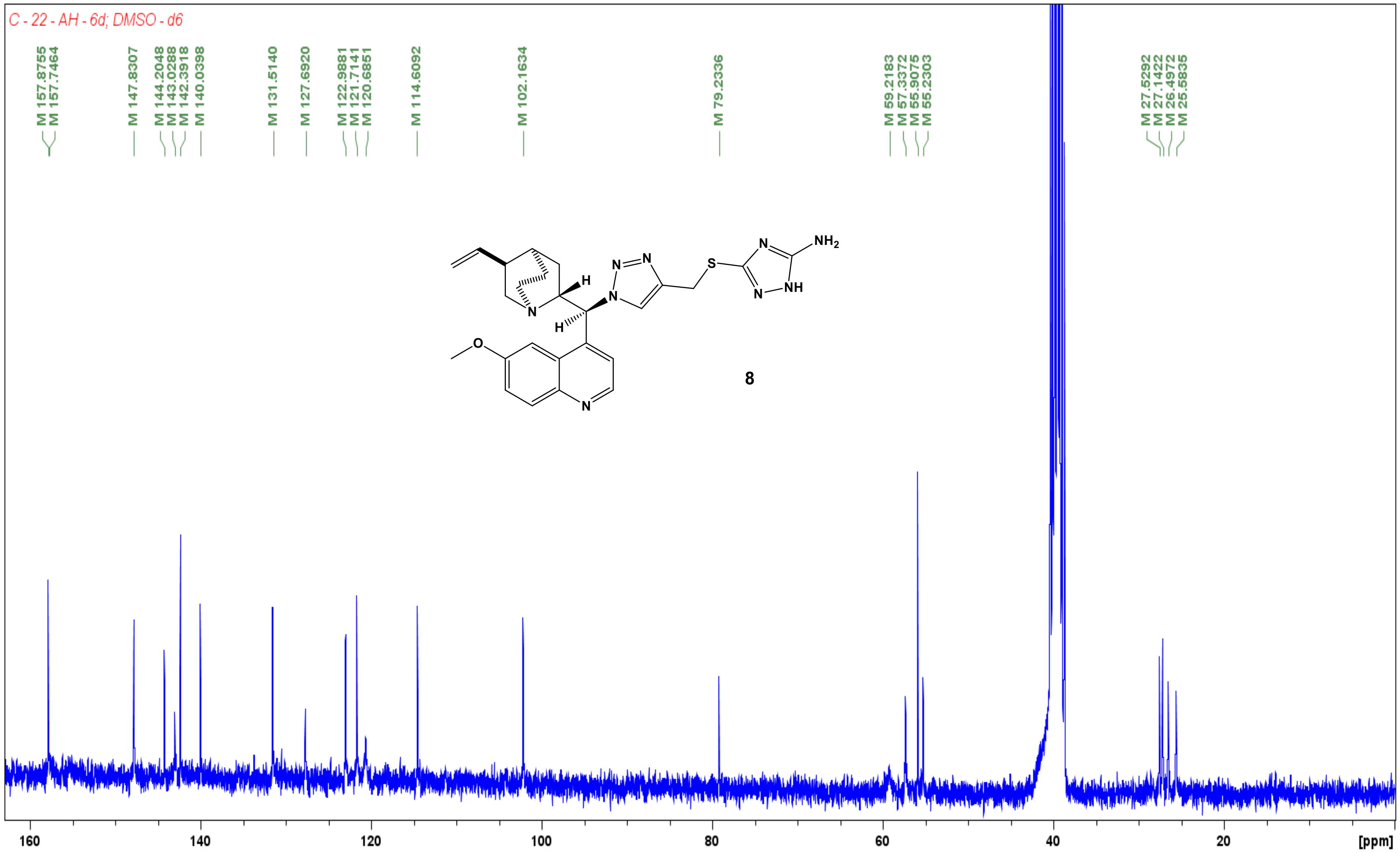

Figure S12. ^{13}C NMR spectrum of 3-((1-((*S*)-(6-methoxyquinolin-4-yl)((2*R*,4*S*,5*R*)-5-vinylquinuclidin-2-yl)methyl)-1*H*-1,2,3-triazol-4-yl)methylthio)-1*H*-1,2,4-triazol-5-amine (**8**) ($(CD_3)_2SO$, 75 MHz).

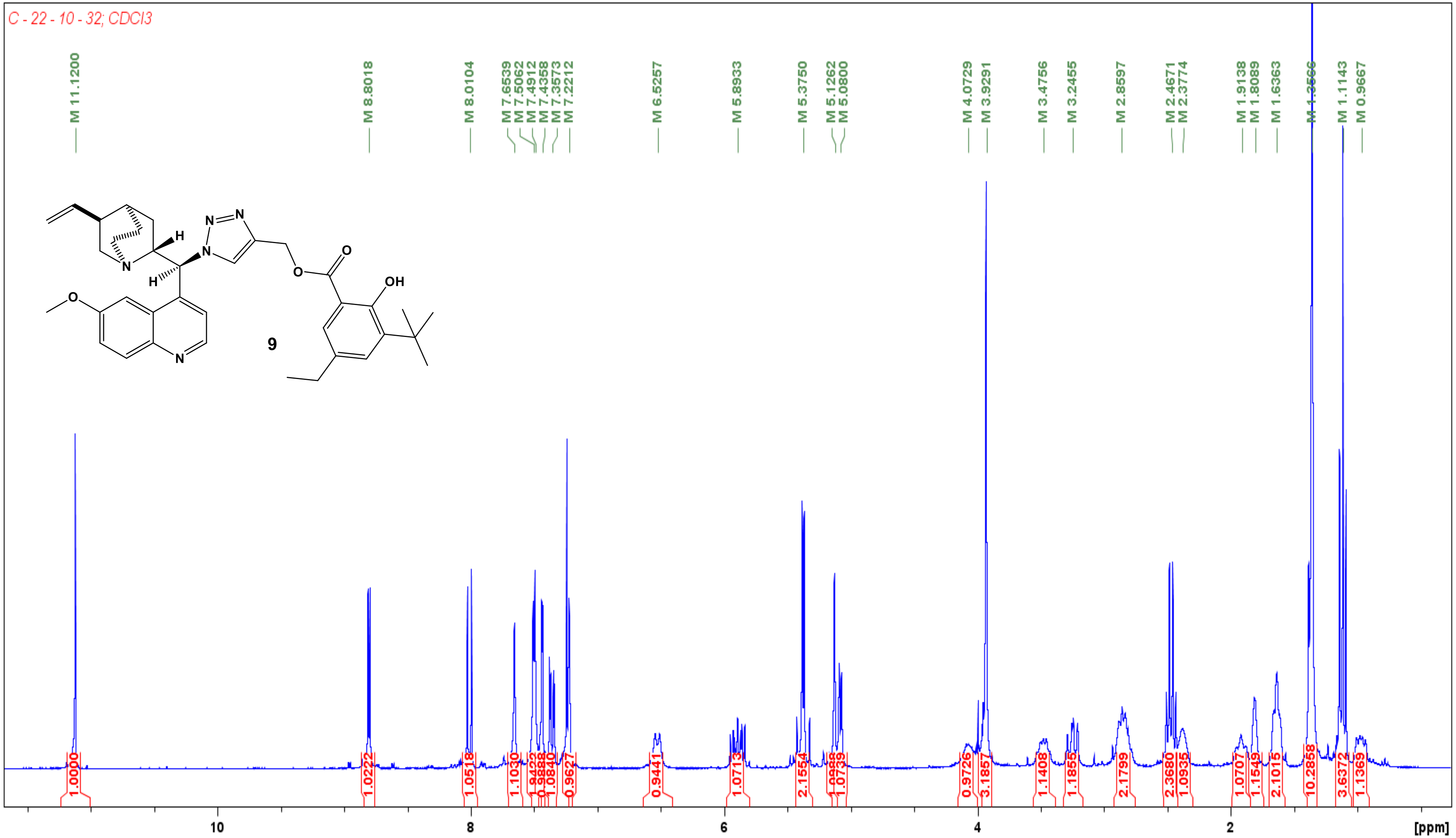

Figure S13. ^{1}H NMR spectrum of (1-((*S*)-(6-methoxyquinolin-4-yl)((2*R*,4*S*,5*R*)-5-vinylquinuclidin-2-yl)-methyl)-1H-1,2,3-triazol-4-yl)methyl 3-tert-butyl-5-ethyl-2-hydroxybenzoate (**9**) ($CDCl_3$, 300 MHz).

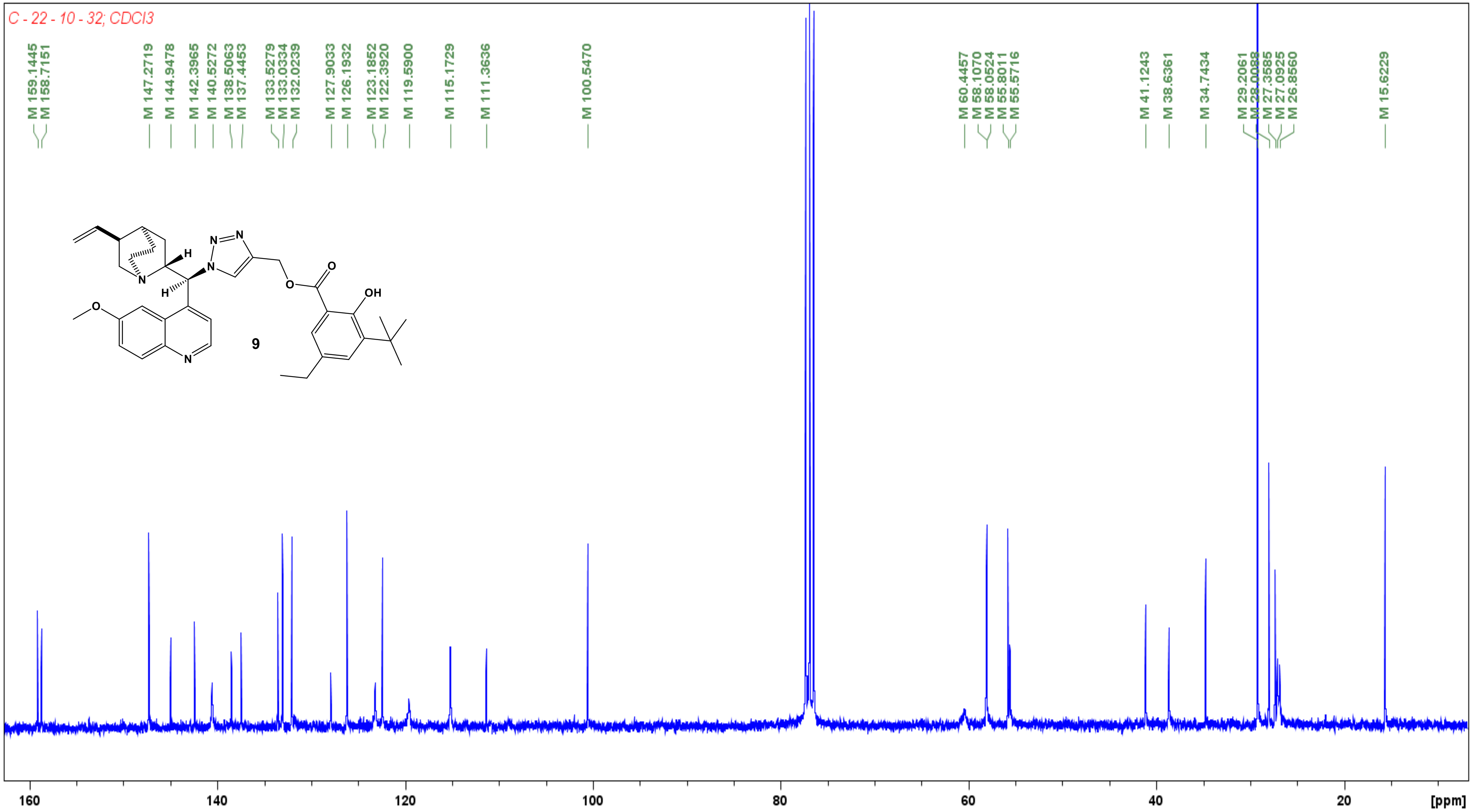

Figure S14. ^{1}H NMR spectrum of (1-((*S*)-(6-methoxyquinolin-4-yl)((2*R*,4*S*,5*R*)-5-vinylquinuclidin-2-yl)-methyl)-1H-1,2,3-triazol-4-yl)methyl 3-tert-butyl-5-ethyl-2-hydroxybenzoate (**9**) ($CDCl_3$, 300 MHz).

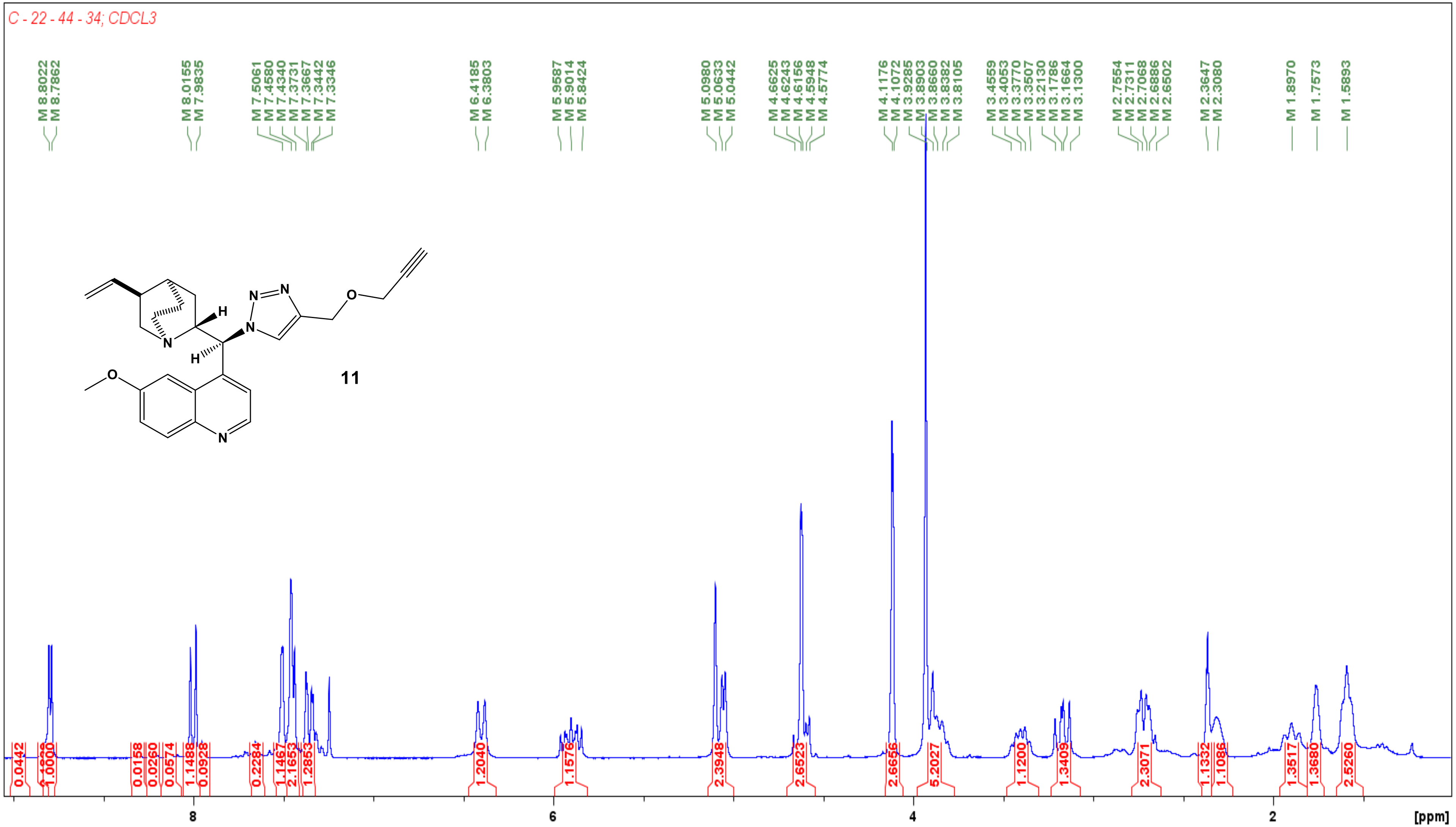

Figure S15. ^{1}H NMR spectrum of (2*R*,4*S*,5*R*)-2-((*S*)-(6-methoxyquinolin-4-yl)(4-((prop-2-ynyloxy)methyl)-1*H*-1,2,3-triazol-1-yl)methyl)-5-vinylquinuclidine (**11**) ($CDCl_3$, 300 MHz).

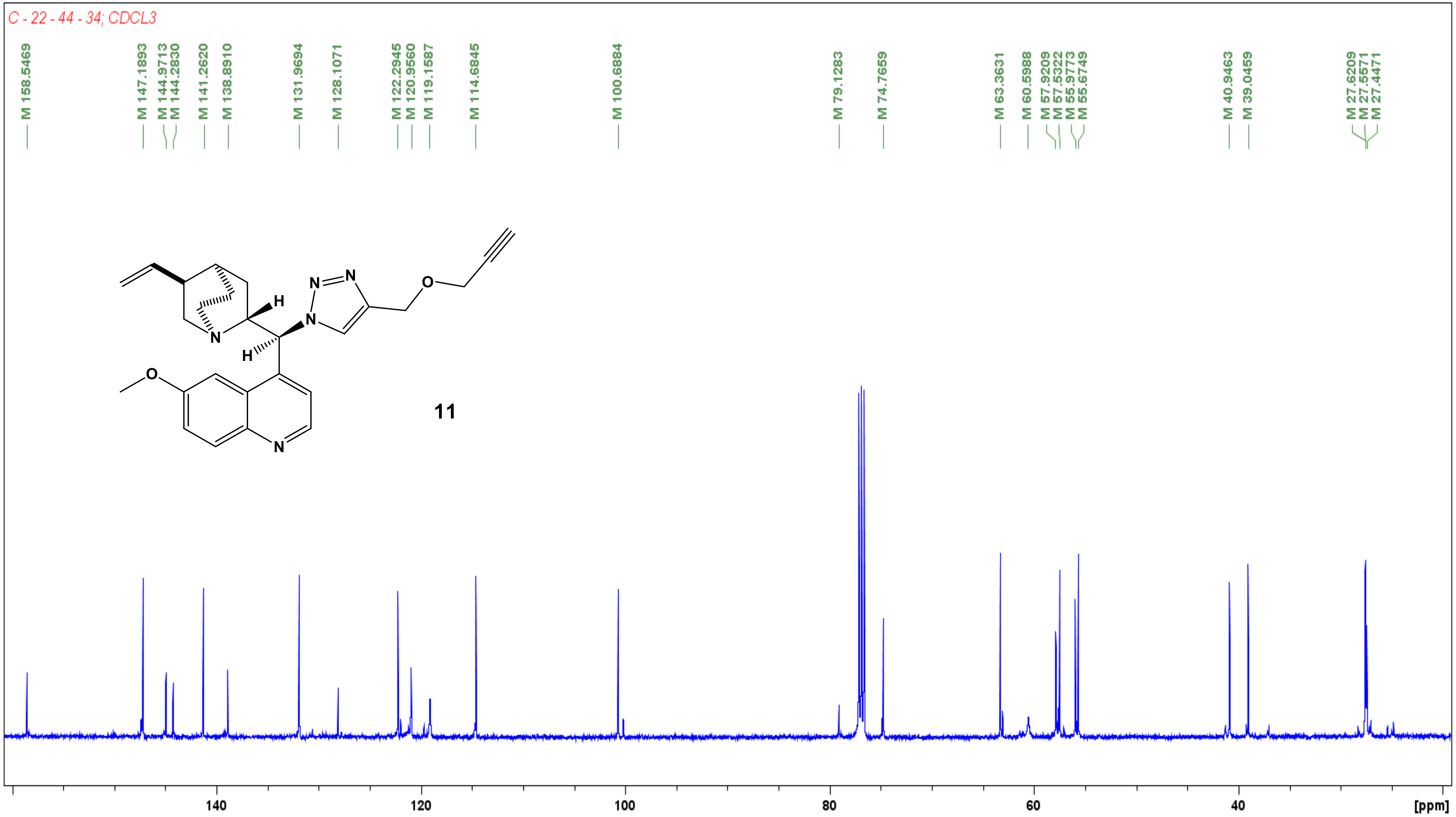

Figure S16. ¹³C NMR spectrum of (2*R*,4*S*,5*R*)-2-((*S*)-(6-methoxyquinolin-4-yl)(4-((prop-2-ynyloxy)methyl)-1*H*-1,2,3-triazol-1-yl)methyl)-5-vinylquinuclidine (**11**) ($CDCl_3$, 100 MHz).

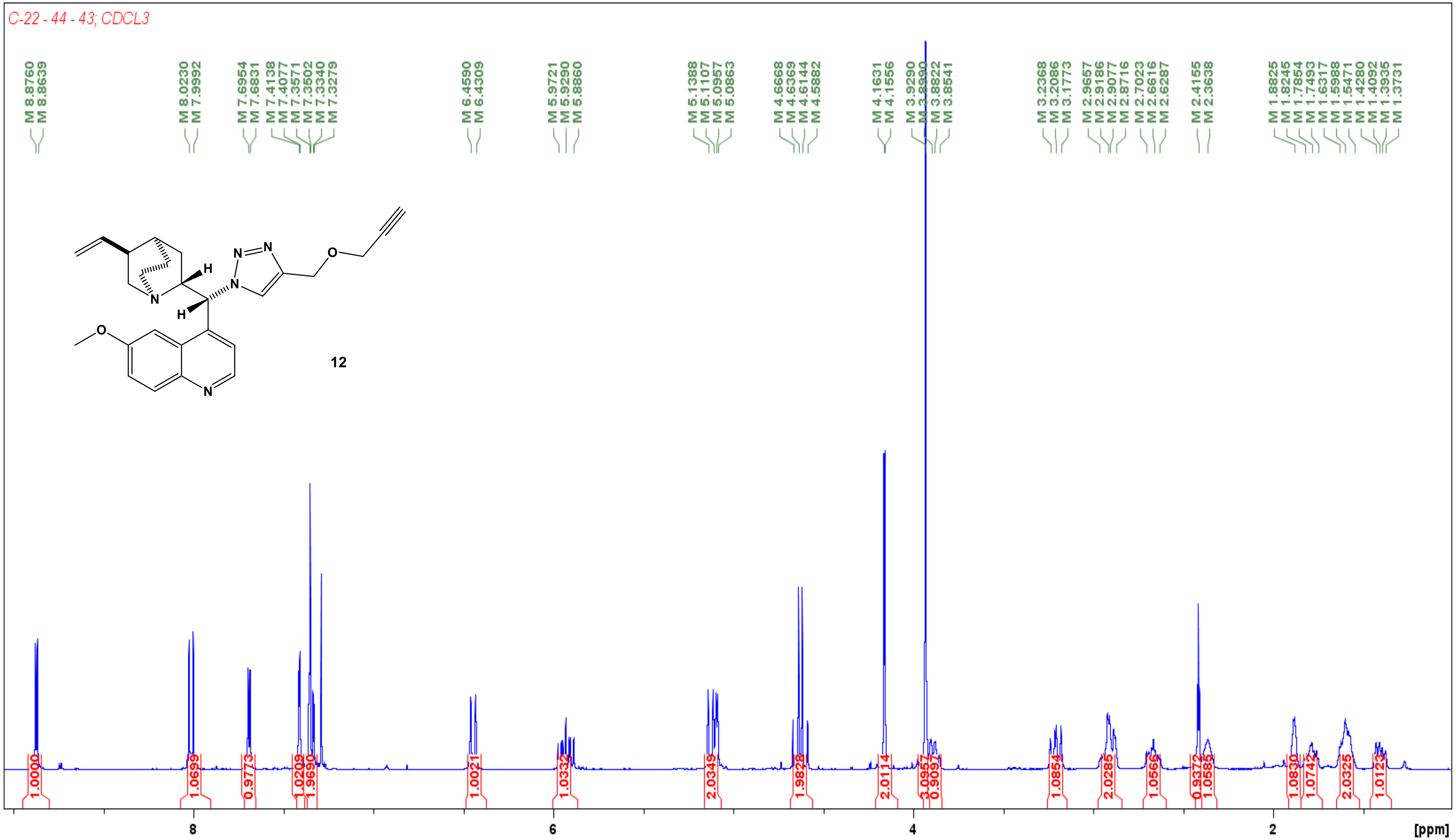

Figure S17. ^{1}H NMR spectrum of (2*R*,4*S*,5*R*)-2-((*R*)-(6-methoxyquinolin-4-yl)(4-((prop-2-ynyloxy)methyl)-1*H*-1,2,3-triazol-1-yl)methyl)-5-vinylquinuclidine (**12**) ($CDCl_3$, 400 MHz).

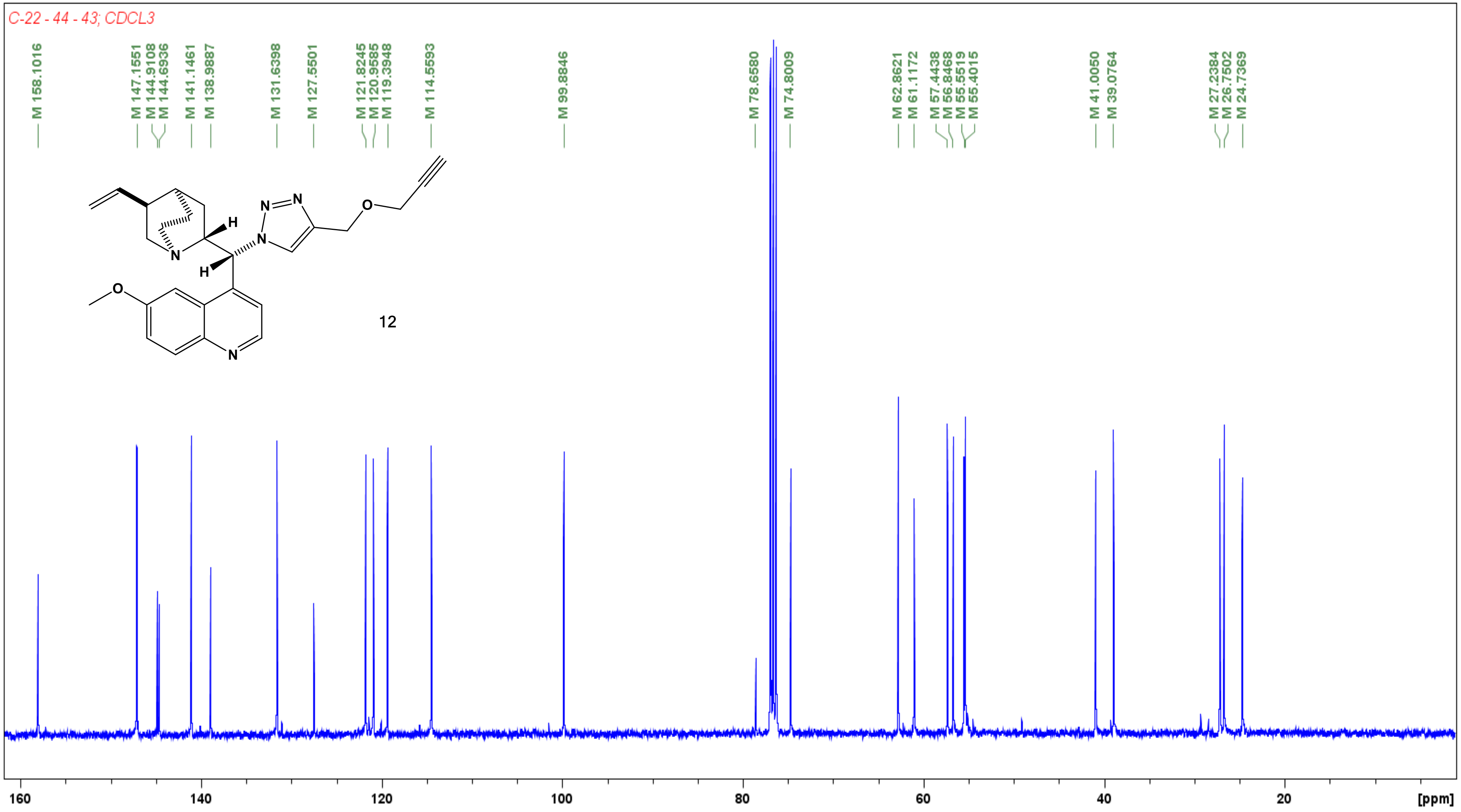

Figure S18. ^{13}C NMR spectrum of (2*R*,4*S*,5*R*)-2-((*S*)-(6-methoxyquinolin-4-yl)(4-((prop-2-ynyloxy)methyl)-1*H*-1,2,3-triazol-1-yl)methyl)-5-vinylquinuclidine (**12**) ($CDCl_3$, 101 MHz).

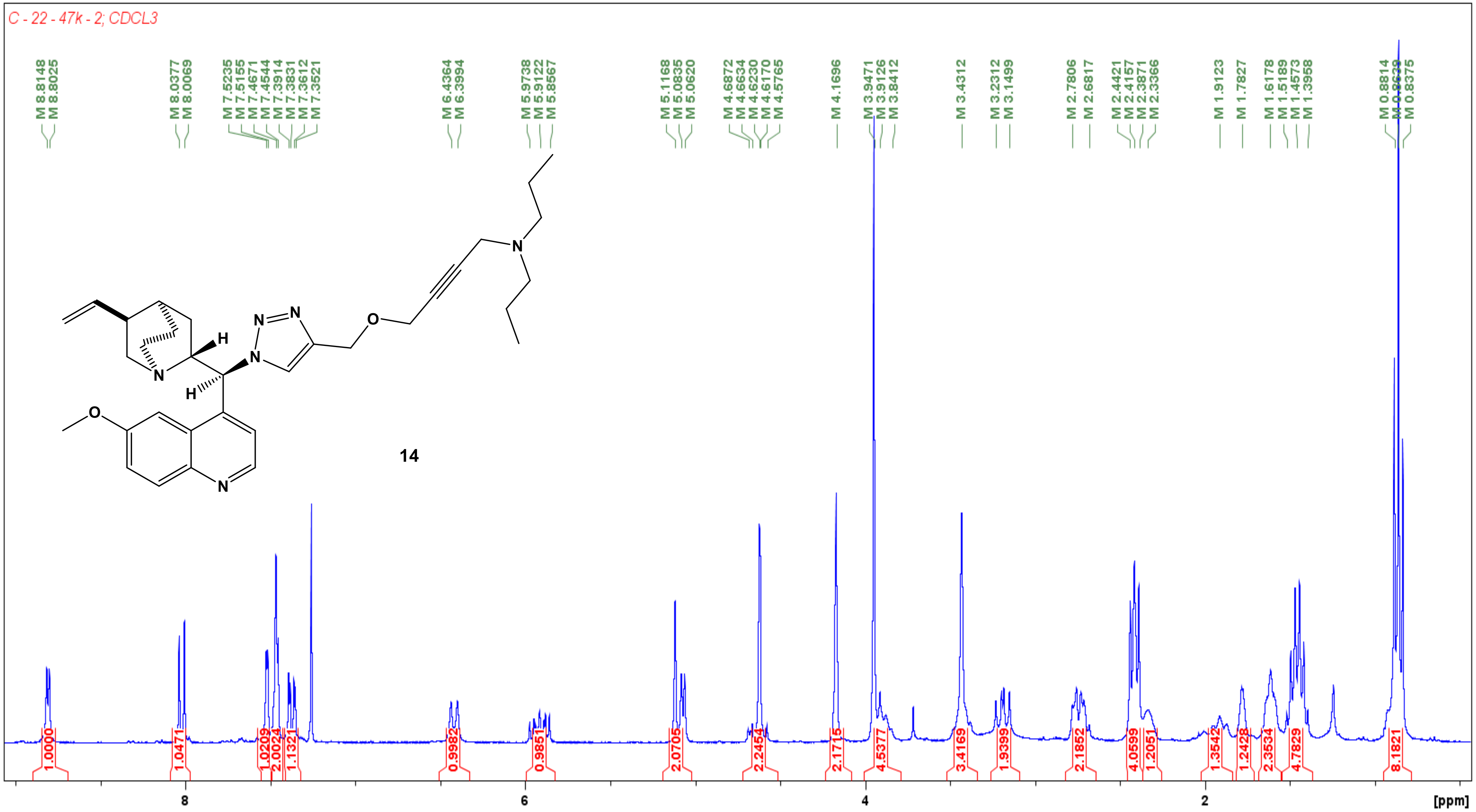

Figure S19. ¹H NMR spectrum of 4-((1-((*S*)-(6-methoxyquinolin-4-yl)((2*R*,4*S*,5*R*)-5-vinylquinuclidin-2-yl)methyl)-1*H*-1,2,3-triazol-4-yl)methoxy)-N,N-dipropylbut-2-yn-1-amine (**14**) ($CDCl_3$, 300 MHz).

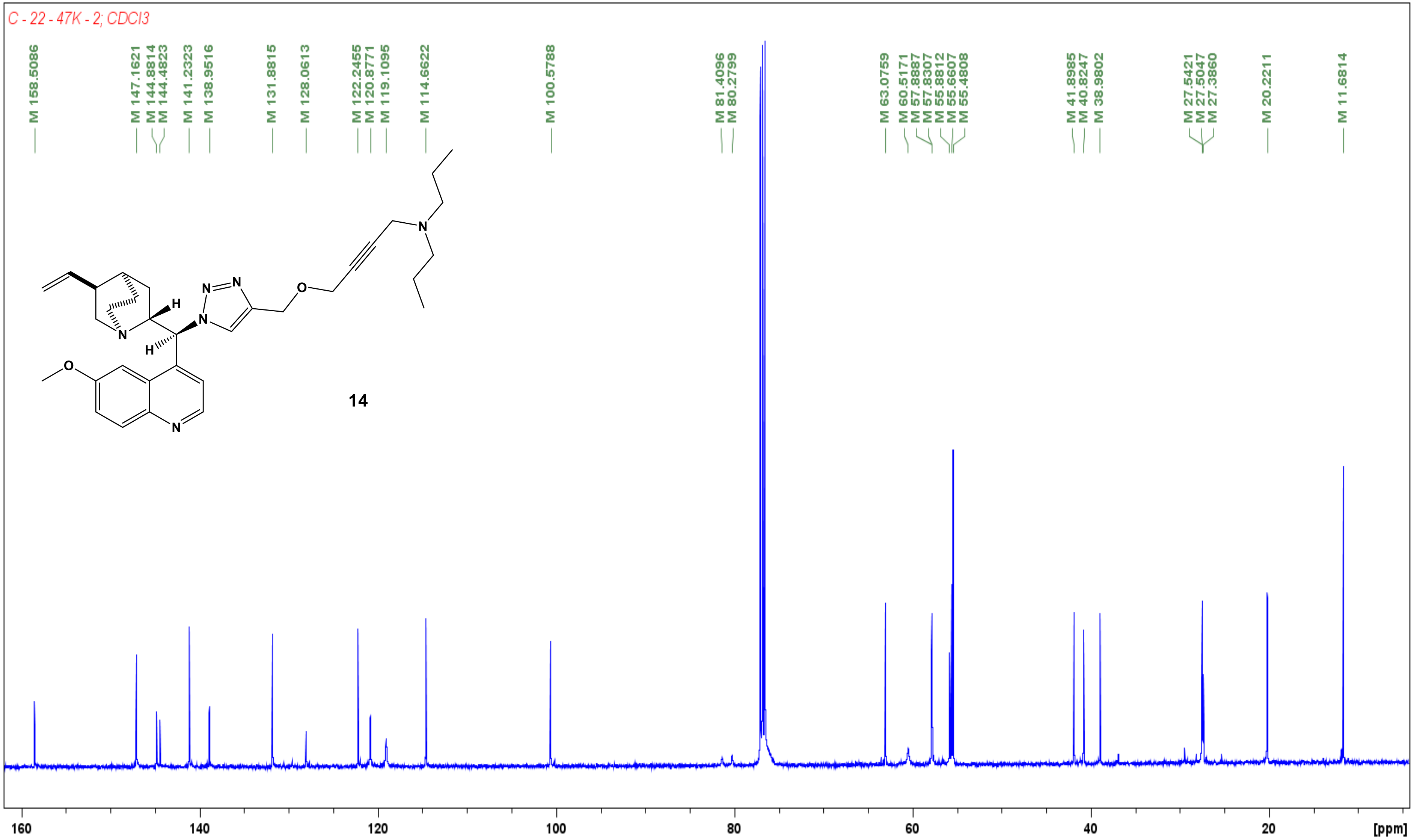

Figure S20. ^{13}C NMR spectrum of 4-((1-((*S*)-(6-methoxyquinolin-4-yl)((2*R*,4*S*,5*R*)-5-vinylquinuclidin-2-yl)methyl)-1*H*-1,2,3-triazol-4-yl)methoxy)-N,N-dipropylbut-2-yn-1-amine (**14**) ($CDCl_3$, 126 MHz).

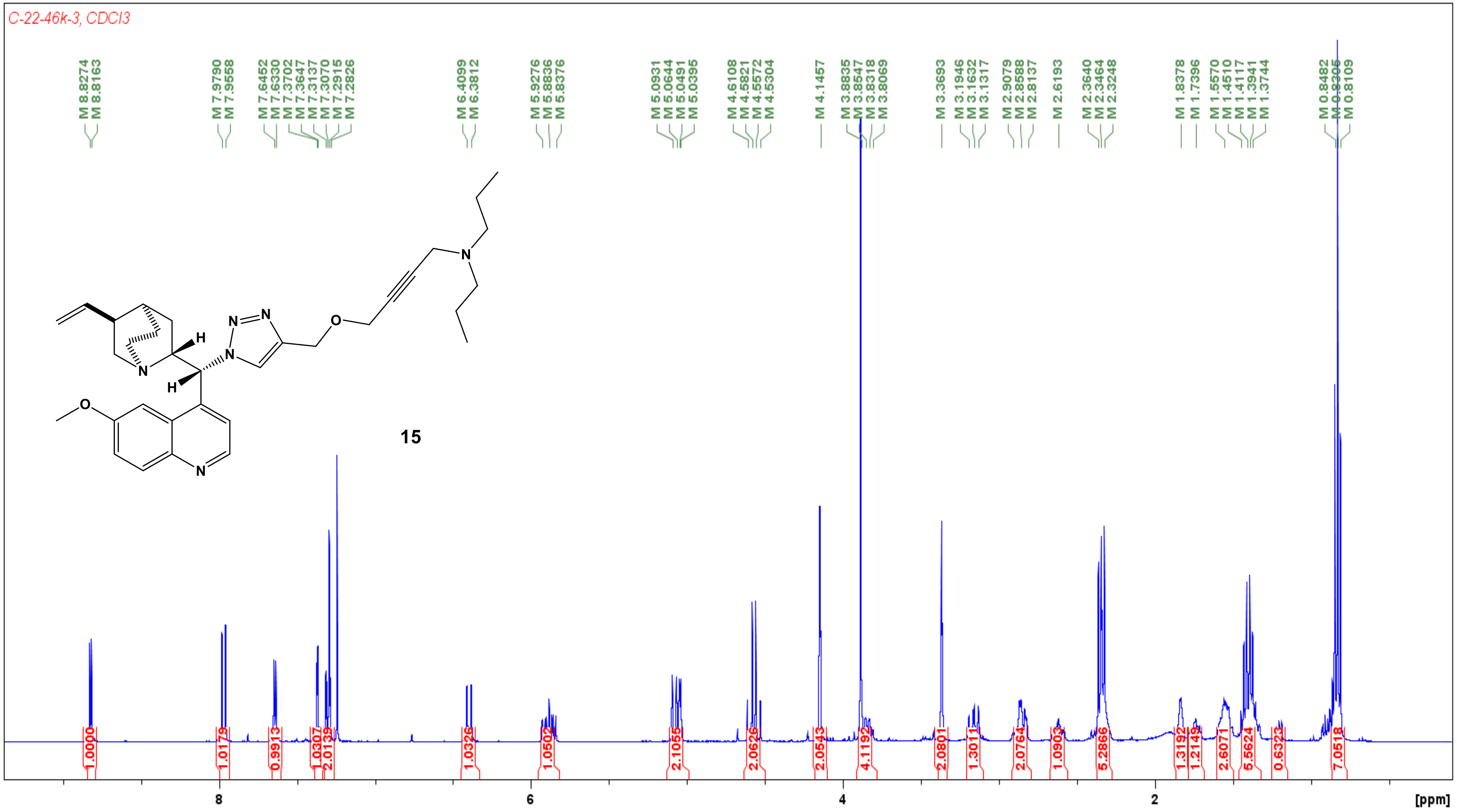

Figure S21. ^{1}H NMR spectrum of 4-((1-((*R*)-(6-methoxyquinolin-4-yl)((2*R*,4*S*,5*R*)-5-vinylquinuclidin-2-yl)methyl)-1*H*-1,2,3-triazol-4-yl)methoxy)-N,N-dipropylbut-2-yn-1-amine (**15**) ($CDCl_3$, 400 MHz).

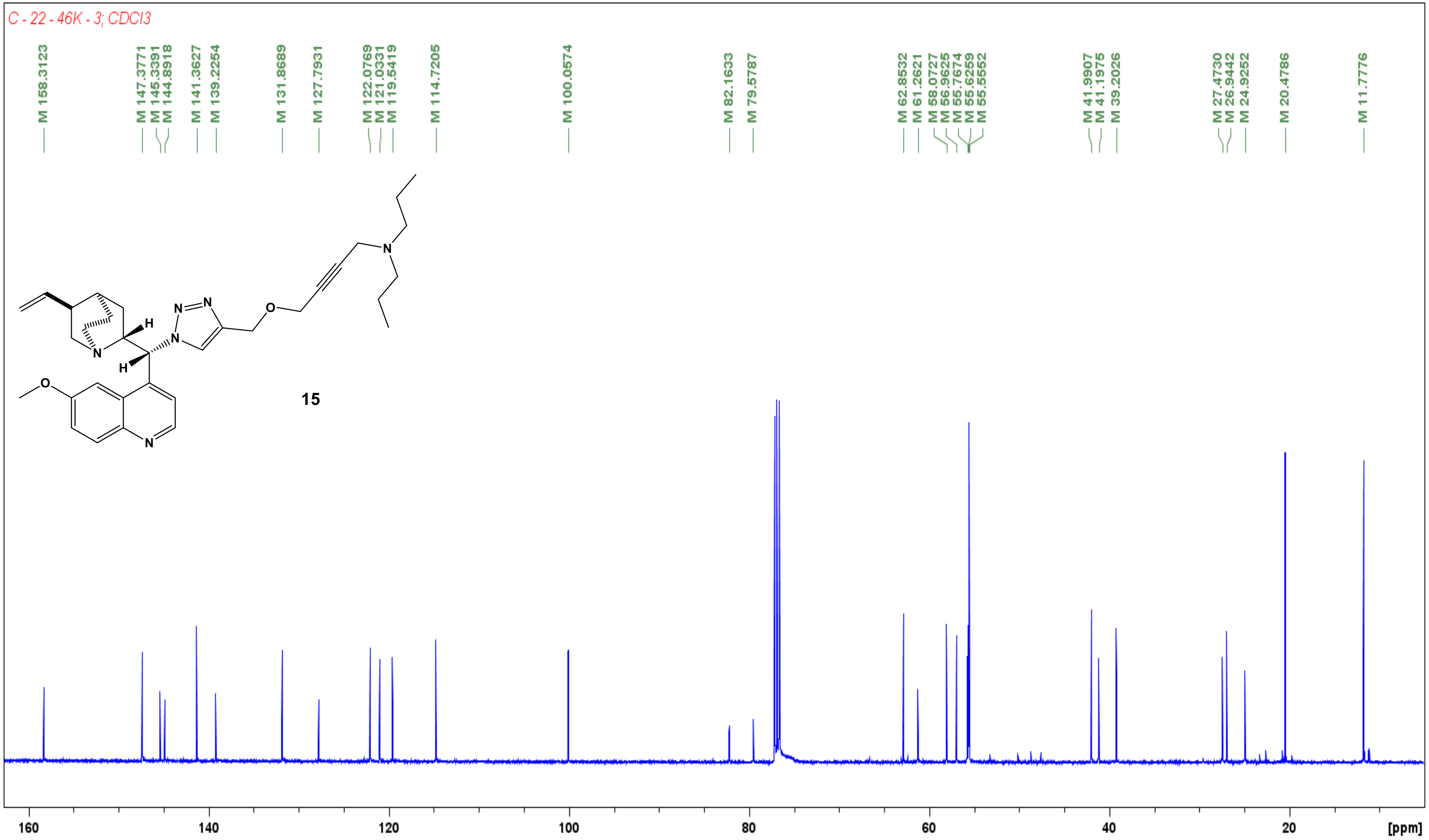

Figure S22. ^{13}C NMR spectrum of 4-((1-((*R*)-(6-methoxyquinolin-4-yl)((2*R*,4*S*,5*R*)-5-vinylquinuclidin-2-yl)methyl)-1*H*-1,2,3-triazol-4-yl)methoxy)-N,N-dipropylbut-2-yn-1-amine (**15**) ($CDCl_3$, 126 MHz).

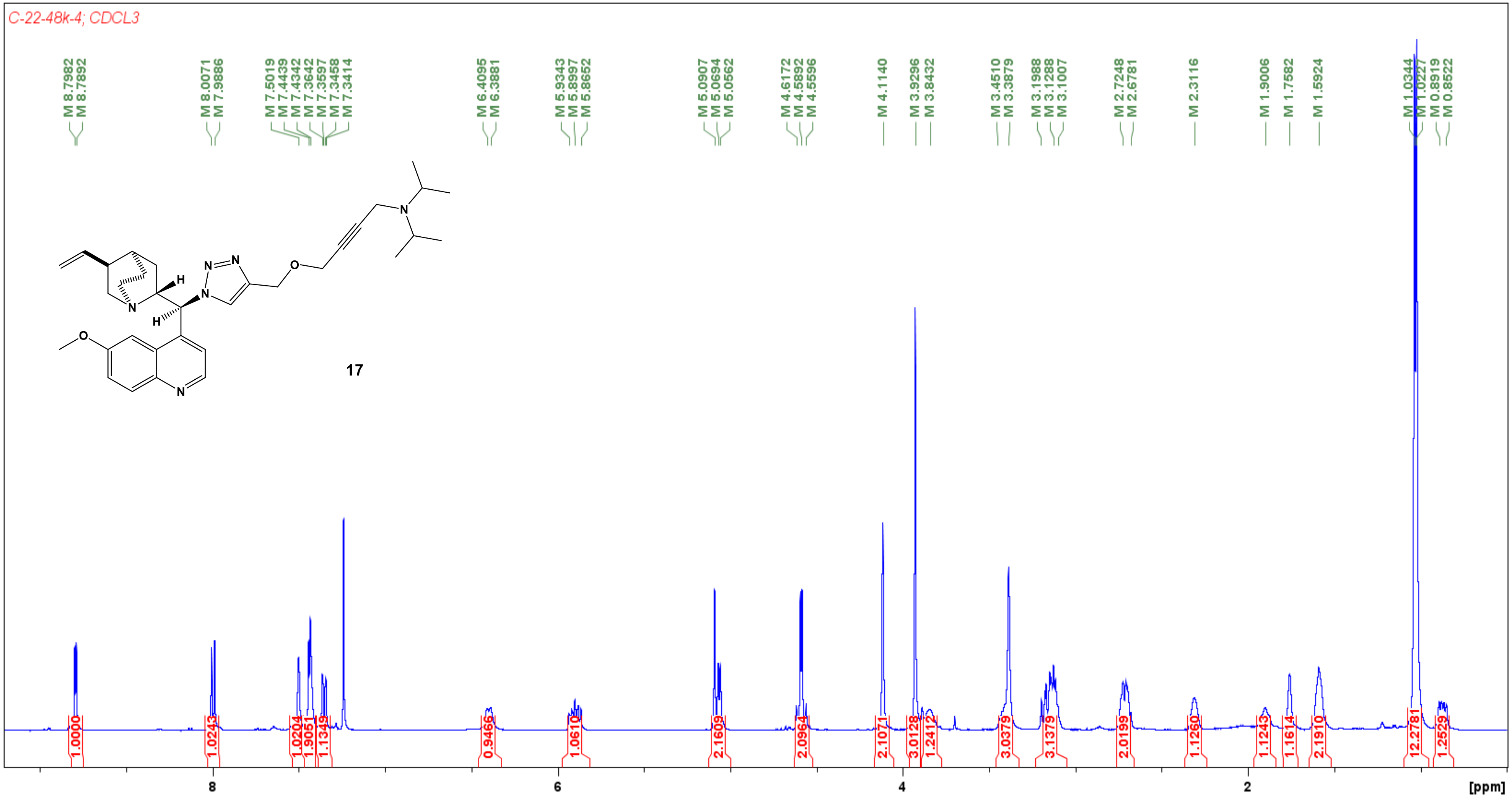

Figure S23. ¹H NMR spectrum of 4-((1-((*S*)-(6-methoxyquinolin-4-yl)((2*R*,4*S*,5*R*)-5-vinylquinuclidin-2-yl)methyl)-1*H*-1,2,3-triazol-4-yl)methoxy)-N,N-diisopropylbut-2-yn-1-amine (**17**) ($CDCl_3$, 500 MHz).

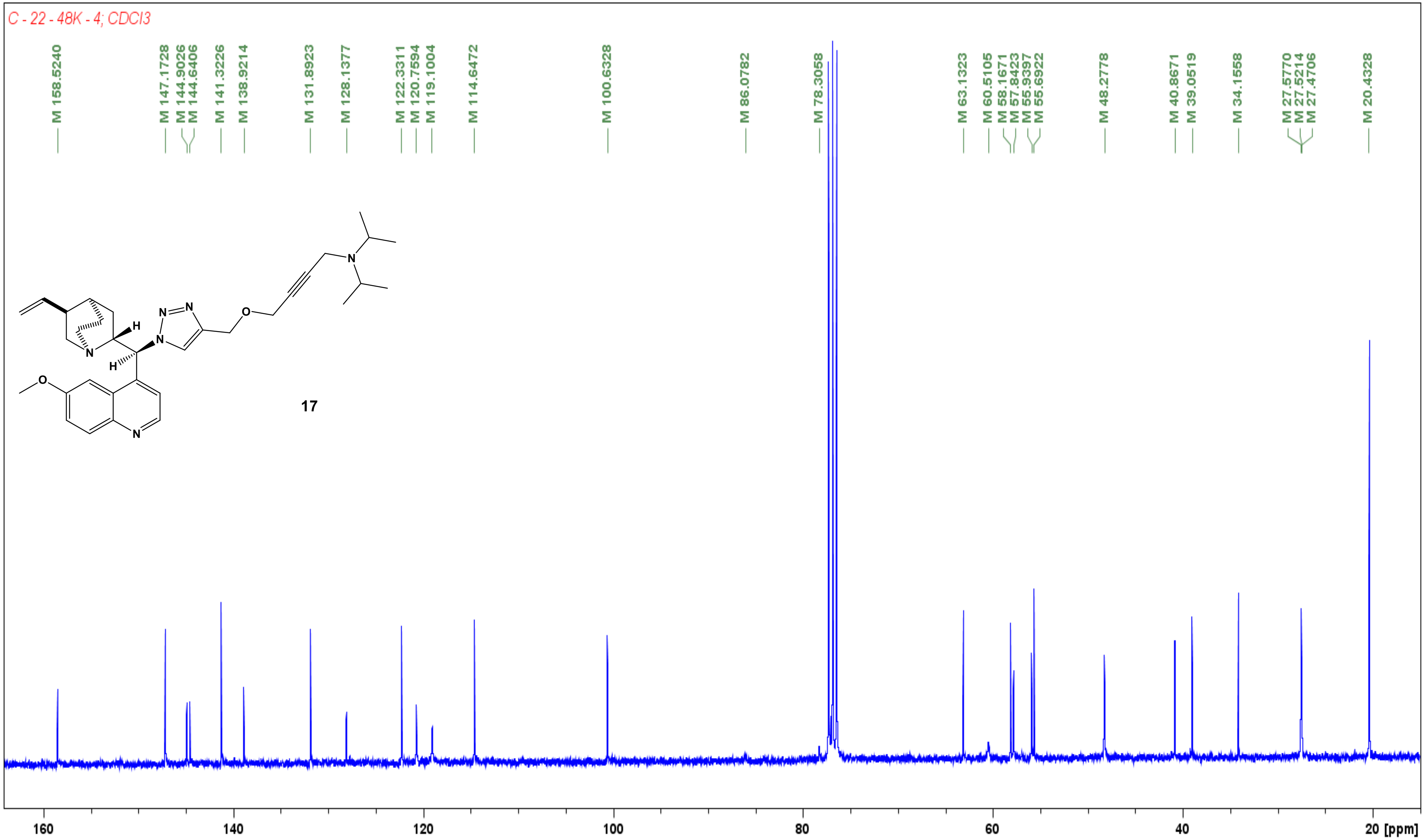

Figure S24. ^{13}C NMR spectrum of 4-((1-((*S*)-(6-methoxyquinolin-4-yl)((2*R*,4*S*,5*R*)-5-vinylquinuclidin-2-yl)methyl)-1*H*-1,2,3-triazol-4-yl)methoxy)-N,N-diisopropylbut-2-yn-1-amine (**17**) ($CDCl_3$, 75 MHz).

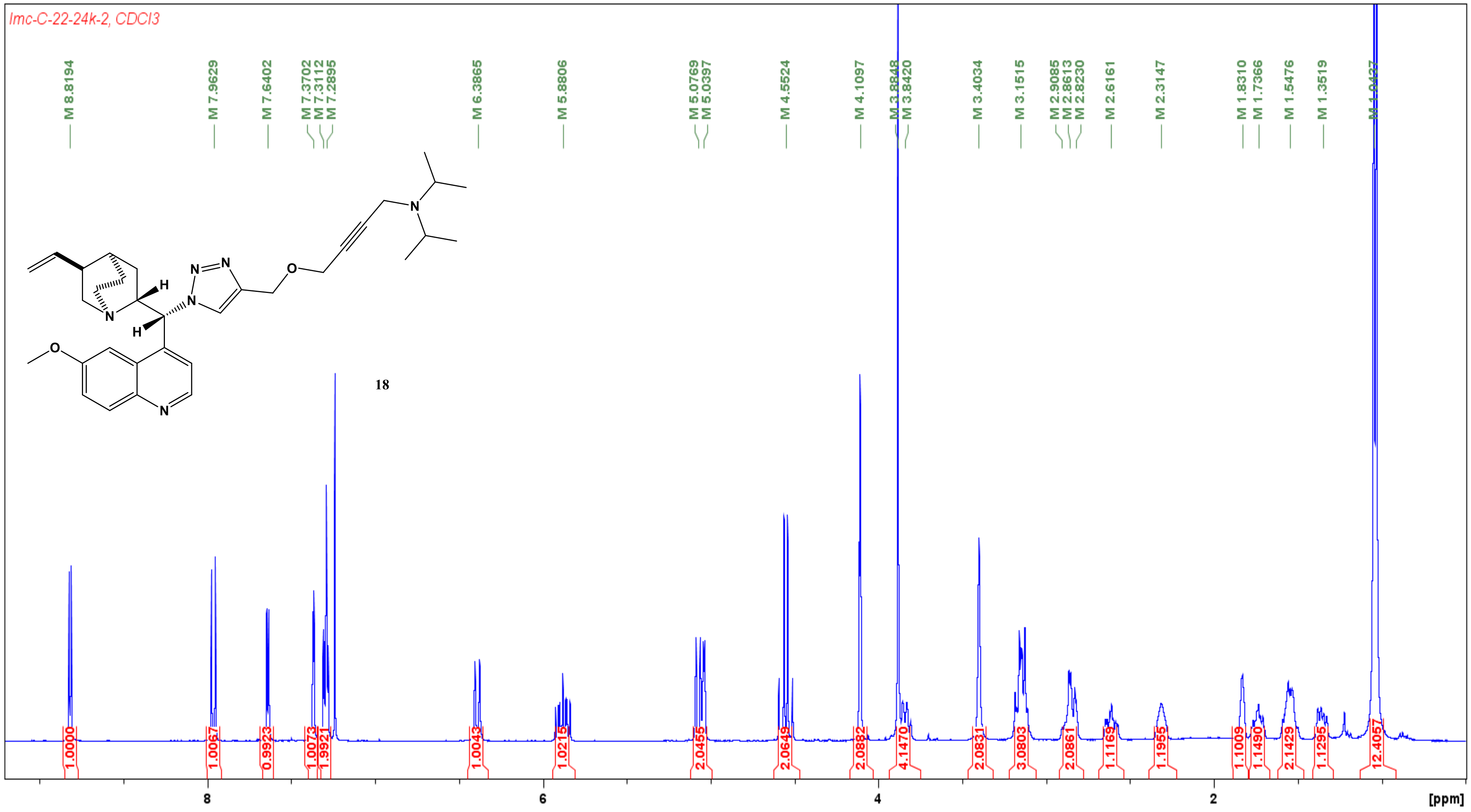

Figure S25. ¹H NMR spectrum of 4-((1-((*R*)-(6-methoxyquinolin-4-yl)((2*R*,4*S*,5*R*)-5-vinylquinuclidin-2-yl)methyl)-1*H*-1,2,3-triazol-4-yl)methoxy)-N,N-diisopropylbut-2-yn-1-amine (**18**) ($CDCl_3$, 400 MHz).

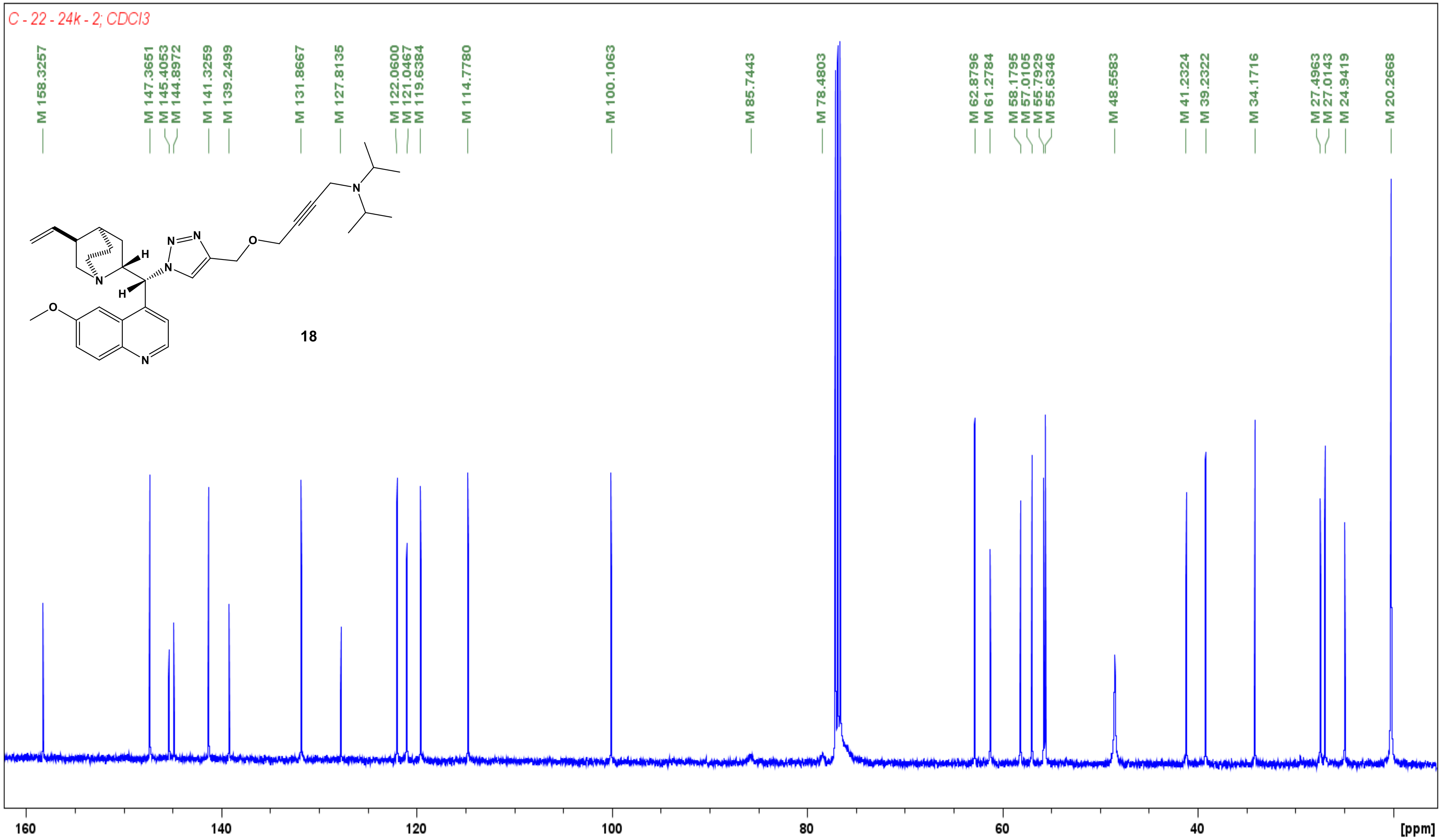

Figure S26. ^{13}C NMR spectrum of 4-((1-((*R*)-(6-methoxyquinolin-4-yl)((2*R*,4*S*,5*R*)-5-vinylquinuclidin-2-yl)methyl)-1*H*-1,2,3-triazol-4-yl)methoxy)-N,N-diisopropylbut-2-yn-1-amine (**18**) ($CDCl_3$, 126 MHz).

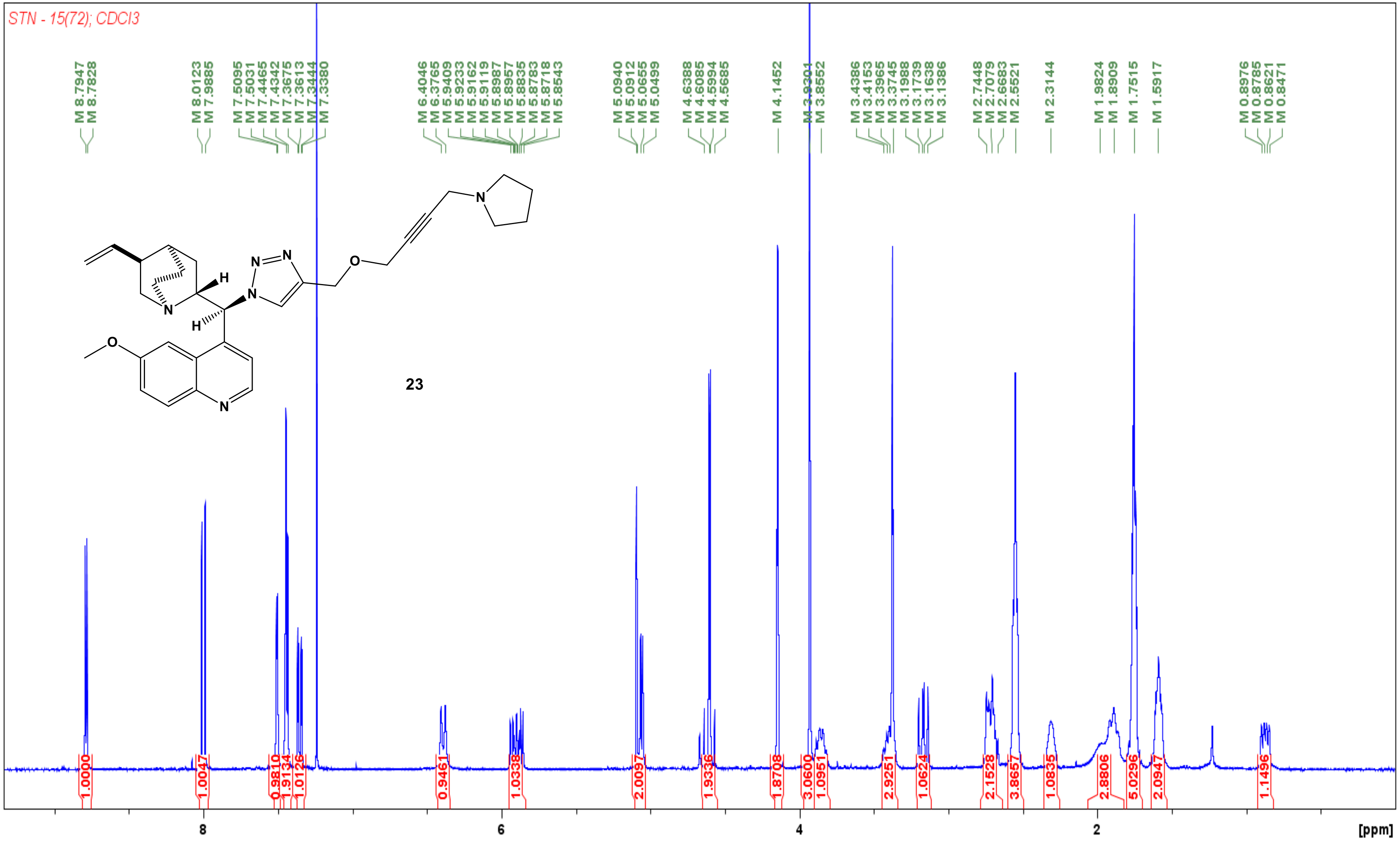

Figure S27. ^{1}H NMR spectrum of (2*R*,4*S*,5*R*)-2-((*S*)-(6-methoxyquinolin-4-yl)(4-((4-(pyrrolidin-1-yl)but-2-ynyloxy)methyl)-1*H*-1,2,3-triazol-1-yl)methyl)-5-vinylquinuclidine (**23**) ($CDCl_3$, 400 MHz).

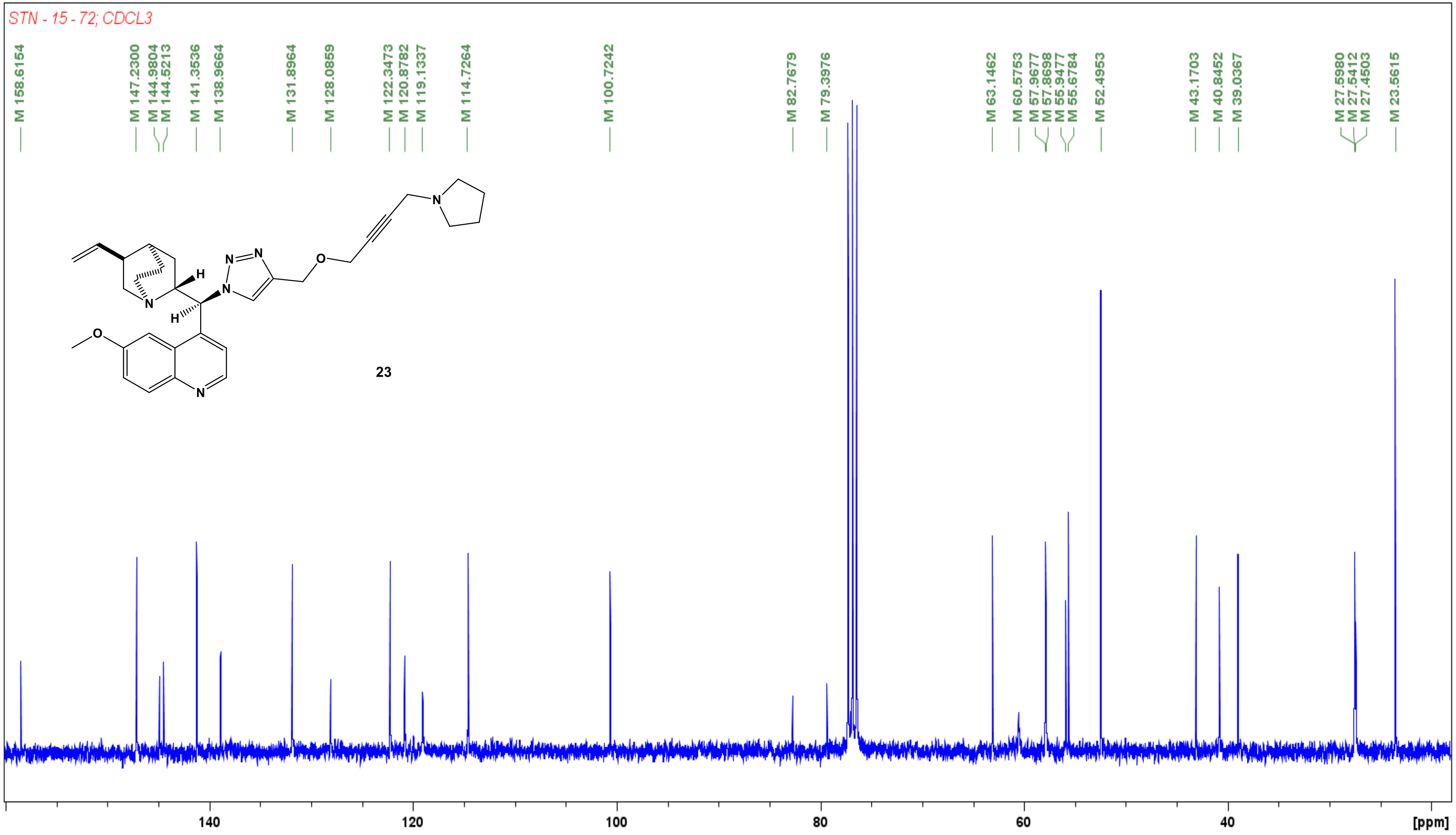

Figure S28. ^{13}C NMR spectrum of (2*R*,4*S*,5*R*)-2-((*S*)-(6-methoxyquinolin-4-yl)(4-((4-(pyrrolidin-1-yl)but-2-ynyloxy)methyl)-1*H*-1,2,3-triazol-1-yl)methyl)-5-vinylquinuclidine (**23**) ($CDCl_3$, 101 MHz).

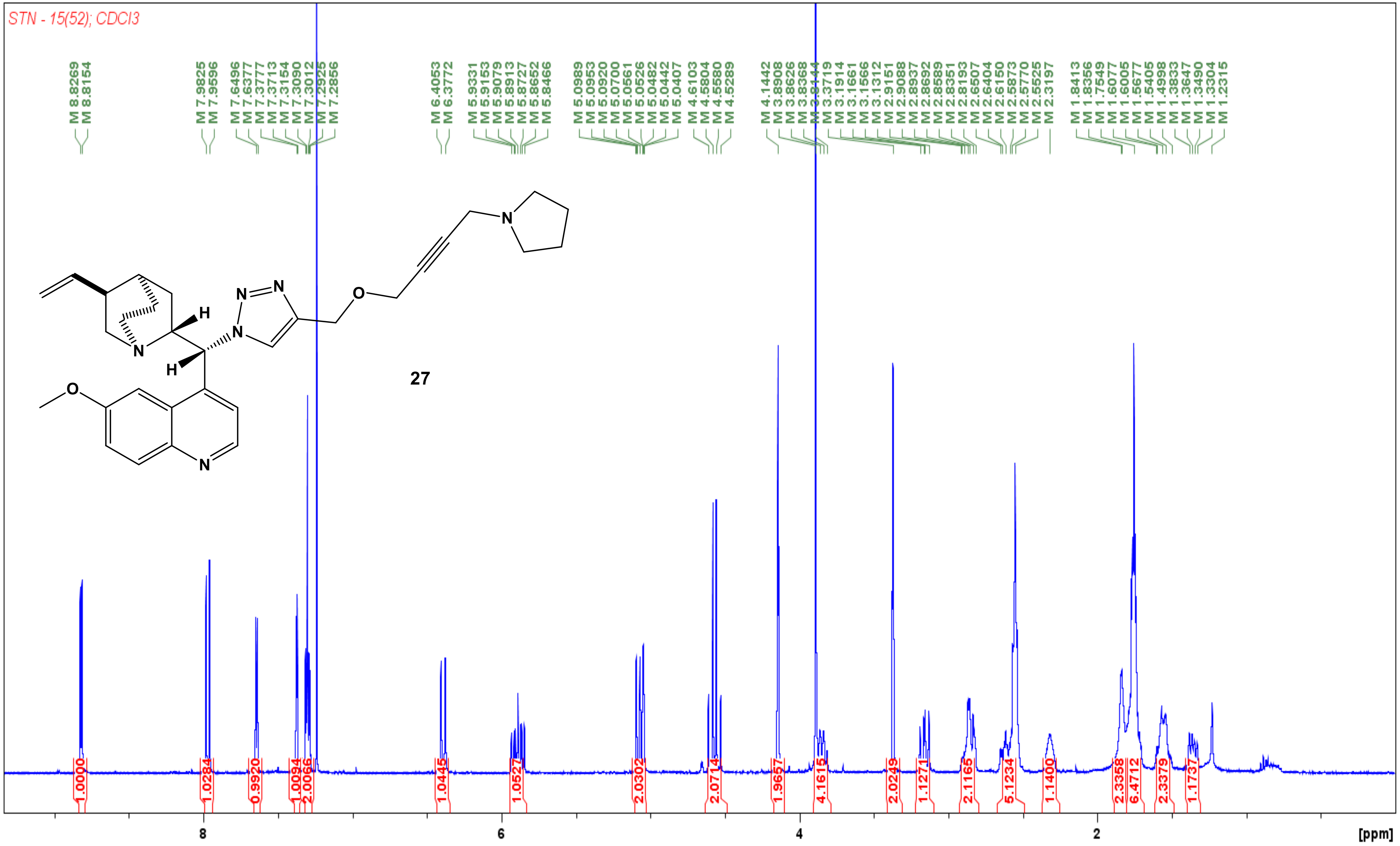

Figure S29. ^{1}H NMR spectrum of (2*R*,4*S*,5*R*)-2-((*R*)-(6-methoxyquinolin-4-yl)(4-((4-(pyrrolidin-1-yl)but-2-ynyloxy)methyl)-1*H*-1,2,3-triazol-1-yl)methyl)-5-vinylquinuclidine (**27**) ($CDCl_3$, 400 MHz).

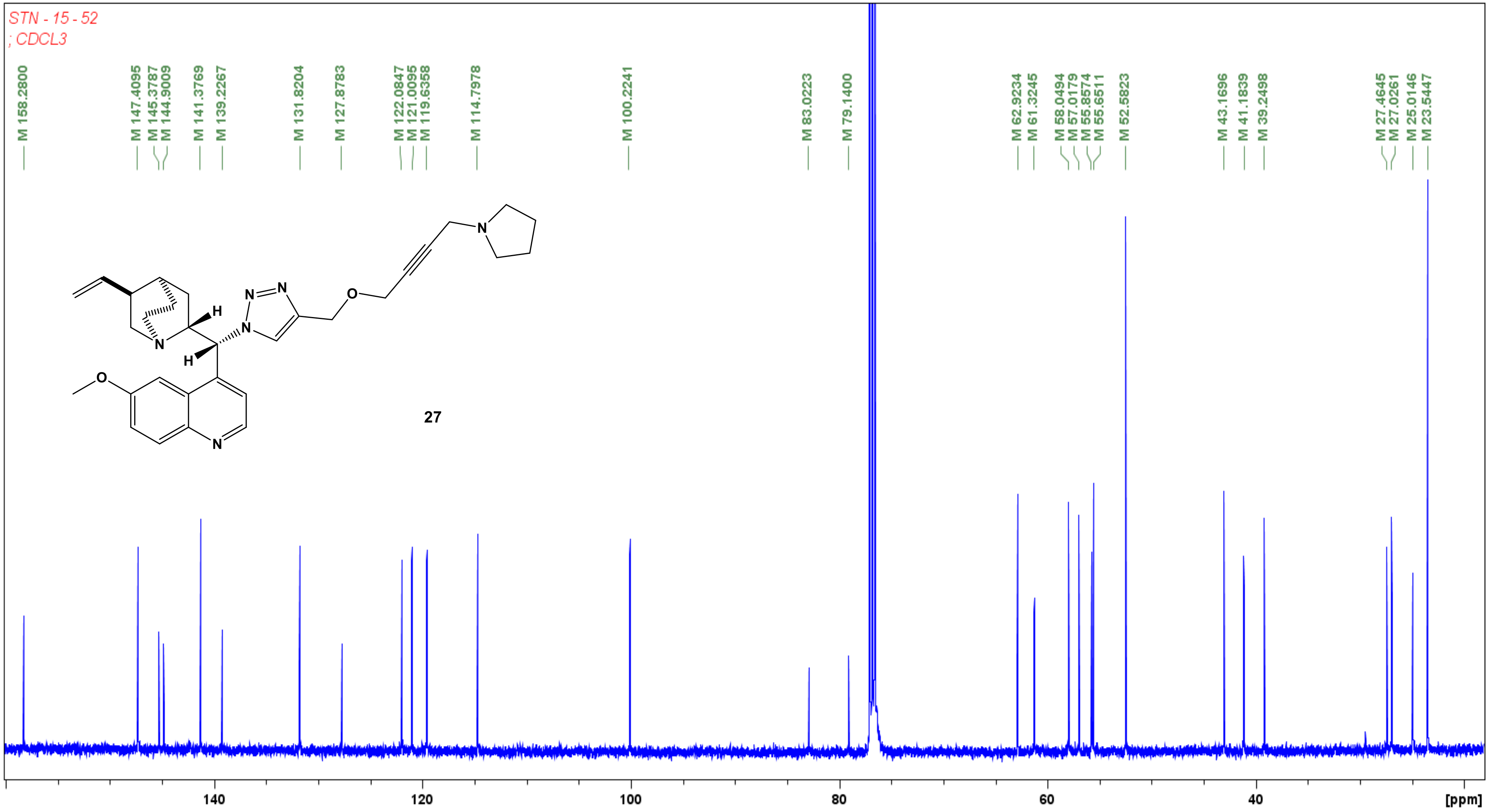

Figure S30. ^{13}C NMR spectrum of (2*R*,4*S*,5*R*)-2-((*R*)-(6-methoxyquinolin-4-yl)(4-((4-(pyrrolidin-1-yl)but-2-ynyloxy)methyl)-1*H*-1,2,3-triazol-1-yl)methyl)-5-vinylquinuclidine (**27**) ($CDCl_3$, 126 MHz).

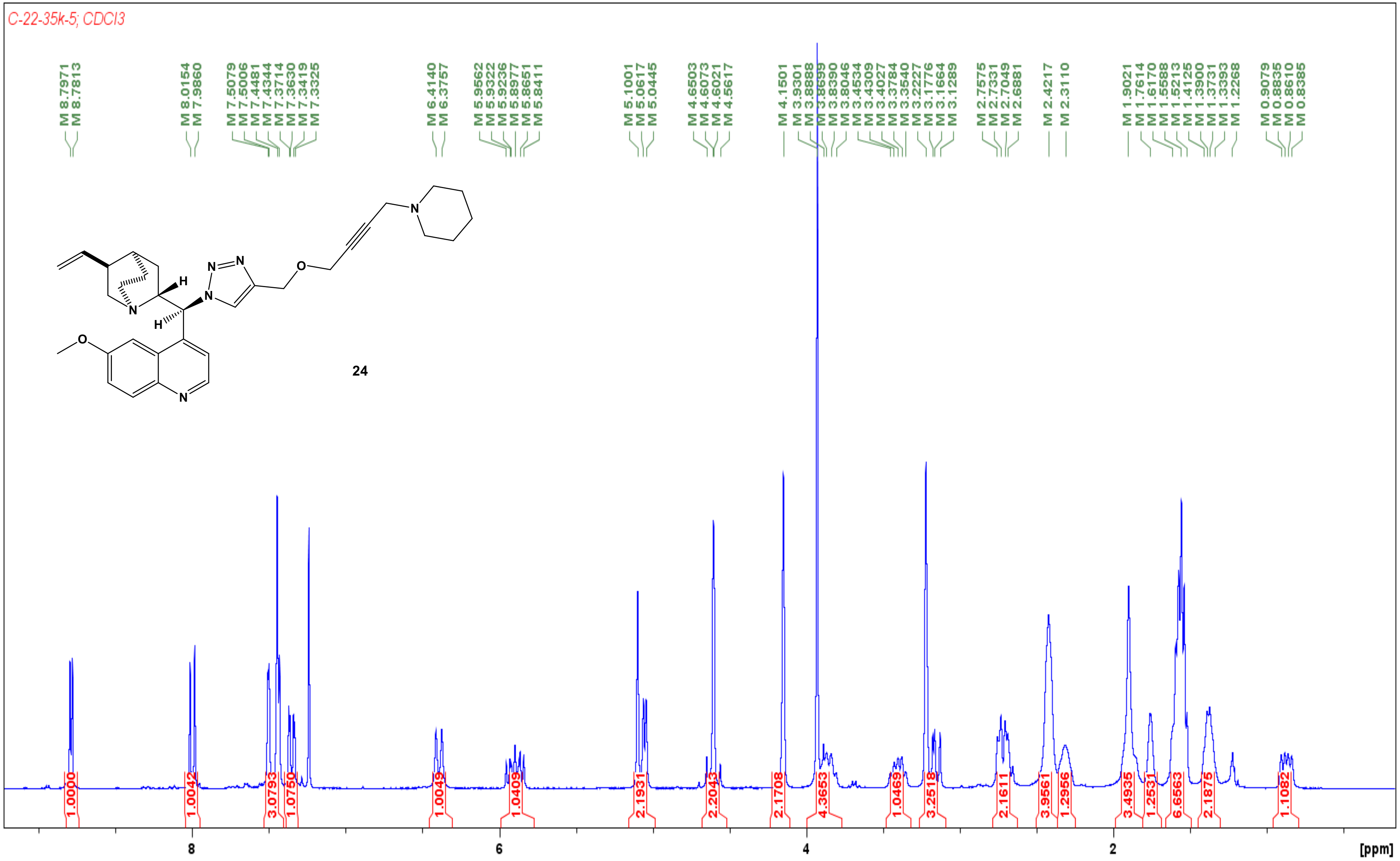

Figure S31. ¹H NMR spectrum of (2*R*,4*S*,5*R*)-2-((*S*)-(6-methoxyquinolin-4-yl)(4-((4-(*piperidin*-1-yl)but-2-ynyloxy)methyl)-1*H*-1,2,3-triazol-1-yl)methyl)-5-vinylquinuclidine (**24**) ($CDCl_3$, 300 MHz).

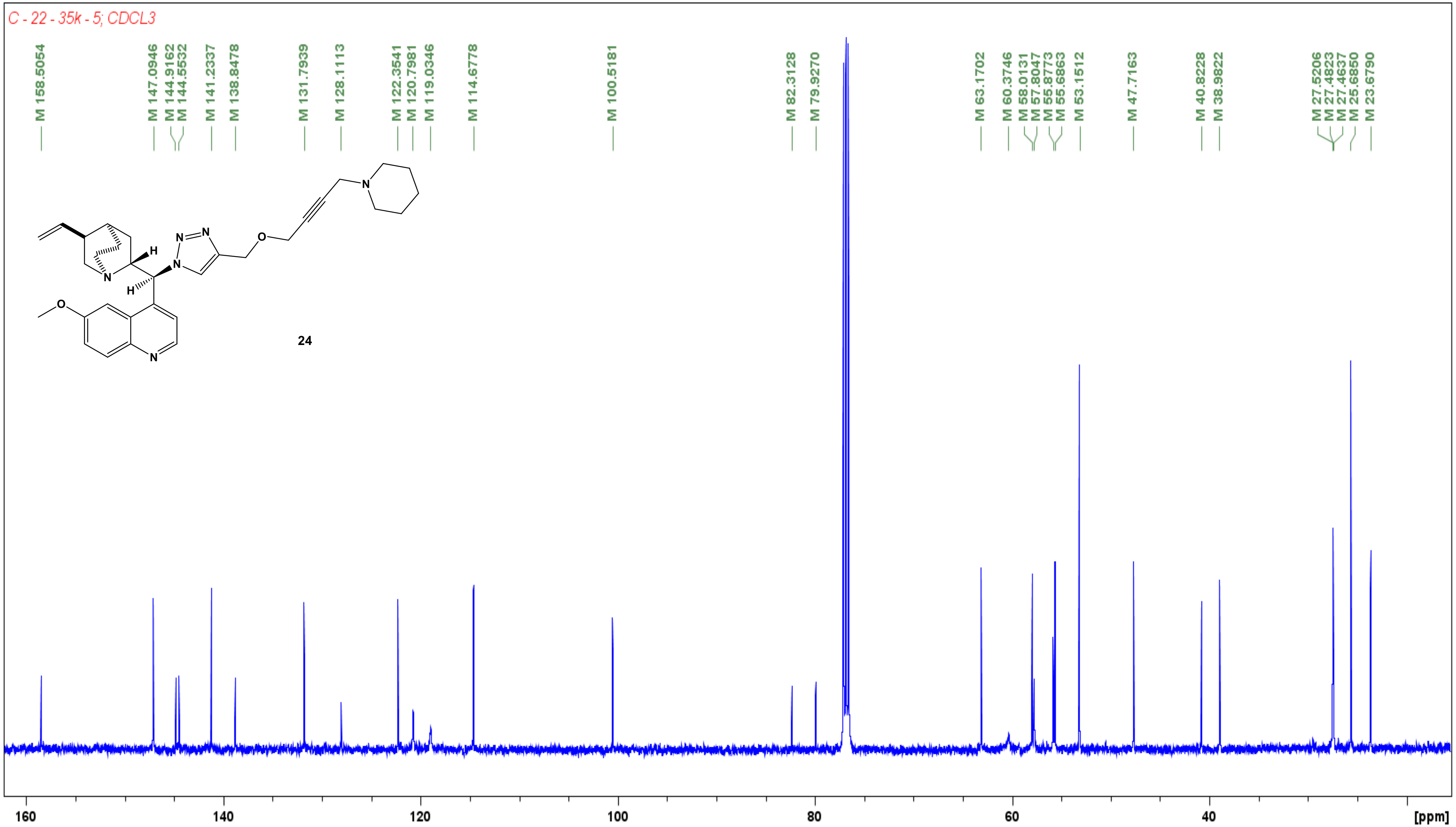

Figure S32. ^{13}C NMR spectrum of (2*R*,4*S*,5*R*)-2-((*S*)-(6-methoxyquinolin-4-yl)(4-((4-(*piperidin*-1-yl)but-2-ynyloxy)methyl)-1*H*-1,2,3-triazol-1-yl)methyl)-5-vinylquinuclidine (**24**) ($CDCl_3$, 126 MHz).

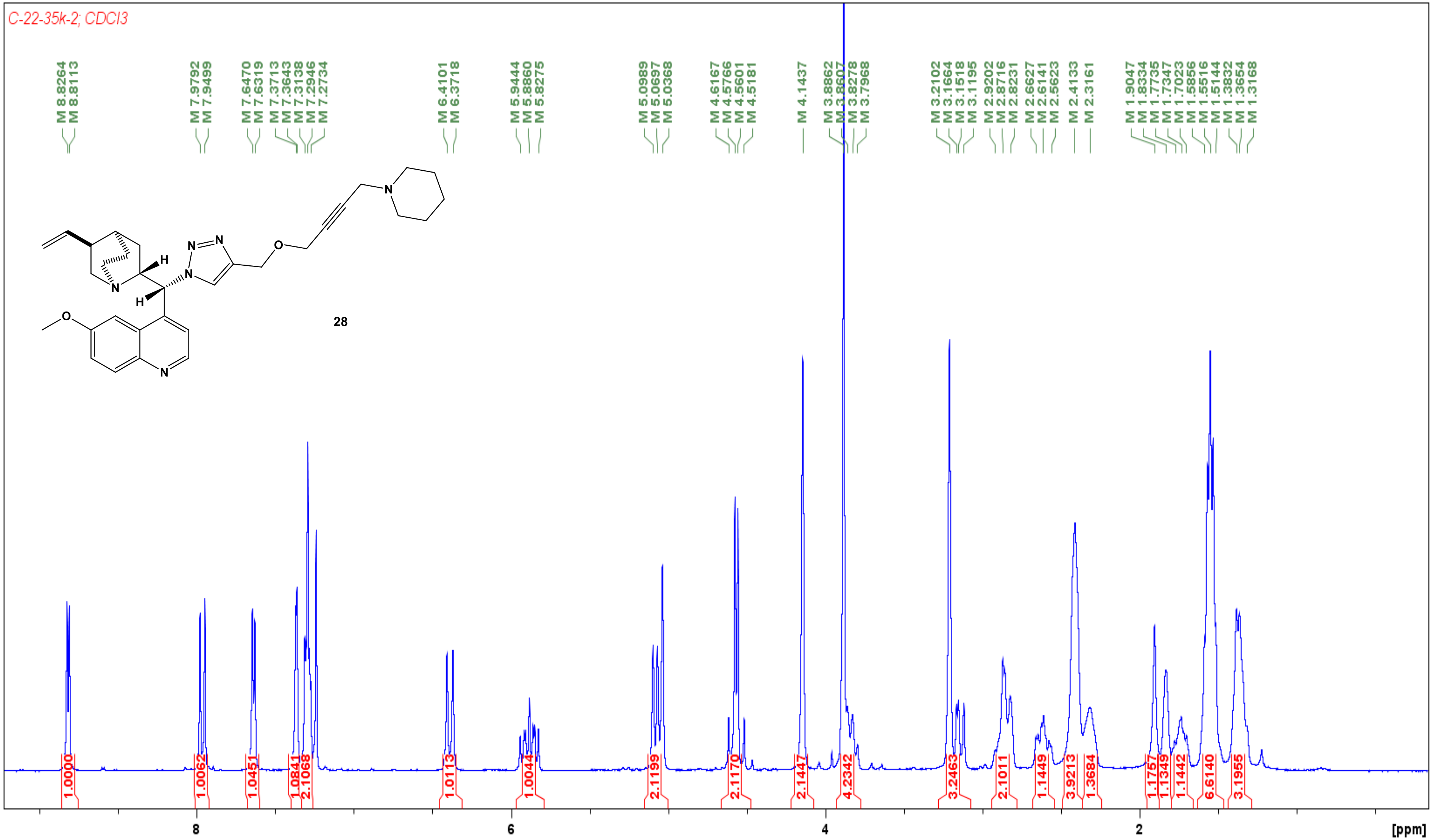

Figure S33. ¹H NMR spectrum of (2*R*,4*S*,5*R*)-2-((*R*)-(6-methoxyquinolin-4-yl)(4-((4-(*piperidin*-1-yl)but-2-ynyloxy)methyl)-1*H*-1,2,3-triazol-1-yl)methyl)-5-vinylquinuclidine (**28**) ($CDCl_3$, 300 MHz).

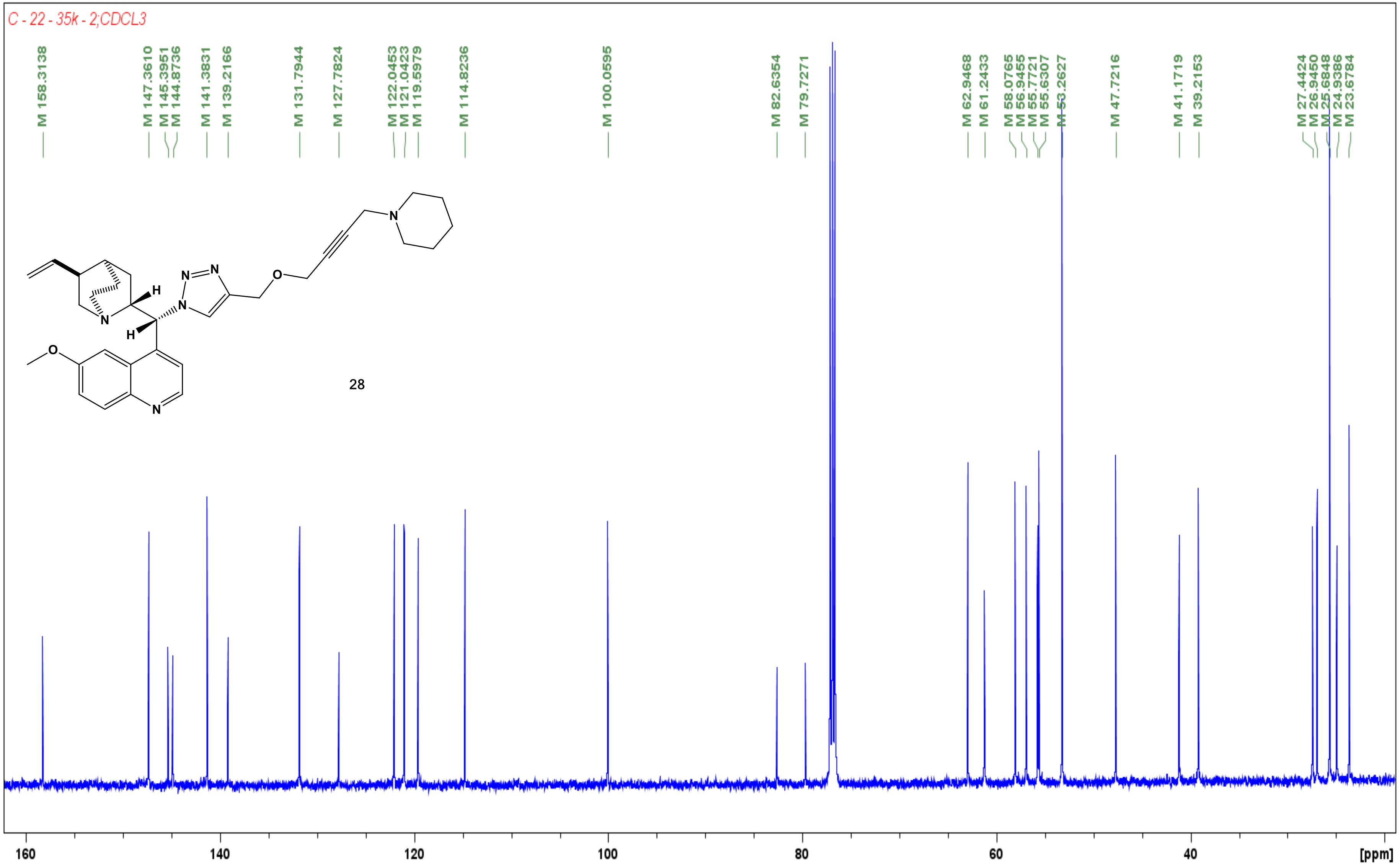

Figure S34. ^{13}C NMR spectrum of (2*R*,4*S*,5*R*)-2-((*R*)-(6-methoxyquinolin-4-yl)(4-((4-(*piperidin*-1-yl)but-2-ynyloxy)methyl)-1*H*-1,2,3-triazol-1-yl)methyl)-5-vinylquinuclidine (**28**) ($CDCl_3$, 126 MHz).

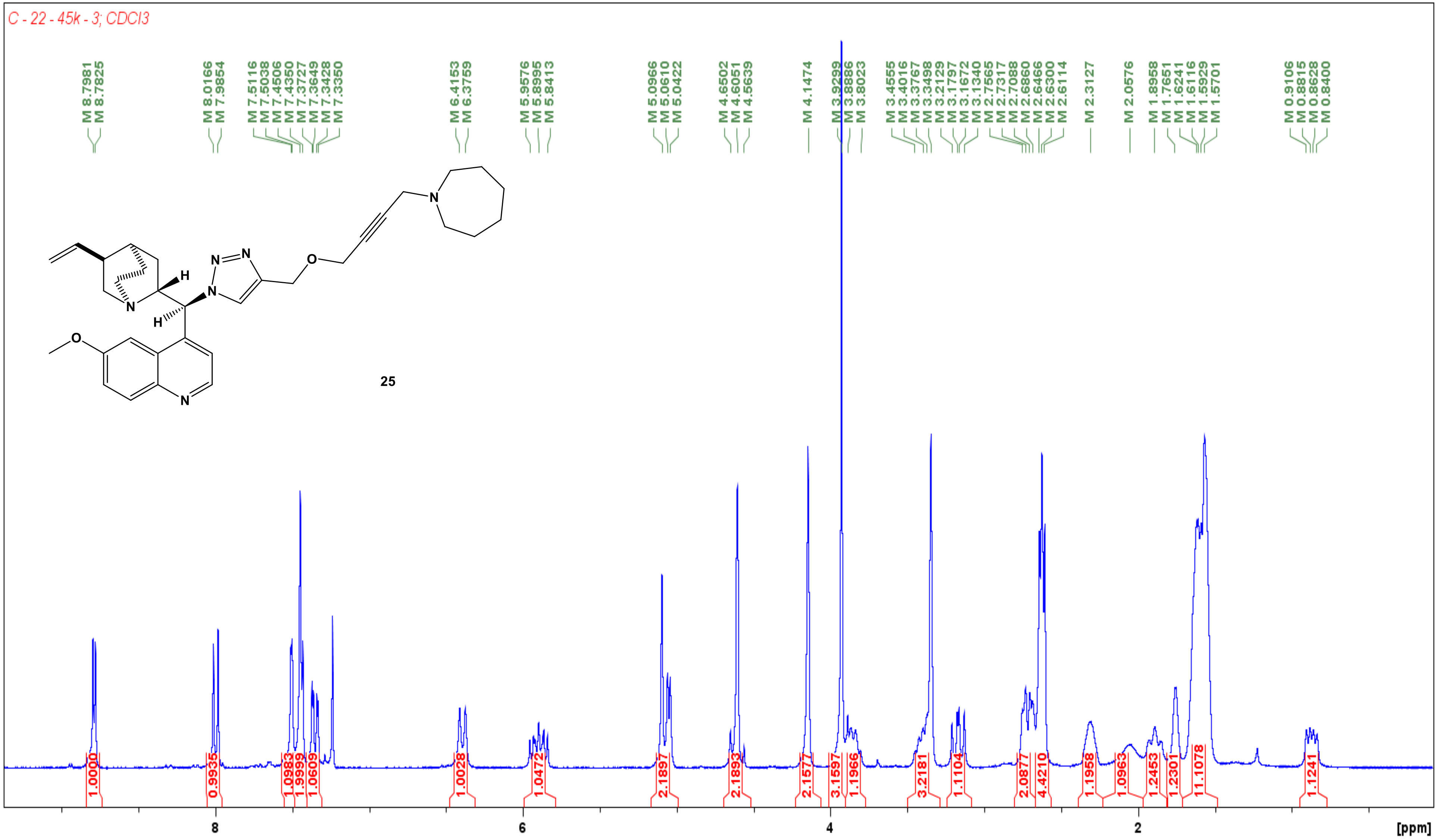

Figure S35. ¹H NMR spectrum of (2*R*,4*S*,5*R*)-2-((*S*)-(4-((4-(azepan-1-yl)but-2-ynyloxy)methyl)-1*H*-1,2,3-triazol-1-yl)(6-methoxyquinolin-4-yl)methyl)-5-vinylquinuclidine (**25**) ($CDCl_3$, 300 MHz)

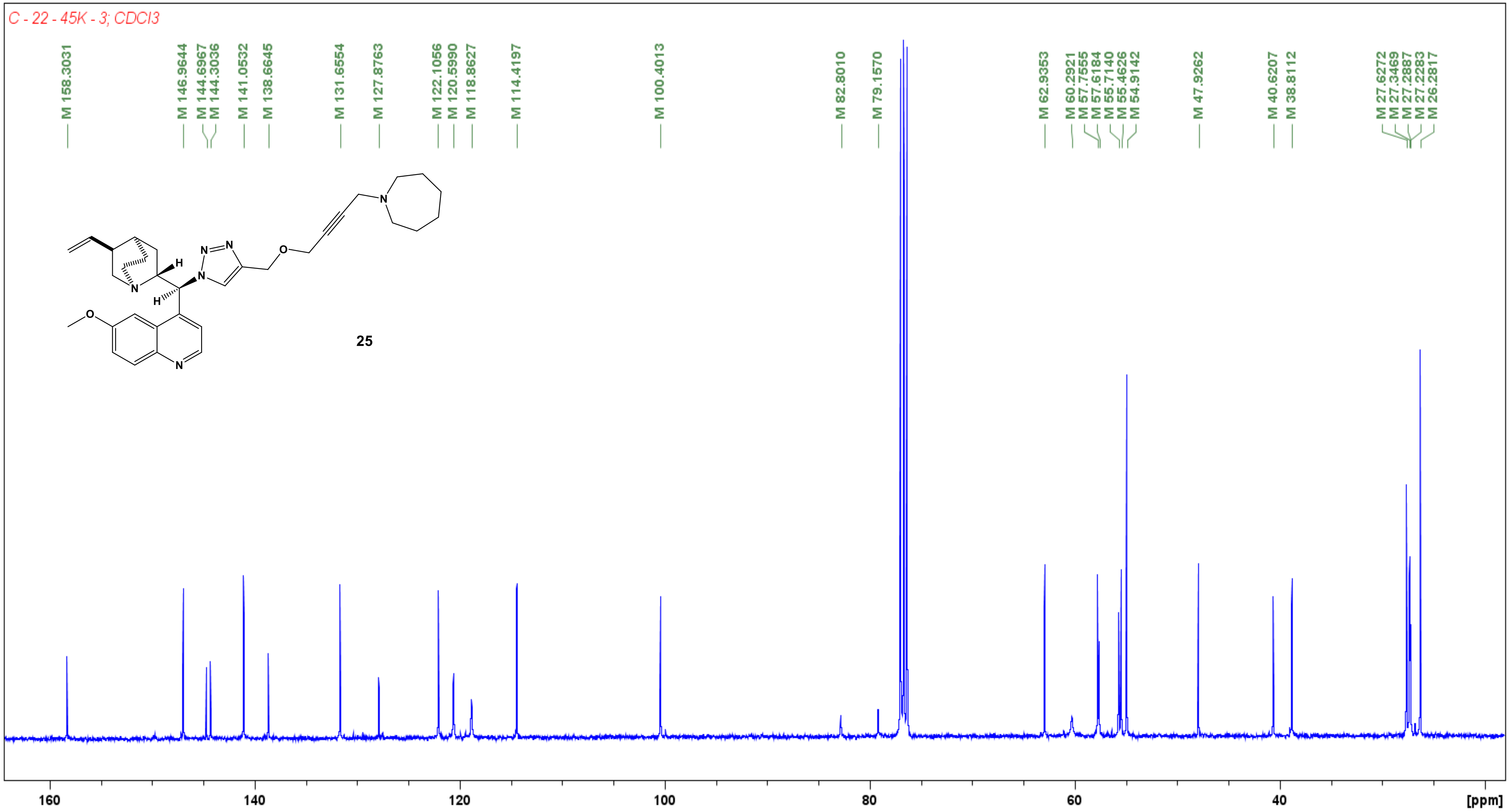

Figure S36. ^{13}C NMR spectrum of (2*R*,4*S*,5*R*)-2-((*S*)-(4-((4-(azepan-1-yl)but-2-ynyloxy)methyl)-1*H*-1,2,3-triazol-1-yl)(6-methoxyquinolin-4-yl)methyl)-5-vinylquinuclidine (**25**) ($CDCl_3$, 101 MHz).

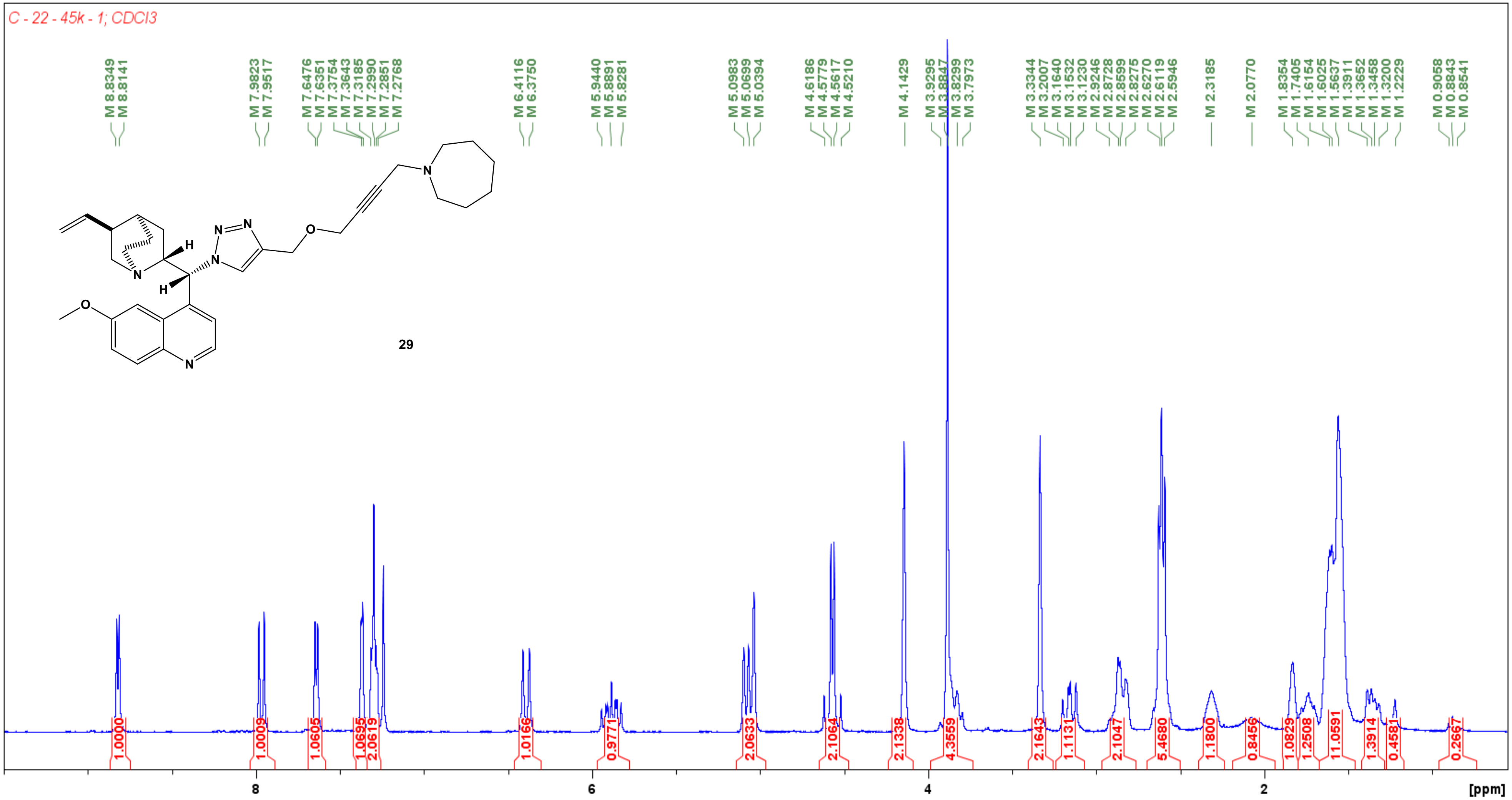

Figure S37. ^{1}H NMR spectrum of (2*R*,4*S*,5*R*)-2-((*R*)-(4-((4-(azepan-1-yl)but-2-ynyloxy)methyl)-1*H*-1,2,3-triazol-1-yl)(6-methoxyquinolin-4-yl)methyl)-5-vinylquinuclidine (**29**) ($CDCl_3$, 300 MHz).

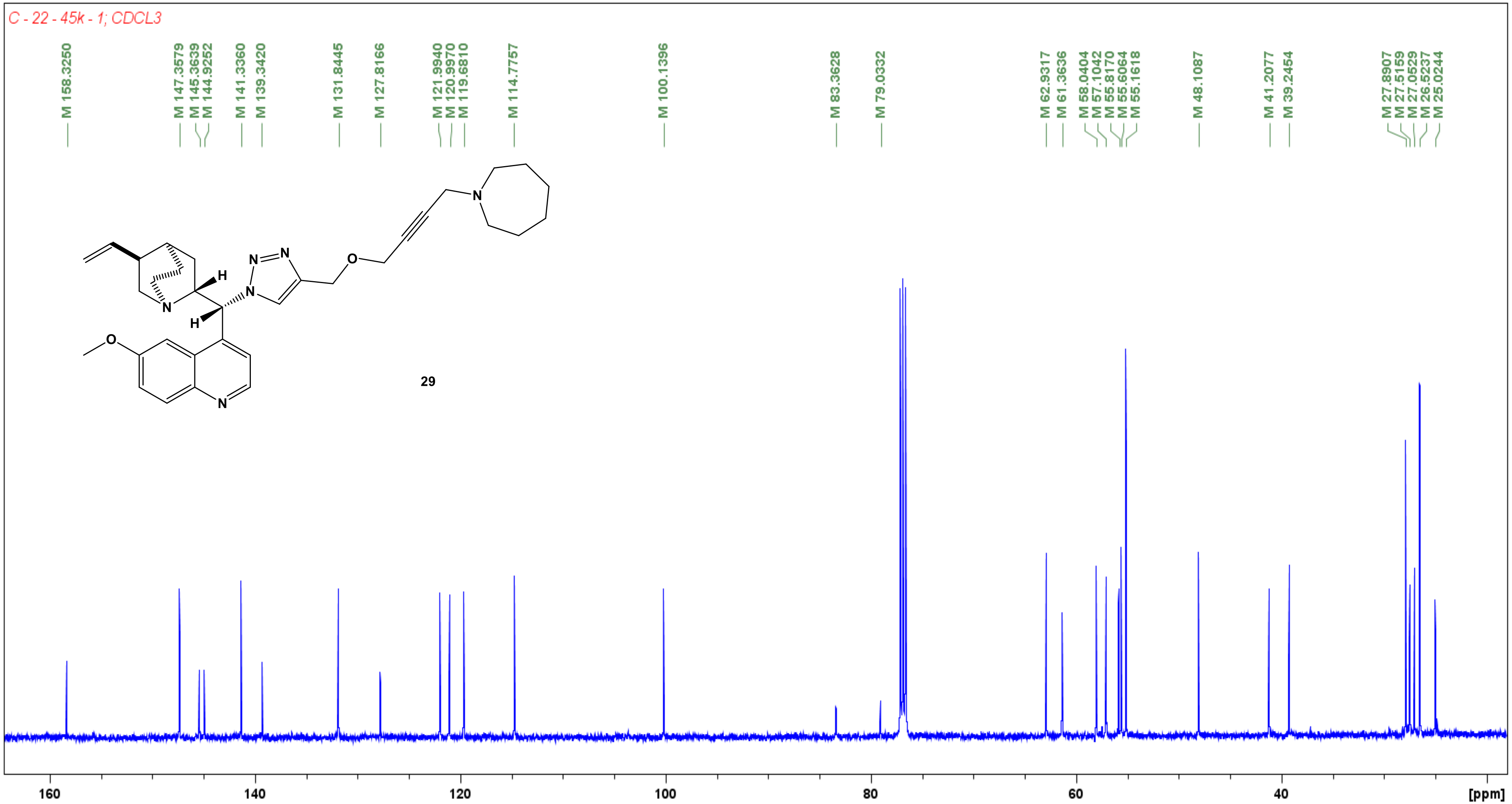

Figure S38. ^{13}C NMR spectrum of (2*R*,4*S*,5*R*)-2-((*R*)-(4-((4-(azepan-1-yl)but-2-ynyloxy)methyl)-1*H*-1,2,3-triazol-1-yl)(6-methoxyquinolin-4-yl)methyl)-5-vinylquinuclidine (**29**) ($CDCl_3$, 126 MHz).

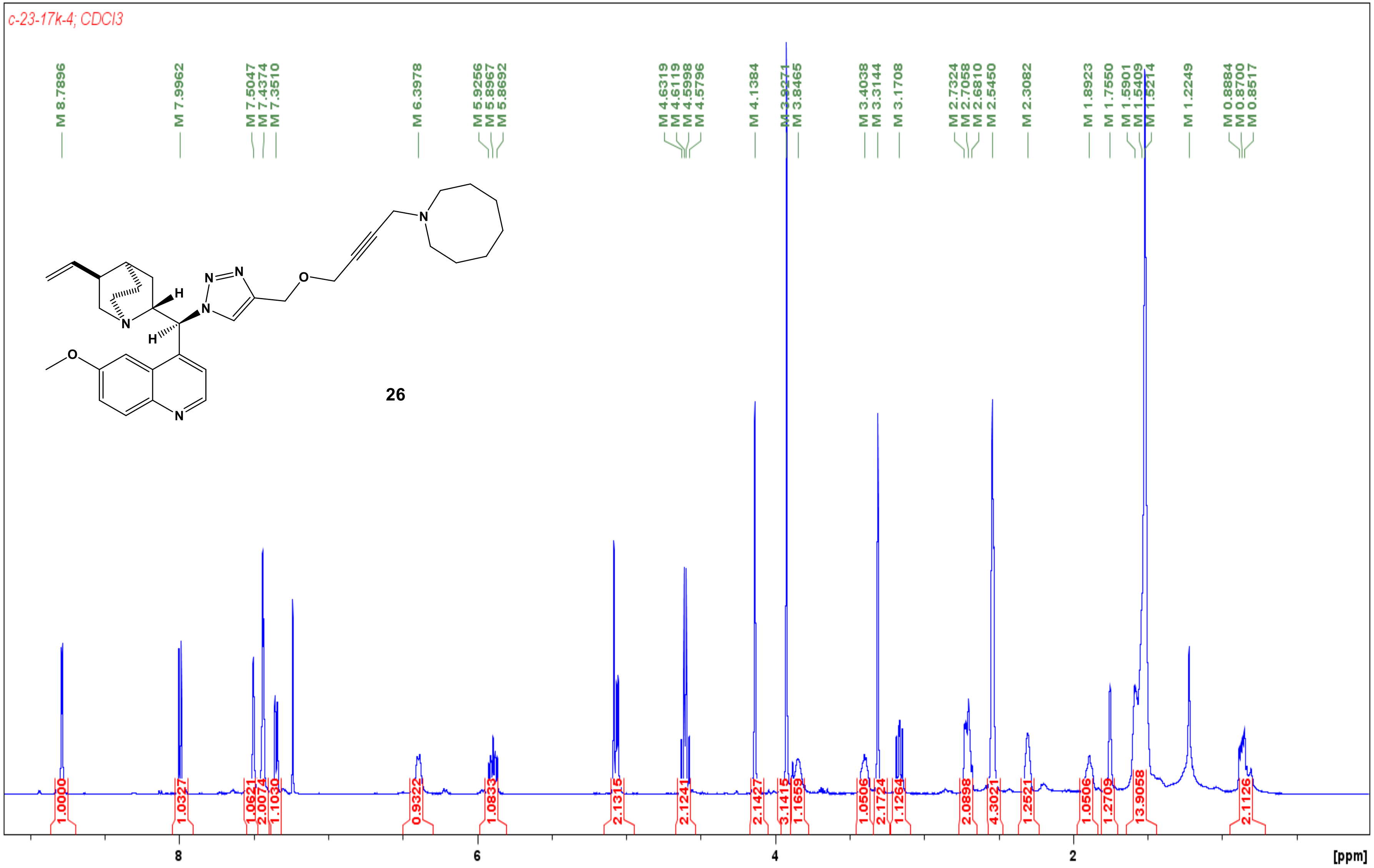

Figure S39. ¹H NMR spectrum of (2*R*,4*S*,5*R*)-2-((*S*)-(4-((4-(azocan-1-yl)but-2-ynyloxy)methyl)-1*H*-1,2,3-triazol-1-yl)(6-methoxyquinolin-4-yl)methyl)-5-vinylquinuclidine (**26**) ($CDCl_3$, 400 MHz).

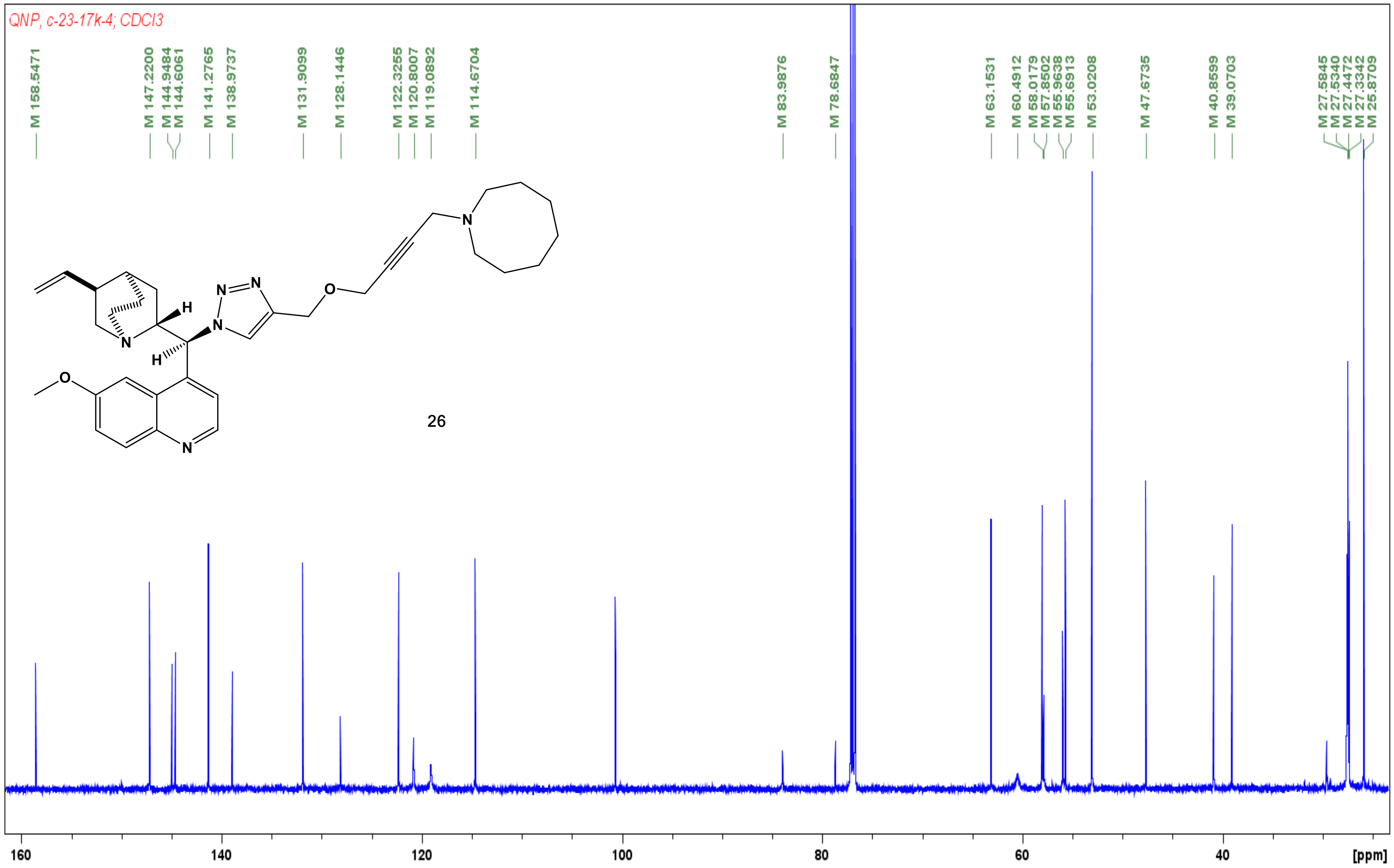

Figure S40. ^{13}C NMR spectrum of (2*R*,4*S*,5*R*)-2-((*S*)-(4-((4-(azocan-1-yl)but-2-ynyloxy)methyl)-1*H*-1,2,3-triazol-1-yl)(6-methoxyquinolin-4-yl)methyl)-5-vinylquinuclidine (**26**) ($CDCl_3$, 75 MHz).

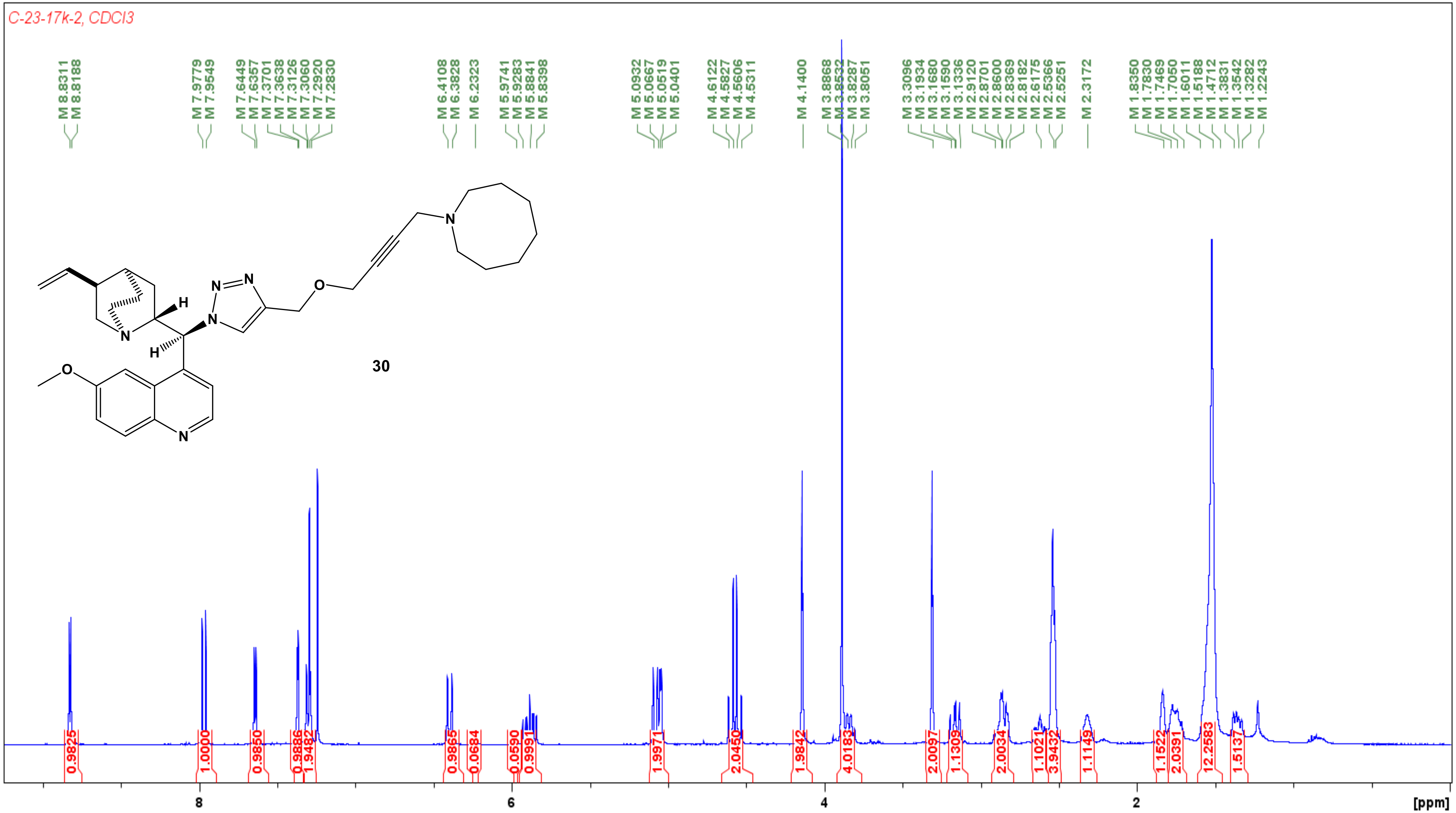

Figure S41. ^{1}H NMR spectrum of (2*R*,4*S*,5*R*)-2-((*R*)-(4-((4-(azocan-1-yl)but-2-ynyloxy)methyl)-1*H*-1,2,3-triazol-1-yl)(6-methoxyquinolin-4-yl)methyl)-5-vinylquinuclidine (**30**) ($CDCl_3$, 400 MHz).

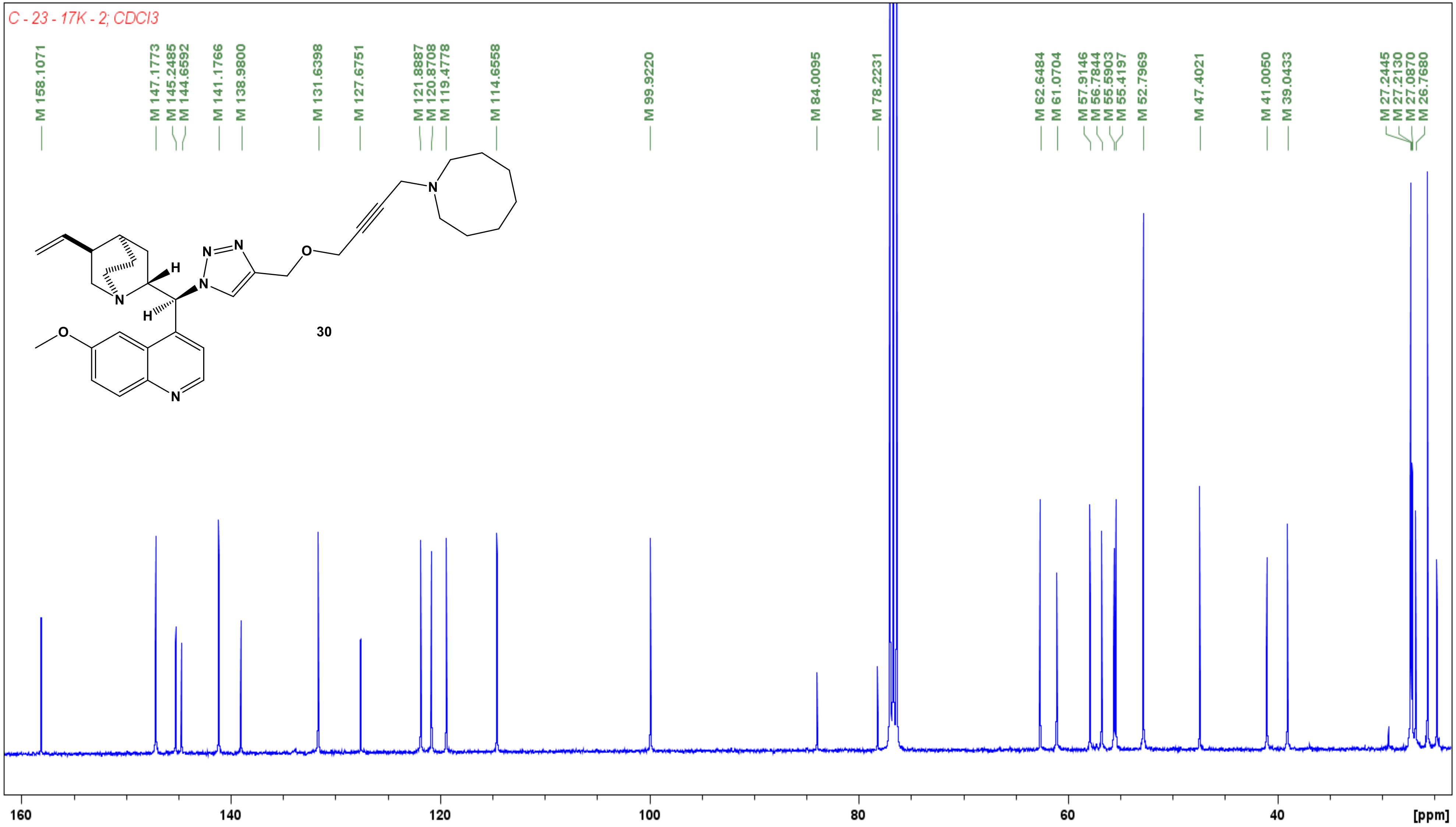

Figure S42. ^{13}C NMR spectrum of (2*R*,4*S*,5*R*)-2-((*R*)-(4-((4-(azocan-1-yl)but-2-ynyloxy)methyl)-1*H*-1,2,3-triazol-1-yl)(6-methoxyquinolin-4-yl)methyl)-5-vinylquinuclidine (**30**) ($CDCl_3$, 100 MHz).
